# Proteorhodopsin insights into the molecular mechanism of vectorial proton transport

**DOI:** 10.1126/sciadv.adu5303

**Published:** 2025-04-16

**Authors:** Sergey Bukhdruker, Ivan Gushchin, Vitaly Shevchenko, Kirill Kovalev, Vitaly Polovinkin, Fedor Tsybrov, Roman Astashkin, Alexey Alekseev, Anatoly Mikhaylov, Siarhei Bukhalovich, Dmitry Bratanov, Yury Ryzhykau, Daria Kuklina, Nicolas Caramello, Tatyana Rokitskaya, Yuri Antonenko, Maksim Rulev, Chavdar Stoev, Dmitrii Zabelskii, Ekaterina Round, Andrey Rogachev, Valentin Borshchevskiy, Rohit Ghai, Gleb Bourenkov, Mahel Zeghouf, Jacqueline Cherfils, Martin Engelhard, Igor Chizhov, Francisco Rodriguez-Valera, Ernst Bamberg, Valentin Gordeliy

**Affiliations:** ^1^ Research Center for Molecular Mechanisms of Aging and Age-Related Diseases, Moscow Institute of Physics and Technology, 141700 Dolgoprudny, Russia.; ^2^ Hamburg Outstation c/o DESY, European Molecular Biology Laboratory, 22607 Hamburg, Germany.; ^3^ ELI Beamlines Centre, ELI ERIC, 252 41 Dolní Břežany, Czechia.; ^4^ Institut de Biologie Structurale J.-P. Ebel, Université Grenoble Alpes-CEA-CNRS, 38000 Grenoble, France.; ^5^ Frank Laboratory of Neutron Physics, Joint Institute for Nuclear Research, 141980 Dubna, Russia.; ^6^ Laboratory of Structural Dynamics, Stability and Folding of Proteins, Institute of Cytology, Russian Academy of Sciences, 194064 St. Petersburg, Russia.; ^7^ Institute for Nanostructure and Solid State Physics, HARBOR, Universität Hamburg, 22761 Hamburg, Germany.; ^8^ Belozersky Institute of Physico-Chemical Biology, Lomonosov Moscow State University, 119991 Moscow, Russia.; ^9^ Department of Cell and Molecular Biology, Biomedical Centre, Uppsala University, 75124 Uppsala, Sweden.; ^10^ European X-ray Free Electron Laser GmbH, 22869 Schenefeld, Germany.; ^11^ Department of Aquatic Microbial Ecology, Institute of Hydrobiology, Biology Centre of the Czech Academy of Sciences, 370 05 České Budějovice, Czech Republic.; ^12^ Université Paris-Saclay, CNRS, and Ecole Normale Supérieure Paris-Saclay, 91190 Gif-sur-Yvette, France.; ^13^ Department Structural Biochemistry, Max Planck Institute of Molecular Physiology, 44227 Dortmund, Germany.; ^14^ Institute for Biophysical Chemistry, Medizinische Hochschule Hannover, D-30625 Hannover, Germany.; ^15^ Evolutionary Genomics Group, Departamento de Producción Vegetal y Microbiología, Universidad Miguel Hernández, San Juan de Alicante, 03550 Alicante, Spain.; ^16^ Department of Biophysical Chemistry, Max Planck Institute of Biophysics, 60438 Frankfurt am Main, Germany.

## Abstract

Bacterial proton pumps, proteorhodopsins (PRs), are a major group of light-driven membrane proteins found in marine bacteria. They are functionally and structurally distinct from archaeal and eukaryotic proton pumps. To elucidate the proton transfer mechanism by PRs and understand the differences to nonbacterial pumps on a molecular level, high-resolution structures of PRs’ functional states are needed. In this work, we have determined atomic-resolution structures of MAR, a PR from marine actinobacteria, in various functional states, notably the challenging late O intermediate state. These data and information from recent atomic-resolution structures on an archaeal outward proton pump bacteriorhodopsin and bacterial inward proton pump xenorhodopsin allow for deducing key universal elements for light-driven proton pumping. First, long hydrogen-bonded chains characterize proton pathways. Second, short hydrogen bonds allow proton storage and inhibit their backflow. Last, the retinal Schiff base is the active proton donor and acceptor to and from hydrogen-bonded chains.

## Teaser

Bacteria aptly use fundamental mechanisms for light-driven proton transfer across a membrane.

## INTRODUCTION

Proteorhodopsins (PRs) are the largest family of light-driven bacterial proton pumps that enable their hosts to perform phototrophic metabolism (*1*, *2*). They are evolutionarily distant from archaeal (*3*) and eukaryotic [the latter are similar to archaeal (*4*)] rhodopsin proton pumps, having specific structural and functional features. In PRs, in contrast to archaeal or eukaryotic proton pumps, the proton uptake from the cytoplasm precedes the proton release to the extracellular space, and the pumping is pH dependent (*5*). Also, PRs lack the proton storage (release) pocket characteristic of archaeal and eukaryotic pumps, such as the PRG group (Glu^194^-Glu^204^) in the archaeal outward light-driven proton pump bacteriorhodopsin from *Halobacterium salinarum* [*Hs*BR (*6*)]. Instead, a water pore in the extracellular part penetrates deeply inside the protein and approaches the conserved arginine residue corresponding to Arg^82^ in *Hs*BR (*7*). Last, it was suggested that the primary proton acceptor in PRs is a conserved His-Asp pair instead of a lone Asp^85^ residue in *Hs*BR (*8*, *9*). All these differences indicate that proton transfer by this abundant protein family may differ from what is established for archaeal and eukaryotic proton pumps (*3*). As PRs are the largest and ecologically important family of microbial rhodopsins (MRhs), which substantially contribute to life in the ocean and may play a critical role in global ecology and climate regulation, understanding the molecular mechanisms of PRs would be of fundamental importance. The differences between archaeal/eukaryotic and bacterial proton pumps might provide insights into the evolutionary aspects of the structure and function of the transporters. Whether the fundamentals of proton pumping are universal across all life domains is an intriguing question, the answer to which could help to elucidate possible common underlying elements. Understanding the fundamentals of proton transfer and identifying its key elements will likely promote unraveling the mechanisms for other types of proton transporters.

Two substantial obstacles prevented such an analysis. First, no structures of the photocycle intermediates of PRs are available. Despite 24 years of effort, even a high-resolution structure of the ground state of a PR under proton-pumping (high pH) conditions remains elusive. Because the function of the light-driven proton pumps depends on small alternations within the active site that change the lengths (and, thus, types) of hydrogen bonds (H-bonds) and partially rely on water molecules and alternative conformations of the protein side chains (*3*), the data must be of exceptional quality. Second, a crystal structure of the last intermediate state, the O state, of any outward light-driven proton pump has not yet been obtained [here, it is important to note that the term “O state” in other MRhs can have a different meaning (*10*, *11*)]. In all domains of life, the proton transfer in the extracellular part in such pumps proceeds upon transition from the O to ground state after the retinal is relaxed back to the all-trans state [see Supplementary Text 1 and ref. (*12*)]. The O state is not observed in the crystals of *Hs*BR, possibly due to crystal confinement (*13*, *14*). However, even the N state of *Hs*BR, preceding the O state, is still under investigation because of the moderate resolution of the data (*15*). For this reason, it remains uncertain which pathways protons use to exit the pumps into the extracellular space at the last stage of the photocycle.

Here, we have functionally and structurally characterized a representative of the PR family found in a widely distributed clade of marine actinobacteria, *Candidatus* Actinomarina minuta, named MAR [marine actinobacterial rhodopsin or MacR (*16*)]. We show that MAR is a typical PR whose functionality depends on the pH of the environment. The 1.25-Å-resolution structure of the ground state of MAR obtained under proton-pumping, high pH conditions reveals pentagonal organization of the H-bonds in the retinal Schiff base (RSB) site, similar to what is known for *Hs*BR (*3*, *17*, *18*). The structures of key functional intermediates of a PR, namely N, O*, and O states, were solved. They are of the highest crystallographic quality: The resolutions are 2.30, 1.41, and 1.09 Å, respectively. The data are completed by the structure of the M-like intermediate state solved at a 1.60-Å resolution. These findings highlight notable differences between bacterial light-driven proton pumps on the one hand and archaeal and eukaryotic ones on the other hand. In particular, for MAR, we show the active participation of both residues, His^51^ and Asp^72^, in storing a proton from RSB, which substantially distinguishes it from *Hs*BR, where only Asp^85^ is the proton acceptor (*19*). Also, in MAR, much less hydration of the cytoplasmic part is required for reprotonation of the proton donor Glu^83^ during the N-to-O state transition compared to what is expected for reprotonation of Asp^96^ in *Hs*BR (*15*). However, fundamental features of the mechanisms remain common.

Our results, complementary to the recent data on archaeal outward proton pump *Hs*BR (*3*) and bacterial inward *Bacillus coahuilensis* xenorhodopsin proton pump [*Bc*XeR (*20*)], provide a unique opportunity to “visualize” experimentally the complete mechanism of proton transfer through membranes and propose a general concept of proton translocation. Although these results are promising, it should be noted that neutron and/or subatomic-resolution x-ray crystallography is still necessary to resolve protons and hydrogens, which are key for understanding the quantum mechanical nature of proton transfer and signaling. Moreover, additional experiments would be helpful to elucidate the influence of lipid environment and protein oligomerization on the structure-function parameters of these transporters (*21*).

## RESULTS

### MAR is a representative of PRs

An MRh gene was identified in the assembled metagenome of a species of marine actinobacteria, *Candidatus* Actinomarina minuta (*16*). The respective protein was named MacR (*16*, *22*). We will call it MAR to prevent confusion with eukaryotic LR/Mac rhodopsin described previously (*4*). Analysis of the phylogenetic tree of available MRhs (fig. S1) reveals that MAR is a representative of a clade of proteins belonging to the broad family of PR light-driven proton pumps. The MRh superfamily comprises two major clusters that are linked to archaeal (cluster A) and bacterial (cluster B) origin (fig. S1) (*4*, *23*). To avoid confusion, here and throughout the text, we use the term PRs only for light-driven proton pumps of bacterial origin (cluster B, excluding sodium and chloride pumps). Notably, sodium (NDQ rhodopsins) and chloride (NTQ rhodopsins) pumps are attributed to a different group of MRhs despite sharing a bacterial ancestor with PRs.

**Fig. 1.**
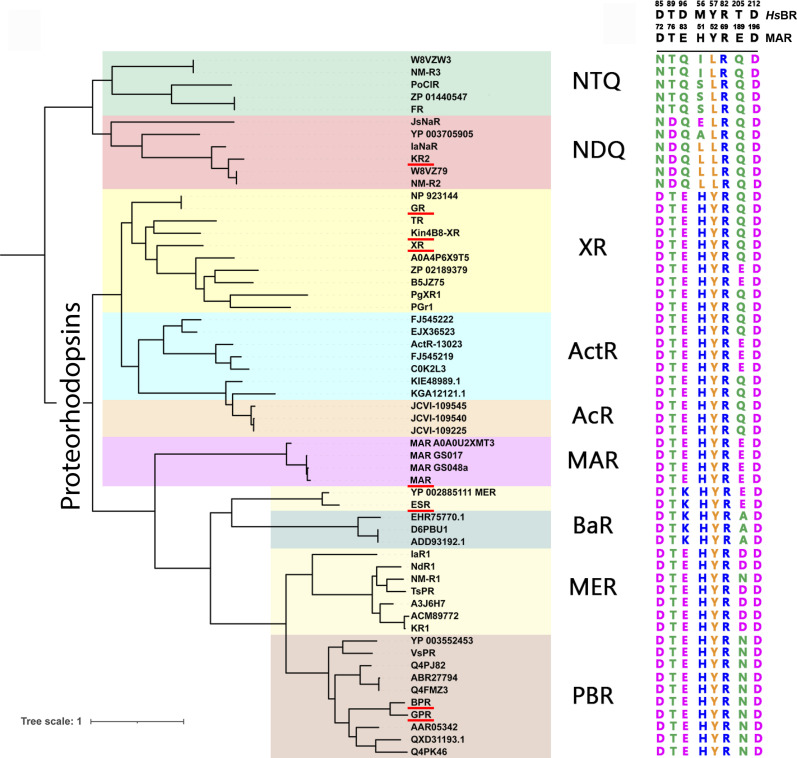
Sequence analysis of PRs in the context of cluster B rhodopsins. Phylogenetic tree of selected members of MRh families (*4*, *23*). The sodium pump (NDQ) and chloride pump (NTQ) families are included in the analysis. The PR subfamilies, XR, actinorhodopsin (ActR), acidinorhodopsin (AcR), MAR, *Bacteroidetes* rhodopsins (BaR), marine euryarcheal rhodopsins (MER), and proteobacterial rhodopsins (PBR), are labeled according to their abbreviations. Proteins discussed in the text are highlighted with red lines.

With its length of 220 amino acid residues, MAR is among the shortest and most compact MRhs, providing almost a minimalistic model for proton pumping. However, we should note that smaller proton pumps have been recently identified, such as Schizorhodopsin-4 with 202 amino acid residues (*24*). Detailed analysis of MAR’s sequence in the context of the cluster B rhodopsins reveals that MAR comprises all the amino acid residues characteristic of PRs. MAR has a His^51^-Asp^72^ pair, previously proposed as the proton acceptor (*9*), the proton donor Glu^83^, and other residues, including Tyr^52^, Arg^69^, Thr^76^, and Asp^196^, fully conserved in PRs (Fig. 1).

**Fig. 2.**
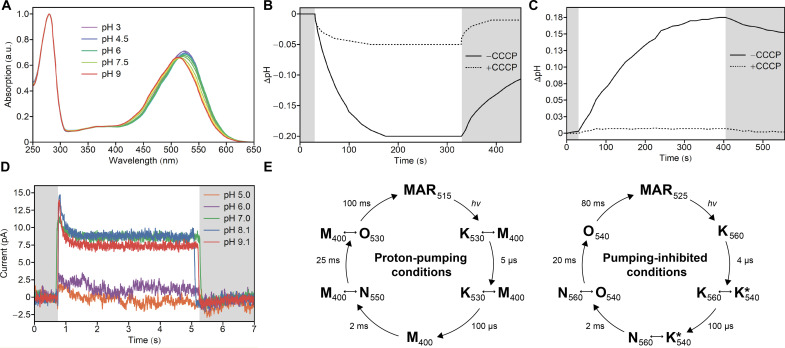
Functional characterization of MAR. (**A**) Absorption spectra of solubilized MAR from pH 5 to 9 with steps of 1.5. Spectra are normalized for convenience. a.u., arbitrary unit. (**B**) Change in solution pH values upon illumination of MAR-expressing *E. coli* cells. The addition of carbonyl cyanide *m*-chlorophenyl hydrazone (CCCP) strongly reduces the illumination-induced change in pH. (**C**) Change in solution pH values upon illumination of soybean phospholipid liposomes containing MAR. The addition of carbonyl cyanide *m*-chlorophenyl hydrazone eliminates the illumination-induced change in pH. (**D**) Photocurrents induced by illumination of MAR-containing liposomes adsorbed to a planar BLM in a buffer containing 10 mM MES and 10 mM NaCl at different pH values. (**E**) Scheme of MAR photocycle under proton-pumping (pH 10) and pumping-inhibited (pH 5) conditions. Small double-headed arrows between two states indicate a slow equilibrium between these states.

Similar to other PRs, MAR exhibited pH-dependent absorption spectra, with the absorption maxima at 512 and 525 nm when measured at pH 9 and 5, respectively (Fig. 2A). The calculated p*K*_a_ (where *K*_a_ is the acid dissociation constant) of the proton acceptor pair His^51^-Asp^72^ is ~7.4 (fig. S2A). pH measurements of the MAR-containing *Escherichia coli* cells and proteoliposome suspensions show that MAR is a light-driven outward proton pump (Fig. 2, B and C). The measurement of MAR-induced photocurrent on the planar bilayer lipid membrane (BLM) shows limited proton-pumping activity at low pH and increased activity at neutral and high pH (Fig. 2D).

The photocycle of MAR was measured by flash photolysis in the temperature range from 0° to 50°C in 10°C steps and at pH 5, 7.5, and 10 (figs. S2 to S5), as described in Materials and Methods. To better mimic the natural membrane environment and, as a consequence, avoid protein aggregation at high temperatures, we reconstructed the protein into nanodiscs. Measuring photocycles at different temperatures allowed us to obtain thermodynamic parameters of the photocycle (table S1), which could be used for better planning of future time-resolved and cryotrapping crystallographic experiments. The photocycle can be well described by irreversible first-order reactions between pure intermediate states and states comprising fast equilibria of two subsequent pure states (fig. S6). The photocycles are schematically depicted in Fig. 2E. At high pH, where MAR functions as an outward proton pump, two states with fast equilibria decay to form an M intermediate state in the microsecond range. The K intermediate is red shifted compared to the ground state, whereas the M intermediate is notably blue shifted, characteristic of the deprotonated form of the RSB. Two spectrally distinct late red-shift intermediates, N and O, follow before the protein relaxes back to the ground state. The photocycle at low pH, where no pumping is observed, differs substantially from that measured at high pH. It lacks the blue-shifted M intermediate state. Instead, a red-shifted intermediate called K* is observed. Last, the photocycle at neutral pH is a mixture of the two modes described above. Overall, the photocycle of MAR and its dependence on pH is similar to that described for other PRs, particularly green PR [GPR (*25*)]. It is important to note that although the measurements were performed in nanodiscs, a lipid-like environment, the complex problem of the influence of the native lipid environment and protein oligomerization on the protein photocycle is beyond the scope of this work and requires further studies [see refs. (*26*–*30*) and review (*21*)]. In summary, the sequence alignment and the photocycle measurements confirm that MAR is a member of the PR family, which functions as an outward proton pump at neutral and high pH.

### Crystal structures of MAR at atomic and true-atomic resolutions

To get insights into the molecular basis of PR function, we crystallized MAR using the in meso approach (*31*, *32*). Several types of twinning-free crystals were obtained, namely orange form and rose form (figs. S7 and S8). They relate to different functional states of MAR, as described further here. The crystals are of high quality, providing atomic (M-like and O* states) and true-atomic-resolution (ground and O states) structures of MAR at a resolution of up to 1.09 Å (in the case of the O state structure). Such data allowed us to analyze the key functional steps in PR function at the atomic level in the same manner as we reported for *Hs*BR (*3*). Data collection and structure refinement statistics are presented in tables S2 and S3, respectively. Examples of the corresponding electron densities are shown in figs. S9 to S12.

**Fig. 3.**
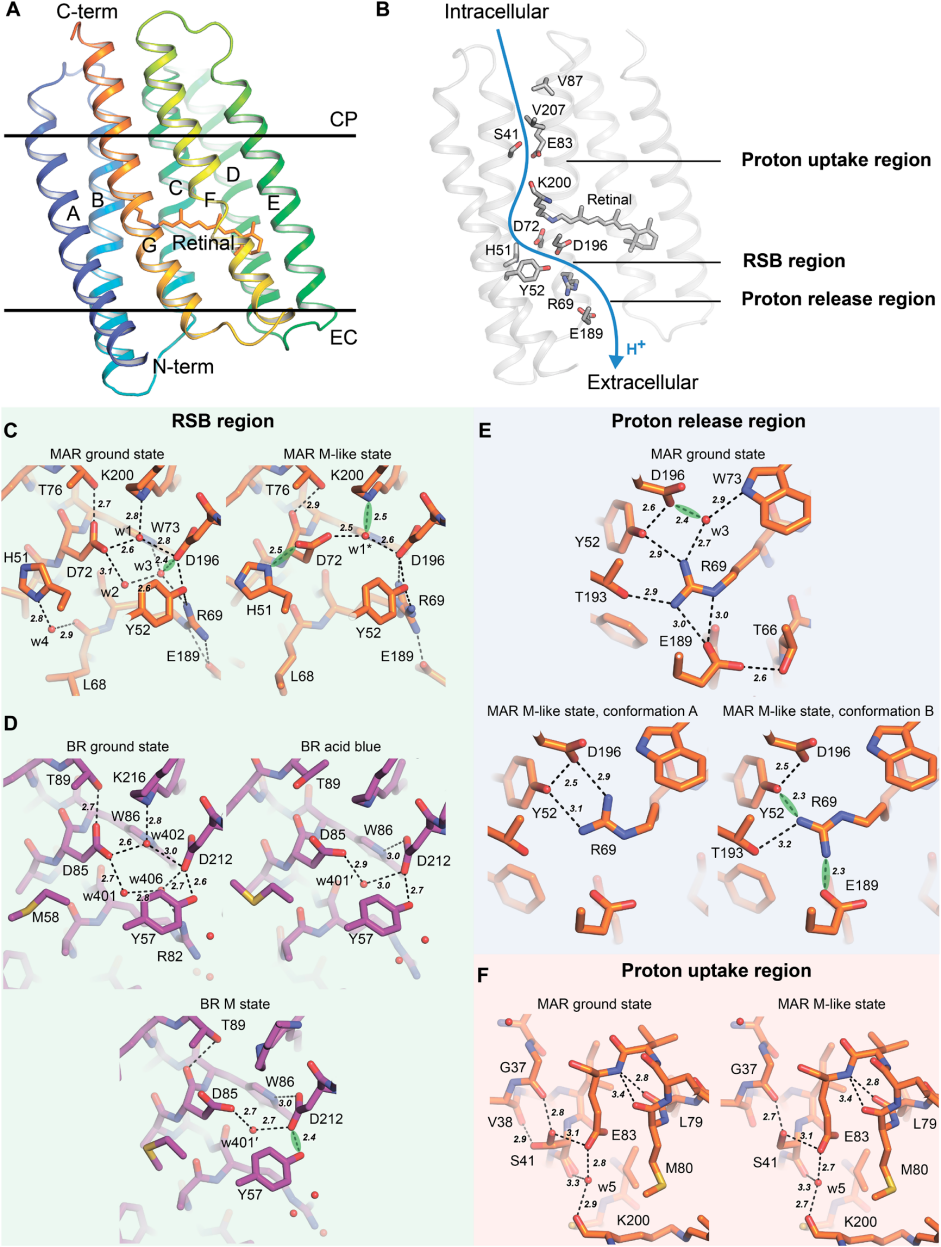
Structures of the ground and M-like states of the MAR photocycle. (**A**) Overall structure of MAR. Hydrophobic membrane boundaries were calculated using the PPM server (*116*). (**B**) Proton translocation pathway in MAR. Key residues are shown as sticks. (**C** and **D**) Comparison between RSB regions in MAR and *Hs*BR, respectively. For MAR, structures at high pH (ground state) and low pH (M-like state) were taken. For *Hs*BR, the structures of the ground state [PDB ID: 7Z0A (*3*)], acid blue *Hs*BR [PDB ID: 1X0I (*40*)], and the M state [PDB ID: 7Z0E (*3*)] were taken. It is worth discussing the difference between the Thr^89^ conformation in the acid blue *Hs*BR structure and the ground and M states. Our analysis indicates that this may be an error because, in such an orientation, threonine does not form H-bonds with its environment. Thus, no differences, besides retinal isomerization, were identified between acid blue *Hs*BR and the M state *Hs*BR. (**E**) Structures of the proton release region in MAR in the ground and M-like states. (**F**) Structures of the proton uptake region in MAR in the ground and M-like states. [(C) to (F)] Residues are depicted with sticks. Polar contacts are shown with black dashes. SHBs are shown with green clouds. SHBs have been assigned as described in Materials and Methods.

Overall, MAR consists of seven transmembrane helices, A to G, connected through short intracellular and extracellular loops (Fig. 3A). A retinal chromophore is covalently bound to Lys^200^ of helix G. The fold of the MAR is similar to that of other PRs (*7*, *27*, *29*, *33*–*36*). Helical C_α_ RMSD (i.e., root mean square deviation of C_α_ atom positions, calculated over residues belonging to α helices) between the MAR backbone atoms’ positions in the ground state and those of blue PR [BPR (*34*)] is ~1.3 Å (fig. S13). Given the similarity of MAR with other PRs, the putative proton translocation pathway comprises the proton donor Glu^83^, the RSB pocket with the conserved His^51^-Asp^72^ pair, and the proton release region (Asp^196^, Tyr^52^, Arg^69^, and Glu^189^), which separates the RSB pocket from the extracellular bulk (Fig. 3B). Positions of these amino acid residues are generally similar to those observed in the structures of other PRs (*7*, *27*, *29*, *33*–*36*).

### The orange-form crystals reveal the ground state of MAR under proton-pumping conditions

**Fig. 4.**
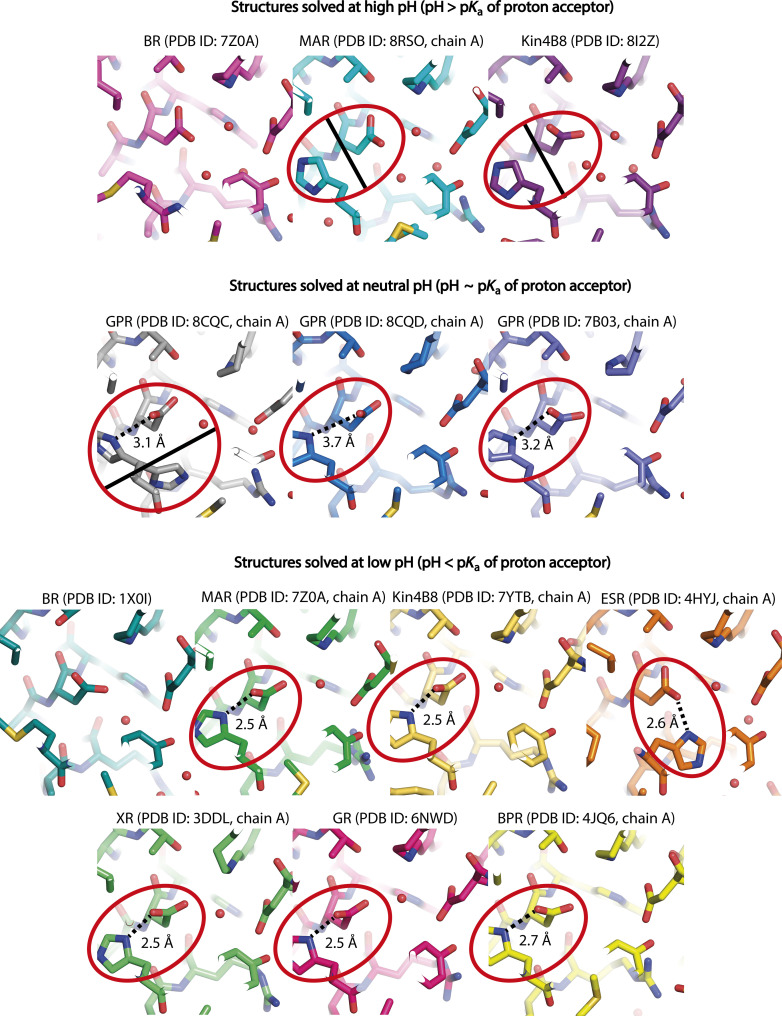
Comparison of the RSB region in the structures of PRs and *Hs*BR solved at different pH values. The His-Asp pair, fully conserved in PRs, is highlighted. H-bonds between His and Asp are shown with a dashed line. The absence of H-bonds is shown with a solid line.

First, we obtained a 1.25-Å-resolution structure of MAR in the ground state at pH 8.8 using orange-form crystals of MAR (absorption maximum at 515 nm). This is the first atomic-resolution structure of a PR under proton-pumping conditions, as the pH of the structure roughly corresponds to the average pH of the ocean photic zone of 8.2 (*37*). Unexpectedly, the RSB region of MAR in the ground state resembles that of *Hs*BR (Fig. 3, C and D). Namely, Asp^72^, Asp^196^, and three water molecules, referred to as w1, w2, and w3 (analogous to w402, w401, and w406 in *Hs*BR, respectively), form a pentagon of H-bonds. Notably, His^51^ does not interact with Asp^72^. Instead, His^51^ is bonded to the carboxyl oxygen of the Leu^68^ main chain via the w4 water molecule. This contrasts with most previously published PR structures solved under acidic or subacid conditions where pumping was inhibited (pumping-inhibited conditions). Figure 4 illustrates the differences in the conformations of the RSB region in PRs and *Hs*BR at different pH values. In most structures, the His-Asp pair was H bonded. The corresponding Protein Data Bank accession numbers (PDB IDs) are 4JQ6 [BPR (*34*)], 7B03 and 8CQD [GPR (*27*, *28*)], 4HYJ [*Exiguobacterium sibiricum* rhodopsin, ESR (*7*)], 3DDL [xanthorodopsin, XR (*33*)], 6NWD [*Gloeobacter* rhodopsin, GR (*29*)], and 7YTB [Kin4B8 (*35*)]. An exception is a 2.3-Å-resolution cryo–electron microscopy structure of Kin4B8 [PDB ID: 8I2Z (*35*)]. Its active site configuration under proton-pumping conditions is similar to that of MAR. In the cryo–electron microscopy structure of GPR solved at a 2.8-Å resolution [PDB ID: 8CQC (*28*)], the RSB conformation reminds a superposition of both proton-pumping and pumping-inhibited states, which is expected for the structure solved at intermediate pH.

At the extracellular side of the ground state of MAR, Arg^69^ is bonded to Tyr^52^ and Glu^189^ (Fig. 3E), resulting in a chain of H-bonds (HBCs; Lys^200^-w1-Asp^196^-Tyr^52^-Arg^69^-Glu^189^) propagating toward a large water-filled cavity, leading further to the extracellular bulk. At the cytoplasmic side, the proton donor Glu^83^ is oriented toward the RSB and is bonded to the carbonyl group of Lys^200^ through a single water molecule w5 (Fig. 3F), unlike Asp^96^ in *Hs*BR, which in the ground state is completely isolated from RSB in the hydrophobic cavity. Glu^83^ in MAR is separated from the cytoplasmic bulk by hydrophobic residues Val^87^ and Val^207^ (Fig. 3B). Ser^41^, adjacent to Glu^83^, exists in two conformations: one bonded to Glu^83^ and the carbonyl oxygen of Gly^37^, with the other bonded only to the carbonyl group of Val^38^ (Fig. 3F).

Overall, while MAR is remarkably different from archaeal and eukaryotic proton pumps at the extracellular side and near the proton uptake region, it shares a similar pentagonal organization of the H-bonds in the active site, pointing toward the latter’s indispensability for transferring a proton from RSB to the proton acceptor upon the formation of the M state. This active site conformation is observed only for PRs under proton-pumping, high pH conditions.

### The His-Asp pair is the primary proton acceptor in PRs, as revealed by the M-like state of MAR

The His-Asp pair is fully conserved in PRs (Fig. 1). It has been shown that both residues are involved in accepting a proton from RSB upon the formation of the M state (*8*, *9*). The presence of the His residue in the RSB pocket increases the p*K*_a_ of the proton acceptor Asp to around 7 (*8*), which is the possible reason why PRs, unlike *Hs*BR, are nonfunctional at low pH (*30*). Inhibition of proton pumping at low pH is also associated with the disappearance of the M state (*5*, *30*, *38*). Notably, PR functionality at low pH and the M state can be restored by mutating His to Met residue, recreating the RSB region of *Hs*BR (*9*, *39*). In the absence of high-resolution structures of PRs at different functional states, the mechanisms of proton transfer from RSB to the His-Asp pair, proton storage within the pair, proton release from the pair to the extracellular space, and inhibition of proton pumping at low pH remain elusive.

The orange-form crystals that were used to solve the MAR ground state structure under proton-pumping conditions also grew at pH 5.2, i.e., under pumping-inhibited conditions. These low-pH crystals were visually indistinguishable from the high-pH crystals and were also in the same symmetry group, but their spectrum was notably different (fig. S7A). We determined a 1.6-Å-resolution structure from such crystals, which provided a molecular basis for the inhibition of proton pumping in PRs under acidic conditions and the role of the His-Asp pair in it.

In the ground state of MAR solved under proton-pumping conditions, His^51^ and Asp^72^ are not interacting. In contrast, the structure of MAR under pumping-inhibited conditions shows that His^51^ and Asp^72^ reorient to form a 2.5-Å short H-bond (SHB; Fig. 3C). This reorientation of the residues and formation of an SHB indicate that this pair acquired a proton from the acidic solution. Protonation of the group is also substantiated by the red shift of the absorption spectrum maximum from 515 to 524 nm for the high- and low-pH crystals, respectively (fig. S7, D and E). Last, this is supported by the similarity of the RSB region in MAR at pH 5.2 with that of other PRs also solved under low pH conditions (Fig. 4).

Reorientation of Asp^72^ toward His^51^ in the low-pH MAR leads to dehydration of the RSB region in a similar way to that shown for *Hs*BR in the L and M states (*3*): Only one water molecule, w1*, remains in the region. Given the similarity between the structures of the M state and the acid blue (low pH) form of *Hs*BR (Fig. 3D) (*40*), the structure of MAR at low pH can be a reasonable model for the M state in the extracellular part of the pump. The acid blue *Hs*BR, low-pH MAR, and the M states of the corresponding proteins all have a protonated proton acceptor, explaining their similarity. Considering these rationales, we will refer from here on to the structure of the orange-form MAR at high pH as the ground state and that at low pH as the M-like state.

The observed structural rearrangements in the M-like state of MAR provide insight into the mechanism of proton transfer to the proton acceptor group and the proton-pumping inhibition at low pH. We suggest that with the formation of the M state, a proton is transferred from the RSB to the Asp^72^ carboxylate, leading to the formation of the His^51^-Asp^72^ SHB, where the proton is stored until the decay of the O state. The involvement of two residues in storing a proton through the SHB explains the increased p*K*_a_ as compared to *Hs*BR, in which Asp^85^ alone accepts a proton (*5*, *41*). In contrast, under acidic conditions, the already protonated His-Asp pair cannot accept a proton from RSB, preventing RSB deprotonation and leading to the absence of the M state and proton pumping.

Analysis of the proton release region reveals differences between MAR and *Hs*BR upon protonation of the proton acceptor. While Arg^82^ of *Hs*BR flips to the extracellular part in the acid blue and M states, Arg^69^ of MAR in the M-like state remains oriented toward RSB (Fig. 3E). It acquires some mobility, which is evident from the coexistence of two conformations. The flip of the corresponding arginine plays an important role in *Hs*BR during the proton release to the extracellular space by a proton-release group (*3*) that is absent in PRs.

To summarize, upon the formation of the M state in MAR, the RSB donates its proton to the proton-accepting pair His^51^-Asp^72^, leading to dehydration of the region and the disruption of the pentagonal organization of the H-bonds observed for the ground state. Such changes are similar to those observed for the M state of *Hs*BR (*3*), with minor nuances in the extracellular part associated with protein differences.

### MAR functionality is limited in orange-form crystals by tight crystal contacts that can be overcome by spacers

**Fig. 5.**
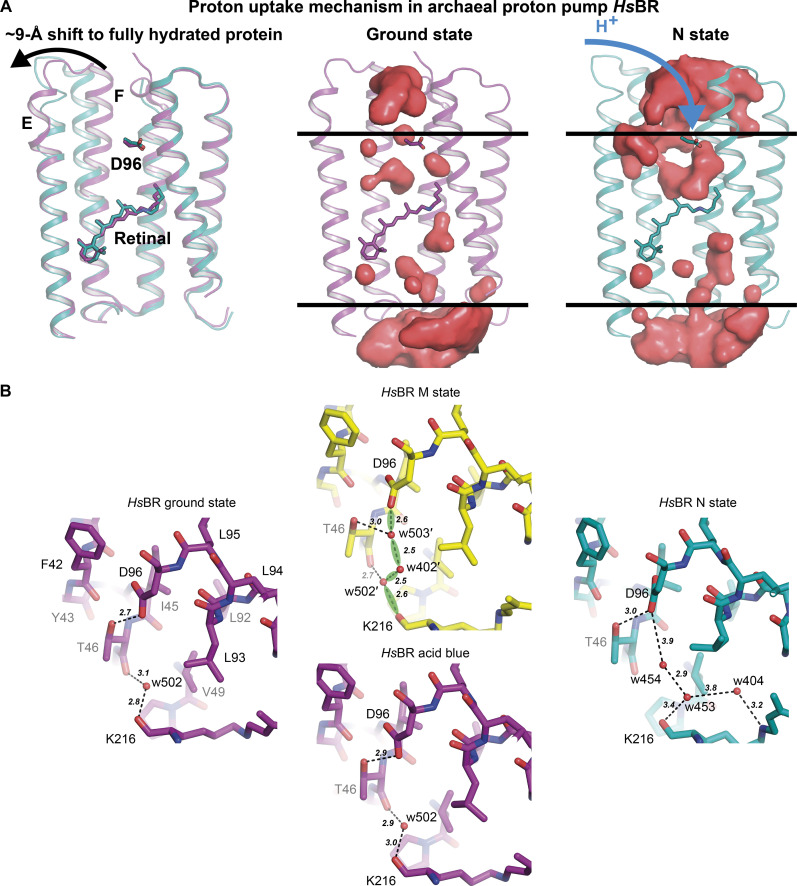
Proton uptake mechanism in *Hs*BR. (**A**) Opening of *Hs*BR to the cytoplasm. Structures in the ground state [PDB ID: 7Z0A (*3*); colored purple] and N state [PDB ID: 6RPH (*15*); colored teal] were taken for the illustration. Proton acceptor residues and retinal-lysine molecules in *Hs*BR are depicted as sticks. Cavities were calculated using HOLLOW (*115*). (**B**) Comparison of the proton uptake site in the structures of *Hs*BR in the ground state [PDB ID: 7Z0A (*3*); colored purple], in the M state [PDB ID: 7Z0E (*3*); colored yellow], in the acid blue form [PDB ID: 1X0I (*40*); colored purple], and in the N state [PDB ID: 6RPH (*15*); colored teal].

**Fig. 6.**
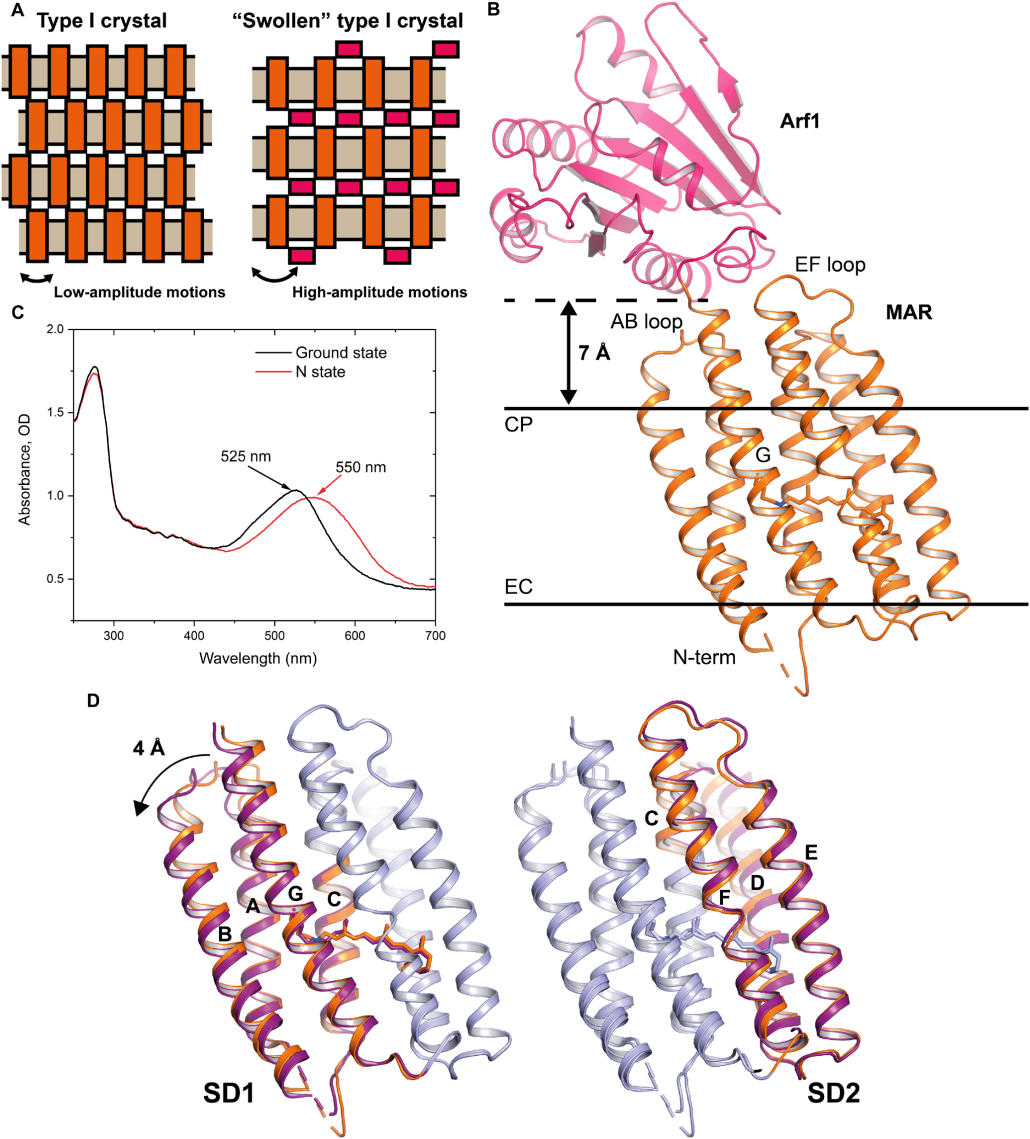
Utilization of a spacer domain for obtaining the structure of the late intermediate of MAR. (**A**) Utilization of a spacer for “swelling” the crystal. (**B**) Overall structure of the MAR-Arf1_L8K,Q71L_ chimera. MAR is colored orange, while Arf1 is colored pink. The predicted hydrophobic-hydrophilic borders are shown as black bars. (**C**) Results of the cryotrapping experiment with the crystals of the MAR-Arf1_L8K,Q71L_ chimera. The spectrum before illumination with the 532-nm laser is colored black. The spectrum after illumination with the 532-nm laser at room temperature for 2 s is colored red. (**D**) Comparison between the structures of the MAR-Arf1_L8K,Q71L_ chimera before and after the illumination, resulting in the accumulation of the N state. Structures are shown as cartoons and are aligned in two possible ways. The first pair of structures is aligned using SD2 residues (while SD1 residues are colored orange and purple for the ground and N states, respectively). The second pair of structures is aligned using SD1 residues (while SD2 residues are colored orange and purple for the ground and N states, respectively).

In MRhs, the ion-transporting cycle is accompanied by the accessibility switch between the extracellular and cytoplasmic sides of the membrane to allow efficient ion release and uptake but also to prevent the backflow of the ions (*42*). The switch corresponds typically to large-scale conformational changes, as demonstrated, for instance, for the N state of *Hs*BR (Fig. 5A) (*15*). Unfortunately, such large-scale rearrangements are often constrained/modified by the tight membrane sandwich packing in type I crystals of MRhs grown in meso (Fig. 6A). This is also true for the tightly packed MAR crystals (Supplementary Text 2).

When comparing the changes that occur in the M state of *Hs*BR and the M-like state of MAR in their cytoplasmic parts, we see no notable rearrangements for the latter (Figs. 3F and 5B). For both transporters, RSB reprotonation occurs during the M-to-N state transition involving water molecules in the cytoplasmic part. In the ground state of *Hs*BR, RSB is isolated from the proton donor Asp^96^ in the hydrophobic pocket, and extensive hydration of the region is required for its reprotonation (Fig. 5B) (*3*). Two additional water molecules, w402′ and w503′, that interconnect Asp^96^ with the carbonyl group of the retinal-carrier Lys^216^ (Fig. 5B) are found in the region in the M state. These water molecules bind Asp^96^ to RSB upon the M-to-N state transition, where the reprotonation of the latter occurs (*15*).

On the other hand, in the ground state of MAR, the proton donor Glu^83^ is already H bonded with the carbonyl group of Lys^200^ via a water molecule w5 (Fig. 3F). Therefore, there is no need for such substantial hydration of the region for reprotonation, which could explain why we do not see any differences between the M-like and ground states of MAR in the cytoplasmic part. Another explanation could be that the M-like structure of MAR contains an all-*trans* retinal, while 13-cis,15-anti isomerization is expected for the M state in PRs (*43*–*46*). As can be seen from the comparison of the structures of the M and acid blue states of *Hs*BR, hydration of the cytoplasmic part does not occur for the latter (Fig. 5A). Isomerization of retinal in the M state of *Hs*BR results in the movement of the carbonyl group of Lys^216^, which creates an additional space for water molecules in the cytoplasmic part. Thus, the structure of the M state would be beneficial in understanding the nuances of the RSB reprotonation in MAR.

However, the notable difference between the photocycles in crystals and in nanodiscs (Supplementary Text 2) suggests that tight crystal contacts might prevent the hydration of MAR in the cytoplasmic part. The problem can be addressed by adding a spacer, a water-soluble protein fused to the membrane protein to increase the distance to the nearest neighbor and allow for large-amplitude motions (Fig. 6A). With this in mind, we designed and used a MAR-Arf1_L8K,Q71L_ chimera (Fig. 6B). Arf1_L8K,Q71L_ is a mutant of a small guanosine triphosphatase Arf1. The chimera was crystallized, and the crystals diffracted to 2.3 Å. The conformation of the MAR molecule is similar to that in the ground state of MAR alone (helical C_α_ RMSD is 0.4 Å). Arf1_L8K,Q71L_ is located at least 7 Å from the predicted hydrophobic-hydrophilic boundary of a crystalline layer and forms only minor contacts with the AB and EF loops of MAR and the cytoplasmic part of helix G (Fig. 6B). This suggests that the MAR-Arf1_L8K,Q71L_ chimera could be used to study late photocycle intermediates of MAR that are inhibited by crystal contacts in the orange-form crystals.

### The opening of MAR to the cytoplasmic part is initiated upon the RSB reprotonation in the N state

To obtain the N state, we continuously illuminated the crystals of MAR-Arf1_L8K,Q71L_ with a 532-nm laser at room temperature and then flash frozen them in a 100 K cryostream. Microspectrophotometry of the crystal before and after the cryotrapping indicated the accumulation of the red-shifted intermediate (λ = 550 nm) corresponding to the N state of MAR (Fig. 6C). Using the crystals with the cryotrapped intermediate, we obtained the structure of the N state at the 2.3-Å resolution.

**Fig. 7.**
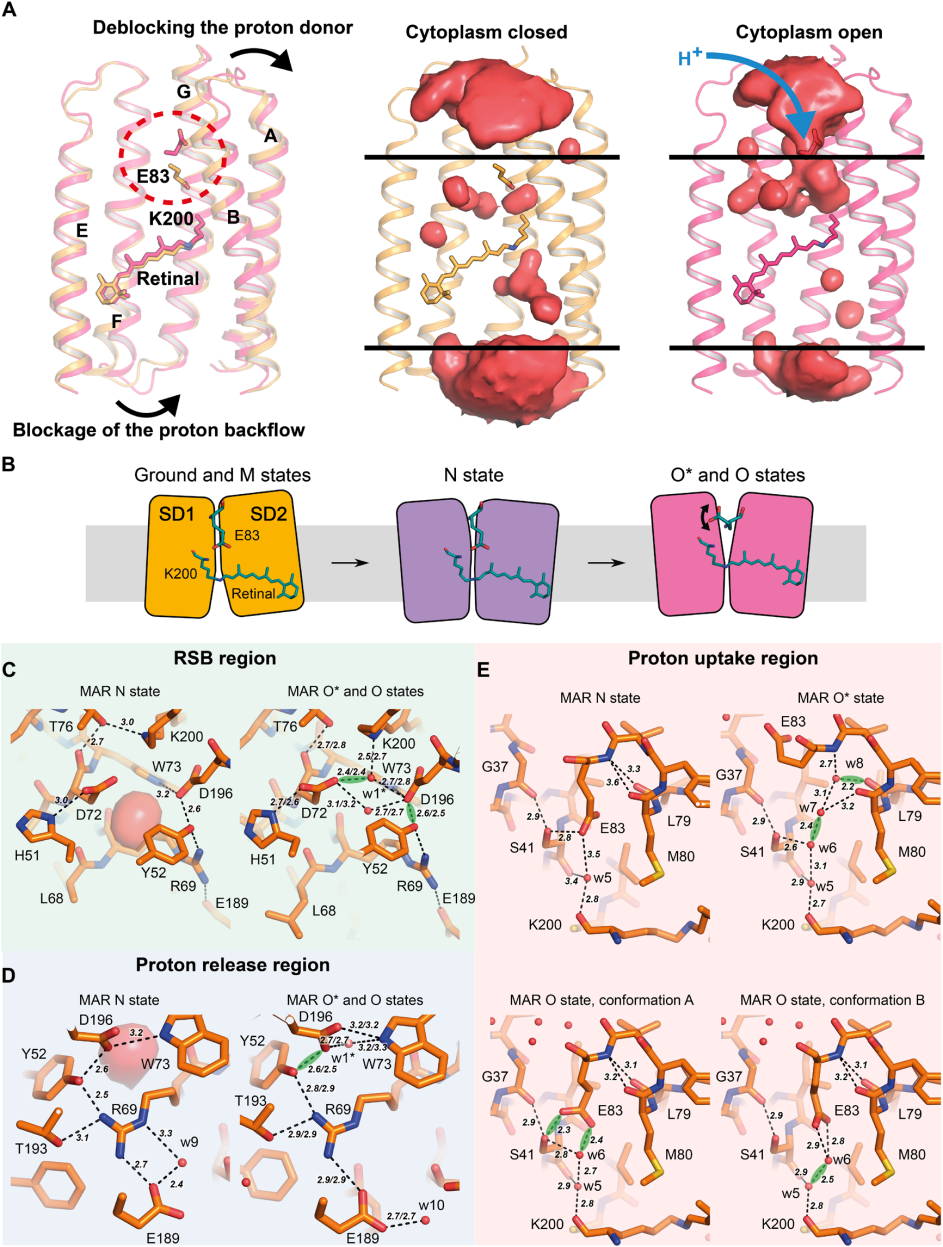
Structures of the late intermediates of the MAR photocycle. (**A**) Structures of MAR in the cytoplasm closed (ground state, colored orange) and cytoplasm open (O* state, colored rose) states. Proton acceptor residues and retinal-lysine molecules are depicted as sticks. Cavities were calculated using HOLLOW (*115*). (**B**) Schematic representation of SD1/SD2–dependent alternating access of RSB to cytoplasmic and extracellular bulk solvents occurring over the MAR photocycle. (**C** to **E**) Structures of the RSB, proton release, and proton uptake regions, respectively, in the N, O*, and O states of MAR. Residues are depicted with sticks. Polar contacts are shown with black dashes. [(C) and (D)] H-bond lengths are given correspondingly for O*/O states. SHBs are shown with green clouds. SHBs have been assigned as described in Materials and Methods.

The ground and N state structures of MAR in the chimera do not differ considerably, with the helical C_α_ RMSD of 0.6 Å. However, some notable changes exist, especially in the AB loop, where the Cα-Cα distance reaches 4 Å. The structural differences between the states can be described best as motions of two rigid subdomains (SD1 and SD2) relative to each other. Here, SD1 comprises residues Met^1^-Phe^81^ (helices A and B and extracellular part of helix C) and Asp^183^-Glu^217^ (helix G). SD2 consists of residues Val^82^-Gln^182^ (cytoplasmic part of helix C; helices D to F). When aligned by SD1 and SD2, helical C_α_ RMSDs between both forms are 0.4 and 0.1 Å, respectively. This motion of SD1 and SD2 is even more prominent upon the formation of the O state, as it will be shown later, which lastly results in the accessibility switch to the cytoplasm (Fig. 7, A and B, and movie S1).

Electron density maps indicate that the retinal conformation in the N state represents the 13-cis,15-anti form, as expected from the spectroscopy data for other PRs (*41*). However, the resolution of the data does not allow us to build the retinal conformation using only structural data and to judge whether there are other sparsely populated isomers of retinal in the N state structure [which requires atomic or even true-atomic resolutions, like was previously shown for the ground states of archaeal proton-pump Archaerhodopsin-3 (*47*) and viral rhodopsin OLPVR1 (*48*)]. Similarly to *Hs*BR (*3*), the RSB is stabilized with the H-bond to the adjacent threonine Thr^76^ (Fig. 7C). The analysis of the internal cavities of MAR in the N state suggests that similar to the M-like state, one water molecule is found between Asp^72^ and Asp^196^; however, it is slightly shifted toward the extracellular side. His^51^ and Asp^72^ are H bonded with a bond length of 3.0 Å. Asp^196^-Tyr^52^-Arg^69^-Glu^189^ residues form a continuous proton wire from the RSB to the extracellular bulk (Fig. 7D). Last, the configuration of the proton donor region around Glu^83^ is identical to that found in the ground and M-like states (Fig. 7E), confirming that the accessibility switch has not yet been fully completed.

Thus, in the N state of MAR, the proton donor residue Glu^83^ reprotonates the deprotonated RSB, initiating the opening of the protein to the cytoplasmic part. Glu^83^, which is expected to be deprotonated at this point, remains H bonded to the carbonyl oxygen of Lys^200^, as it was in the ground and M-like states. This means that the residue is still isolated from the cytoplasmic bulk inside the protein, and further opening to the cytoplasm is required for reprotonation.

### The rose-form crystals of MAR contain the protein trapped in the O state by crystallization conditions

As mentioned above, we obtained several crystal forms of MAR during our crystallization trials. While the crystals of MAR in the ground state appeared orange (maximum absorption wavelength of 515 nm; fig. S7A), we also obtained rose-colored crystals under different crystallization conditions (fig. S7B). These crystals had an absorption maximum of 530 nm, which is expected for an O intermediate state of MAR. The structure of MAR determined using the rose-colored crystals demonstrated global structural rearrangements similar to those observed for the N state, however, at a notably larger amplitude (Fig. 7, A and B, and movie S1). Helical C_α_ RMSD between the orange and rose crystal forms is 1.2 Å, but when aligned only by SD1 or SD2, the values are 0.8 and 0.3 Å, respectively.

Because of the accessibility switch, proton donor Glu^83^, previously confined by helix B, becomes hydrated from the cytoplasmic bulk. At the same time, the extracellular part of MAR turns into an almost fully dehydrated region because of the FG-loop closure (a key role in this process is the formation of a hinge in helix F; see Supplementary Text 3 for details). It can be concluded that the structural and spectroscopic data of the rose form of MAR allow assigning this state to the late O intermediate. Apparently, the O state is trapped by the crystallization conditions in the rose-colored crystals.

Trapping of membrane proteins in different functional states by crystallization conditions has been reported before (*49*, *50*). However, trapping the photocycle intermediates of MRhs without excitation by light is complicated because some of them are characterized by photoisomerized retinal in a 13-cis,15-anti configuration. MRh intermediates having an all-*trans* retinal (e.g., like that in the ground state), on the other hand, can be trapped, as has been previously shown for the O state of the *Sy*HR anion pump (*10*). Fortunately, the O intermediate state of PRs harbors a retinylidene Schiff base with an all-*trans* retinal (*51*), allowing it to be trapped in the dark under certain crystallization conditions.

### Structures of the O state of MAR, solved at different pH values, reveal the proton uptake mechanism

Using the rose-form crystals, we obtained two different structures of the O state: under the proton-pumping (pH 8.4) and pumping-inhibited (pH 4.6) conditions at the 1.4- and 1.09-Å resolutions, respectively. We will refer to these structures as the O* and O states for clarity. These structures demonstrate no differences in the RSB and proton release regions and are similar to the N state structure in the corresponding regions (Fig. 7, C and D). However, they have remarkable differences in the proton uptake region (Fig. 7E). Compared to the structures of the ground, M-like, and N states, in the O state structure, an additional water molecule w6 is incorporated in the HBC between Glu^83^ and the carbonyl group of Lys^200^. Glu^83^ and w6 acquire two conformations. In the first conformation, Glu^83^ makes an H-bond with Ser^41^ and w6. w6 is stabilized by Ser^41^ and w5. In the second conformation, both Glu^83^ and w6 break their bonds with Ser^41^. Differently, in the O* state, Glu^83^ is flipped to the cytoplasmic bulk. Two additional water molecules (w7 and w8) are wedged into the α-helical turn between Met^80^, Leu^79^, and Glu^83^, forming a continuous HBC connecting the backbones of Lys^200^ and Glu^83^. Ser^41^ stabilizes this chain.

We hypothesized that the structures of the O* and O states obtained with the spectrally indistinguishable rose-form crystals at different pH values could have a functional meaning in the MAR photocycle. Two events should happen upon the transition of the N state to the O state in PRs (*52*). First, the retinal returns to the all-trans isomerization. Second, the proton acceptor reprotonates from the cytoplasmic bulk. Because of a lack of structural data, these steps are generally assumed to happen simultaneously. However, given our structures at different pH values, we suggest that they may happen sequentially. First, upon forming the O* state intermediate, the retinal returns to the all-trans isomerization. A deprotonated, negatively charged Glu^83^ is flipped to the cytoplasm, seeking the proton. Next, when Glu^83^ captures the proton from the bulk, it becomes neutral and flips back toward the retinal, forming the O state.

To confirm our hypothesis directly, we performed cryotrapping of the O* state crystals using a protocol similar to that described earlier to obtain the N state structure. The isomorphous difference map clearly shows the flip of Glu^83^ in the direction of the retinal (tables S4 and S5 and fig. S14). Such a flip is possible only after the accessibility switch reaches its maximum amplitude upon forming the O* state intermediate. In both ground and M-like structures and even in the N state, where the accessibility switch has not fully evolved, Glu^83^ cannot flip to the cytoplasm as it is locked inside the protein (Fig. 7A, red dashed line). The N-to-O* transition unlocks the caged residue, allowing it to move back and forth between the cytoplasm and the inner cavity. The reprotonation of the proton donor Glu^83^ proceeds during the spectrally indistinguishable O*-to-O state transition [because of the remoteness of the proton donor from the RSB, see ref. (*53*)] and involves the raking-like movement of the residue.

The proposed mechanism of the proton donor reprotonation in bacterial outward proton pumps is principally different from that demonstrated for the archaeal outward proton pump *Hs*BR (*15*). Namely, it was shown that in *Hs*BR, the cytoplasmic halves of helices E and F, together with the EF loop, move by 9 Å, thus exposing the cytoplasmic internal part of MRh to the cytoplasm, allowing the direct reprotonation of Asp^96^ (Fig. 5A). In MAR, there is also an opening of the cytoplasmic side for the proton uptake; however, in this case, helices A, B, and G are all shifted with smaller amplitudes of only about 4 Å at maximum. One of the possible explanations is that for the reprotonation of Asp^96^ in *Hs*BR, complete hydration of the entire cytoplasmic channel and the RSB region could be necessary. For MAR, the accessibility switch toward the cytoplasmic side is sufficient to provide Glu^83^ with the necessary freedom to flip outside the protein for proton uptake. This hypothesis is in full agreement with recent molecular dynamic simulations (*54*) and investigations of activation volumes in *Hs*BR and GPR (*55*). It should be noted that the difference between the amplitudes of changes might not be that severe (*42*).

### Proton release mechanism of MAR

The structures of the O* and O states reveal one water molecule, w1*, in the RSB region, which displays two alternative conformations. In the first one, similar to the M-like state, the water molecule interacts directly with the RSB. In the second conformation, it is shifted toward the extracellular side like that predicted for the N state. Asp^196^ forms polar contacts with Tyr^52^, Trp^73^, and both w1* conformations. Arg^69^ adopts a single conformation and is H bonded to only Tyr^52^ but not to w1* or Asp^196^. Moreover, Arg^69^ is bonded to Glu^183^, developing an uninterrupted and single HBC from the His^51^-Asp^72^ pair to the extracellular bulk. This HBC is used for proton transfer from the pair to the extracellular space on the last step of the photocycle (*56*, *57*). However, we admit that small shifts of elements in this HBC, such as the flip of Asp^72^ to donate a proton to water w1*, are possible upon relaxation to the ground state (see Supplementary Text 3).

Unexpectedly, the His-Asp pair remains protonated in both O* and O state structures, as indicated by the H-bond (2.7 and 2.6 Å, respectively) between the residues. This observation contrasts with that of the ground state structure, in which the pair is sensitive to pH. This bond becomes a regular H-bond, instead of SHB, during the later steps of the photocycle. We suggest that the proton accessibility switch that happens upon the M-to-N-to-O* state transition increases the p*K*_a_ of the His-Asp pair by isolating the pair from the solvent. The accessibility switch allows it to stay protonated until the very end of the photocycle, even without requiring SHB. The difference in bond length on different photocycle stages could explain the controversy between the structural and computational data on the nature of this bond (*7*, *58*).

The proton release to the extracellular bulk takes place upon the O-to-ground state transition when the accessibility switch proceeds for the second time, returning the pair’s p*K*_a_ to the initial values. The accessibility switch back from the cytoplasmic to extracellular side is required and is the driving force for the proton release in MAR and other PRs. We should note here that if the His-Asp pair retained the SHB in this case, the switch itself would not result in the proton release from the pair.

To summarize, three major events proceed upon the rise and decay of the O state: First, Glu^83^ uptakes a proton from the cytoplasm and flips back inside MAR toward the RSB; second, an accessibility switch back from the cytoplasmic to extracellular side occurs; and third, the proton is released from the His^51^-Asp^72^ pair to the extracellular space. These events lead to the proton translocation from the cytoplasm to the extracellular space and the end of the MAR photocycle, returning the protein to the relaxed ground state.

### Mechanism of proton pumping by PRs

**Fig. 8.**
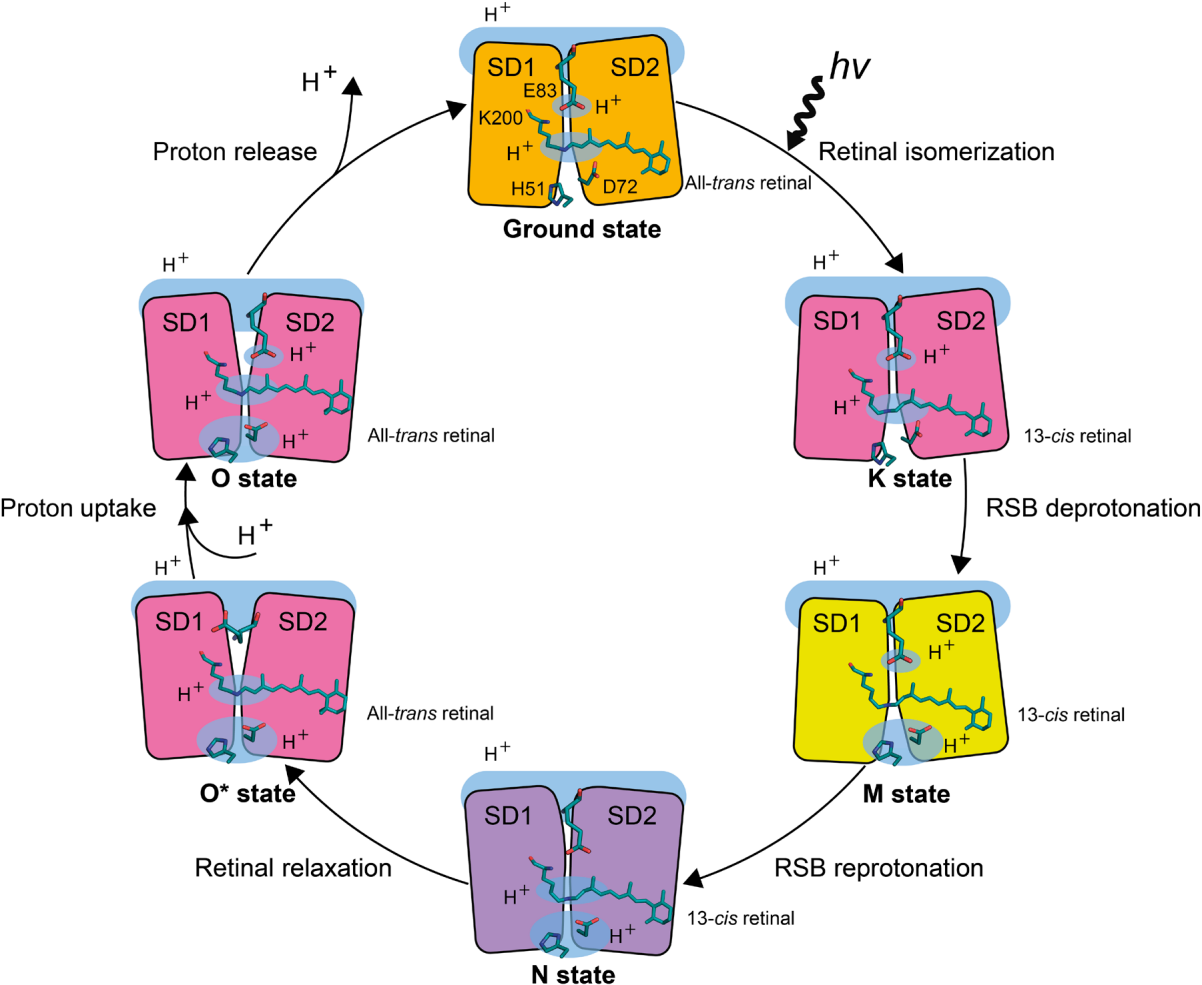
Schematic representation of the MAR photocycle. Proton donor (Glu^83^), RSB (Lys^200^ and retinal), and proton acceptor pair (His^51^-Asp^72^) are shown with sticks. Hydrogen positions are highlighted with blue clouds. SD1/SD2 subdomains of MAR, which are responsible for the accessibility switch, are demonstrated with rectangles and are colored according to the protein absorption spectrum in a particular intermediate state.

Structural and functional data presented here allow us to propose a mechanism for proton transport by PRs, taking MAR as an example (Fig. 8). First, in the ground state, the RSB is protonated, and the proton acceptor pair, His^51^-Asp^72^, is deprotonated, as indicated by the absence of the H-bond between the two residues. The RSB region of MAR in the ground state resembles that of *Hs*BR, having an all-*trans* retinal, two aspartate amino acid residues (Asp^72^ and Asp^196^), and three water molecules. The pentagonal organization of the H-bonds in the region is preserved between these very different proton pumps. Photon absorption by the retinal leads to its isomerization from the all-trans to 13-cis,15-anti isoform, resulting in the formation of the K state intermediate. With the formation of the M state, the proton is transferred from the RSB to the His^51^-Asp^72^ pair that forms an SHB. It is stored in this H-bond until nearly the end of the photocycle. Next, upon forming the N state, Glu^83^ donates its proton to the RSB, and the proton accessibility switch opens the protein to the cytoplasmic side (movie S1). This transition allows deprotonated Glu^83^ to be reprotonated from the cytoplasm at the next stage of the photocycle. Simultaneously, the high p*K*_a_ of the His-Asp pair is further increased, which does not allow the stored proton to leave the protein ahead of time and restricts proton backflow. The subsequent step consists of two spectrally indistinguishable states, which we refer to as O* and O. Upon the N-to-O* transition, the retinal returns to the all-trans isoform. Next, between the O* and O states, Glu^83^ is reprotonated from the cytoplasmic bulk. Last, upon relaxation to the ground state, proton release to the extracellular bulk occurs only when the proton accessibility is switched back from the cytoplasmic to extracellular side because of the return of p*K*_a_ of the His-Asp pair to the ground state values. The proton release pathway is constituted by conserved Asp^196^, Tyr^52^, Arg^69^, and Glu^189^ residues, forming a continuous HBC. It extends from the His-Asp pair to the extracellular space. The proton release might require only minor rearrangement of the wire, similar to that shown for the extracellular side of *Hs*BR in the M state (*3*).

The proposed mechanism is mainly based on the structural data on MAR and literature data on other PRs. Undoubtedly, applying various advanced techniques to MAR, such as Fourier transform infrared spectroscopy (*41*, *59*–*62*), quantum mechanics/molecular mechanics (*63*, *64*), solid-state nuclear magnetic resonance (*65*), and time-resolved crystallography (*66*, *67*), will allow us to clarify the details of proton transfer in MAR in the future. In particular, the study of early MAR intermediates, namely the K and M states, is essential to understand better the mechanism of proton transfer from RSB to the proton acceptor His^51^-Asp^72^, as well as the mechanism of RSB reprotonation by the proton donor Glu^83^.

## DISCUSSION

### Strategies for obtaining the late intermediates of MRh photocycle

High-resolution crystal structures of the late intermediate states of rhodopsin proton pumps are essential to disclose the complete mechanisms of light-driven proton transfer through the membrane. However, obtaining such structures is still a great challenge. As an example, for the best-studied proton pump *Hs*BR, only the N state structure of the wild-type protein at 2.6 Å is available at the moment (*15*). Two major issues complicate the determination of such structures. First, large-scale conformational changes associated with the late intermediates are incompatible with the crystal contacts (*13*). Second, even when these conformational changes appear, they usually result in considerable deterioration of crystal diffraction quality (*68*). In the latter case, it is sometimes possible to overcome the problem by reducing the occupancy of the active state, which was successfully applied in determining the active state structure of the sensory rhodopsin II (*68*). However, other approaches are required if the active state does not form in crystals, like in the case of the O state of *Hs*BR (*13*) and the N and O states of MAR.

In the current work, we used two approaches to study the late intermediates of MRhs, which allowed us to obtain the structures of the N and O states of MAR, a representative of PRs. The first approach assumes that it might be possible to trap active forms of the protein by carefully selecting crystallization conditions. With this approach, we obtained the structure of the O intermediate state of MAR. The second approach involves the use of spacers, water-soluble proteins fused to the membrane proteins. The spacers lead to less tight packing of the membrane protein layers in the type I crystals, thus allowing for larger conformational changes. With this approach, the N state was obtained by using the double mutant of a small guanosine triphosphatase Arf1 as a spacer. While fusion partner proteins were used previously to promote the crystallization of membrane proteins (*69*), they were not chosen to allow for large-amplitude motions. We believe that such approaches may facilitate obtaining the high-resolution structures of late intermediate states and help to push forward the structural studies of membrane protein intermediates by x-ray crystallography.

### Hydrogen atom positions are required to reveal the quantum mechanical nature of proton transport

Visualizing hydrogen atoms would be a logical continuation of the study. The positions of hydrogens are essential to reveal the nature of H-bonds truly (*70*) and to see the proton transfer along the wires. To visualize the hydrogens, a resolution of less than 1 Å is required (*71*, *72*), currently unavailable for MRhs, except for one example (*73*). Alternatively, neutron diffraction can be used (*74*), but its application to membrane proteins grown in LCP is still elusive because of the necessity of large crystals. Both problems still represent a great challenge.

### General molecular mechanism of active proton transport and its universality

A large body of work was performed because *Hs*BR became the model protein to study the molecular mechanism of the first step of bioenergetics [see ref. (*12*) and references therein]. Fifty years of extensive research resulted in considerable progress, which, combined with the present data, allows us to perform a general analysis. We will compare our results on MAR with recent similar atomic-resolution data on an archaeal outward proton pump *Hs*BR (*3*) and a bacterial inward proton pump *Bc*XeR (*20*). Each of these works provides information on the ground and major functional states. The brilliant quality of the data allows accurate determination of the lengths of H-bonds, which is important for distinguishing between different types of H-bonds. Last, these proton pumps are very different, representing outward and inward proton pumps. While outward proton pumps provide an electrochemical transmembrane proton gradient, which drives adenosine 5′-triphosphate synthesis (*75*, *76*), the biological function of inward proton pumps remains a mystery (*77*, *78*). If universal principles of proton transfer do exist, they might be deduced by comparing the data obtained for MAR, *Hs*BR, and *Bc*XeR.

**Fig. 9.**
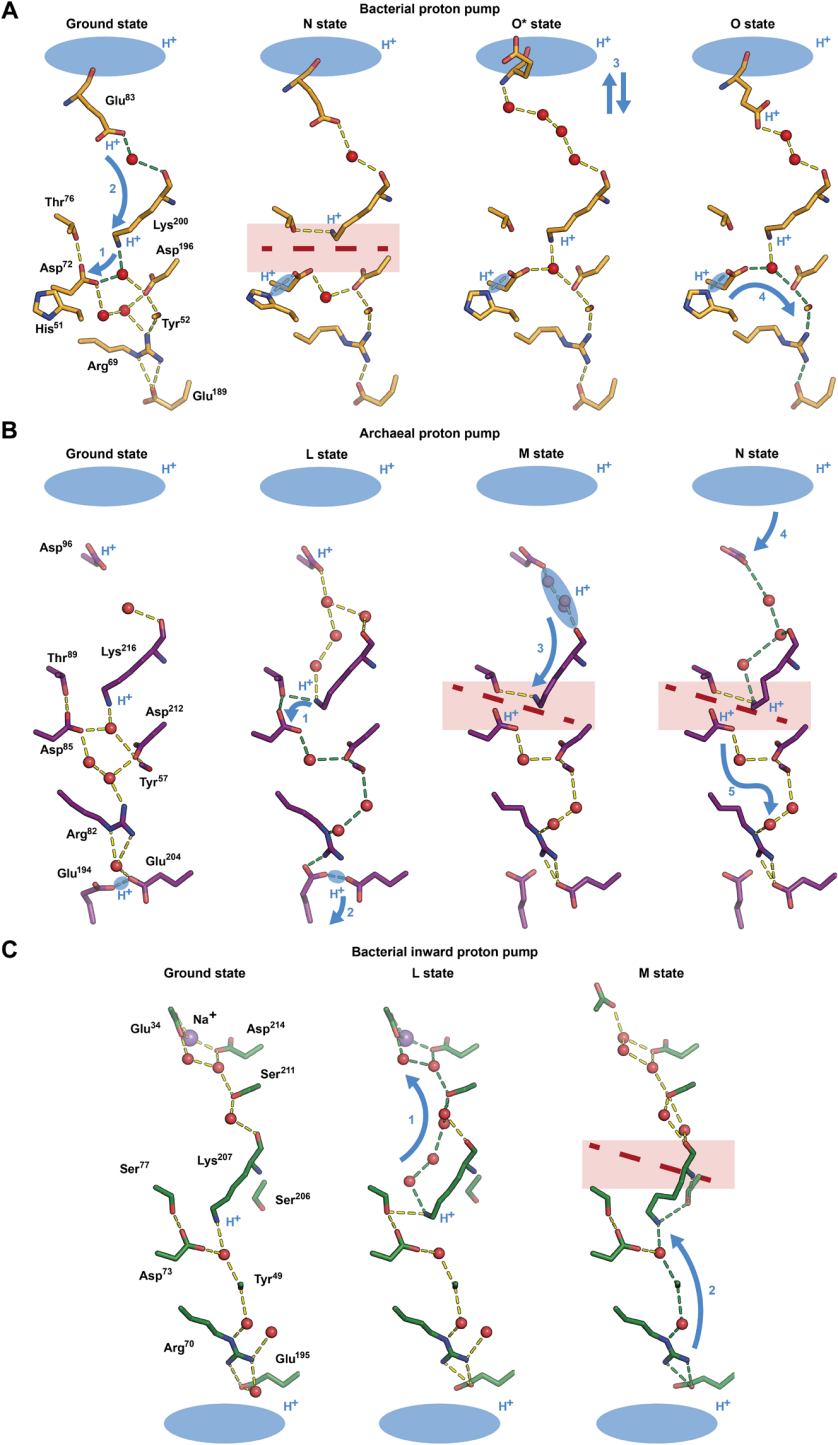
Universality of the mechanism of proton transfer in different proton transporters. (**A**) MAR, a representative of bacterial proton pumps. (**B**) *Hs*BR, a representative of archaeal proton pumps [PDB IDs: 7Z0A, 7Z0D, and 7Z0E (*3*) and 6RPH (*15*) for the ground, L, M, and N states, respectively]. (**C**) *Bc*XeR, a representative of bacterial inward proton pumps [PDB IDs: 7ZMY, 7ZN3, and 7ZN0 (*20*) for the ground, L, and M states, respectively]. Primary residues and water molecules involved in proton transportation are shown as sticks and spheres, respectively. HBCs are shown as dashed lines and are colored yellow. HBCs that are involved in the particular step of proton transportation are colored green. Interruptions of HBCs are shown with long red dashes and highlighted with red clouds. Proton transfer directions are shown with blue arrows. Swinging of the proton donor for the reprotonation from the cytoplasmic bulk in MAR is depicted with a double arrow. Proton delocalization areas are highlighted with blue clouds.

To simplify the analysis of general mechanisms, we show in Fig. 9 only those parts of the MAR, *Hs*BR, and *Bc*XeR structures in the ground and intermediate states related to proton transfer pathways. Below, we will outline the patterns of proton transport, while a more detailed description is available in Supplementary Text 3.

First, long HBCs interconnect the RSB and the proton donor/acceptor amino acid residues at certain moments of proton transfer. These HBCs in all three transporters serve as proton translocation pathways, allowing the RSB to control the translocation. The conserved amino acid residues that make up these chains have finely tuned proton affinities and unique quantum mechanical properties, like the presence of π electrons, important for proton transport (Supplementary Text 3). In MAR, the HBCs are formed in the N state in the extracellular part and exist until the relaxation of the O state when the proton transfer from the His^51^-Asp^72^ pair to the extracellular space occurs. In *Hs*BR, the chain is formed in the L state in both cytoplasmic and extracellular parts. It is used for RSB deprotonation to Asp^85^ and signaling to the Glu^194^-Glu^204^ pair for its deprotonation to the extracellular space. The continuous HBC is broken in the M state and restored in the N state for the reprotonation of RSB from Asp^96^. Last, in *Bc*XeR, the HBC from the RSB to Glu^34^ is formed in the L state to translocate protons to the cytoplasm. In general, one can deduce that the HBCs are formed just when they are necessary for proton transfer.

Second, in such HBCs, some H-bonds are SHBs with completely different properties (like the common electron cloud and the decrease in hydrophilicity of the one-dimensional chain of waters; see Supplementary Text 3 for details) compared to the regular H-bonds. The data suggest that the SHBs serve for proton storage and block proton backflow. In *Hs*BR, the proton storage pair Glu^194^-Glu^204^ is placed close to the extracellular bulk at the end of the HBC. The SHBs keep high p*K*_a_ (*3*), allowing it to store the proton and preventing the backflow of protons. In MAR, the corresponding group at the same position is missing. Nevertheless, His^51^-Asp^72^, found at the other end of the HBC, probably plays a similar role, as discussed in the text. The situation is less evident for inward proton pumps. Resolution of our data on *Bc*XeR does not allow us to clearly indicate SHBs between its residues in the proton release pocket in the cytoplasmic part, but a couple of H-bonds, nevertheless, have boundary values [2.6 Å for Thr^88^-Asp^214^ and 2.5 Å for Glu^34^-w804; PDB ID: 7ZMY (*20*)]. In contrast to outward proton pumps, in inward pumps, proton release occurs in the cytoplasm, where the local membrane concentration of protons is smaller than on the extracellular side of the membrane. Thus, there is no need to keep the p*K*_a_ of proton storage groups at very high values [which is in agreement with earlier data (*78*, *79*)], and SHBs, if they exist in inward pumps, may play a different role.

Last, both HBCs and SHBs are controlled by the RSB and its accessibility switch. In the outward pumps, retinal isomerization upon photon absorption results in the reorientation of the protonated RSB from the hydrophilic environment provided by the RSB-proximal aspartates to the hydrophobic environment in the cytoplasmic part of the transporters. This leads to the deprotonation of the RSB to primary proton acceptors (Asp^85^ in *Hs*BR and His^51^-Asp^72^ in MAR), which happens along the transition to the M state. After proton transfer, RSB directly binds to the proton donor via HBC, as seen in the N state of *Hs*BR. Deprotonated RSB controls the gradient and the injection of the protons from the proton donors (Asp^96^ in *Hs*BR and Glu^83^ in MAR) to the HBC and the following reprotonation of RSB. Reisomerization of the retinal switches the protonated RSB back to the extracellular side of the proteins. Thus, the protonated proton acceptor finds itself near the positively charged RSB, resulting in its deprotonation and the proton transfer to the bulk. Proton injection and the following transfer along the extracellular part of HBC are controlled by a switch of the protonated RSB toward the extracellular part and its integration in the HBC. However, it is not only the retinal that changes orientation—the protein also rearranges itself, starting from the opening to the extracellular part (ground state to M state), then to the cytoplasmic part (N state to O state), and back again at the end of the photocycle. This important step controls the p*K*_a_ of the essential amino acid residues and also lowers a barrier for proton transfer by changing the residues’ accessibility to bulk water. The reorientation is achieved through the cytoplasmic bulge, a special bend in the middle of the pumps, implemented differently in these transporters (see Supplementary Text 3 for details).

We assume that these elements or similar ones should also be present in other rhodopsin proton transporters. Being encouraged by the obtained data on these three transporters, we recognize that the knowledge of the key elements of the proton transfer does not yet mean that we completely understand their function on a quantum mechanical level. We do not know how exactly the protons move along HBCs and how they come and leave the SHBs. We also do not know whether the SHBs are low-barrier H-bonds (*58*, *80*–*82*). We hope that our work will highly motivate and guide neutron crystallography and subangstrom-resolution x-ray crystallography, which will provide a better understanding of the fundamentals of HBCs and SHBs and their role in nature.

## MATERIALS AND METHODS

### Phylogenetic analysis and sequence alignment

The MRh phylogenetic tree was constructed using 392 MRh sequences aligned using MAFFT-linsi (*83*), and the maximum likelihood phylogeny was constructed using iqtree2 (*84*) with automatic model selection (*85*), ultrafast bootstraps, and SH-aLRT tests [-bb 1000 -alrt 1000; (*86*)].

Another phylogenetic tree was used for sequence analysis of PRs and their closest homologs. The phylogenetic tree was constructed using a reference set composed of characterized sequences (*35*) by aligning them using the MUSCLE algorithm (*87*) in the UGENE 49.1 software package (*88*) with standard parameters. The phylogenetic tree was constructed using the PhyML maximum likelihood method and SH-like branch support and visualized using iTOL software (*89*).

### MAR expression plasmid

*Candidatus* Actinomarina minuta opsin gene (UniProt ID S5DM51) was cloned from metagenomic fosmid MedDCM-OCT-S44-C50 (*16*) without any optimization. The gene was introduced into the pIVEX2.3d vector via Nco I and Sma I restriction sites added using polymerase chain reaction (PCR) (forward primer: 5′-AAAACCATGGAAGAACTAACATATCGTCTCTTTATGGTAGC-3′; reverse primer: 5′-ATATCCCGGGCGAAACTTTTTCTCCTGACTGAACTCGAG-3′). Subsequently, the gene was transferred to the pSCodon1.2 vector (StabyCodon T7, Eurogentec) via Xba I and Bam HI restriction sites, resulting in the pSC-*MAR*-His6 expression plasmid. Consequently, the expressed construct harbored an additional C-terminal tag with a sequence PGGGSHHHHHH (full molecular weight, 25.4 kDa). The plasmid sequence is available in data S1.

### MAR expression and purification

*E. coli* strain SE1 cells (StabyCodon T7, Eurogentec) were transformed with the pSC-*MAR*-His6 plasmid. The cells were grown at 37°C in shaking baffled flasks in an autoinducing medium ZYP-5052 (*90*) containing ampicillin (100 mg/liter). After the glucose level in the growing bacterial culture dropped below 10 mg/liter, 10 μM all-*trans* retinal (Sigma-Aldrich, US) was added, the incubation temperature was reduced to 20°C, and incubation continued overnight. Collected cells were disrupted using the M-110P Lab Homogenizer (Microfluidics) at 172 MPa in a buffer containing 20 mM tris-HCl (pH 8.0), 5% glycerol, 0.5% Triton X-100 (Sigma-Aldrich, US), and deoxyribonuclease (50 mg/liter; Sigma-Aldrich, US). The membrane fraction of cell lysate was obtained by ultracentrifugation at 90,000*g* for 1 hour at 4°C. The pellets were resuspended in a 50 mM sodium potassium phosphate buffer (NaKPi; pH 8.0), 0.1 M NaCl, and 1% *n*-dodecyl-β-d-maltoside (DDM; Anatrace, Affymetrix, US). The mixture was left overnight for solubilization. The insoluble fraction part was removed by ultracentrifugation at 90,000*g* for 1 hour at 4°C. The supernatant was loaded on the Ni-NTA column (Qiagen, Germany), and the His-tagged protein was eluted in a buffer containing 50 mM NaKPi (pH 7.5), 0.1 M NaCl, 0.5 M imidazole, and 0.2% DDM. The eluate was subjected to size-exclusion chromatography (125 ml of Superdex 200 PG, GE Healthcare Life Sciences, US) in a buffer containing 50 mM NaKPi (pH 7.5), 0.1 M NaCl, and 0.01% DDM. Colored protein fractions were collected and concentrated to 40 mg/ml for crystallization.

### MAR-ARF1_L8K,Q71L_ molecular biology, expression, and purification

Bovine *Arf1* gene was amplified from the construct used in a previous study (*91*). *MAR* and *Arf1* genes were fused into a single gene by PCR. Then, we introduced the fusion genes into the pEKT expression vector, a pET vector derivative (Novagen), via Xba I and Xho I restriction sites and appended at the 3′ terminus of an additional GSGGSHHHHHH tag, which was used for metal-affinity chromatography purification. Point mutations of Arf1, L8K and Q71L, were introduced by PCR. The plasmid sequence is available in data S1.

We expressed the fusion protein in *E. coli* C41 (DE3) cells (Lucigen). The cultivation of the cells, cell disruption, and solubilization of the fusion protein were essentially the same as for MAR. The supernatant after solubilization was loaded on the Ni-NTA column (Qiagen, Germany), and after washing the column, we eluted the protein in a buffer containing 50 mM NaH_2_PO_4_/Na_2_HPO_4_ (pH 8.0), 100 mM NaCl, 0.5 M imidazole, 1 mM MgCl_2_, 10 μM guanosine diphosphate, and 0.3% DDM. Then, we applied the concentrated protein to a 30-ml Superdex 200i (GE Healthcare, Germany) column equilibrated with 50 mM NaH_2_PO_4_/Na_2_HPO_4_ (pH 8.0), 100 mM NaCl, 1 mM MgCl_2_, 10 μM guanosine diphosphate, and 0.2% DDM buffer and pooled a peak of colored functional protein. Last, we concentrated homogeneous protein to 40 mg/ml for crystallization.

### Measurement of pumping activity of MAR in *E. coli* cells and in liposomes

The pumping activity of MAR in *E. coli* cells was measured as described previously for the sodium pump KR2 (*92*). Reconstitution of MAR into soybean phospholipids and measurements of its pumping activity in liposomes were performed as described previously for the inward proton pump *Bc*XeR (*78*).

### Preparation of single lipid vesicles for planar BLM experiments with MAR

Phospholipids (asolectin from soybean, Sigma-Aldrich, US) were dissolved in chloroform (Chimmed, Russia) at a concentration of 1% (w/v) in a pear-shaped glass flask. Then, the solvent was completely evaporated under vacuum using a rotary evaporator, and a thin lipid film on the sides of the flask was formed. The residual solvent was removed using a vacuum pump overnight. The dried lipids were resuspended in 0.1 M NaCl (AppliChem, Germany) supplemented with 2% (w/v) sodium cholate (Sigma-Aldrich, US) at a final concentration of 1% (w/v). The mixture was clarified by sonication at 4°C for 5 min, and solubilized MAR (50 mg/ml) was added to a final protein concentration of 0.7 mg/ml. The detergent was removed by stirring with detergent-absorbing beads (Amberlite XAD-2, Sigma-Aldrich, US) at 4°C and minimal light exposure. Four changes of beads were performed for the total removal of detergents.

### Planar BLM experiments with MAR

The BLM setup was similar to that described by Bamberg and co-workers (*5*, *93*). The planar BLM was formed from a solution of 1,2-di-*O*-phytanoyl-*sn*-glycero-3-phosphocholine (20 mg/ml, Avanti Polar Lipids, US) and 1,2-dimyristoyl-*sn*-glycero-3-ethylphosphopholine in *n*-decane (0.4 mg/ml, Avanti Polar Lipids, US) on a 0.8-mm aperture in a septum separating the experimental Teflon cell into two compartments of equal size (each of 3-ml volume). The compartments were filled with a buffer containing 10 mM MES (Sigma-Aldrich, US) and 10 mM NaCl (AppliChem, Germany) with the required pH, starting from pH 5.0, adjusted by tris (Sigma-Aldrich, US) under stirring. The cell was connected to an external measuring circuit via two Ag/AgCl electrodes, which were placed on both sides of the BLM. The patch-clamp amplifier (OES-2, OPUS, Russia) and the output signal digitizer NI-DAQmx (National Instruments, US) were used for electric current measurements. Data analysis was conducted using the WinWCP Strathclyde Electrophysiology software designed by J. Dempster (University of Strathclyde). BLM was exposed to continuous illumination with a halogen lamp (Novaflex, World Precision Instruments, US), providing an incident power density of 0.8 W/cm^2^. MAR-containing liposomes (20 μl) were added to one of the cell compartments and thus adhered to one side of the BLM. Then, 0.77 μM tetrachloro-2-(trifluoromethyl)benzimidazole [a gift of L. Yaguzhinsky (Moscow State University)] was added to the other compartment of the cell. The photocurrents were recorded after incubation of liposomes during 1 hour. All the experiments were held at 25°C.

### Time-resolved absorption spectroscopy experiments with MAR

The laser flash photolysis setup was performed similarly to previous work (*94*–*96*). A Surelite II-10 Nd:YAG laser (Continuum Inc., US) was used for providing pulses of the duration of 5 ns at a 532-nm wavelength and an energy of 3 mJ per pulse. Samples in spectroscopic quartz cuvettes (5 by 5 mm; Hellma GmbH & Co., Germany) were placed in a thermostated house between two collimated and mechanically coupled monochromators (1/8-m model 77250, Oriel Corp., US). The probing light (Xe-arc lamp, 75 W, Osram, Germany) passed the first monochromator sample and arrived after a second monochromator at a photomultiplier tube detector (R3896, Hamamatsu, Japan). The current-to-voltage converter of the photomultiplier tube determines the time resolution of the measurement system of ~50 ns (measured as an apparent pulse width of the 5-ns laser pulse). Two digital oscilloscopes (LeCroy 9361 and 9400A; 25 and 32 kilobytes of buffer memory per channel, respectively) were used to record the traces of transient transmission changes in two overlapping time windows. The maximal digitizing rate was 10 ns per data point. Transient absorption changes were recorded in the time window from 0.7 μs to 1 s. Twenty-five laser pulses were averaged at each wavelength to improve the signal-to-noise ratio. The quasi-logarithmic data compression reduced the initial number of data points per trace (~50,000) to ~600 points evenly distributed in a log timescale, giving ~100 points per time decade. The wavelengths were varied from 330 to 730 nm in steps of 10 nm (together, 41 spectral points) using a computer-controlled step motor. Absorption spectra of the samples were measured before and after each experiment on a standard spectrophotometer (Beckman DU-800).

For the experiment in solution, MAR was reconstituted into nanodiscs as described in (*78*). Overall, 18 datasets were obtained. The temperature of the sample was varied from 0° to 50°C in 10°C steps. The samples were suspended in buffers containing 0.2 M NaCl and 50 mM NaKPi (pH 5, 7.5, or 10). Each dataset was independently analyzed using the global multiexponential nonlinear least-squares fitting program MEXFIT, similar to previous work. In all the photocycles, at least five intermediates are needed for a reasonable fit (figs. S3 to S5). The temperature dependence of the five apparent rate constants is presented in fig. S6. Apparent activation enthalpies and entropies of reactions obtained from the fit are summarized in table S1.

A similar setup was used for time-resolved absorption spectroscopy in crystals, except that the homemade LCP plate holder replaced the thermostatic cuvette holder. In the holder, the plate at ambient temperature is inclined at an angle of 45° to the horizontal position so that, first, the horizontal probe beam can pass through the plate and, second, the vertical excitation laser beam can reach the crystals. The selected well was investigated with an optical microscope to detect the crystal positions in the drop. The area around the crystals was covered by nontransparent tape to make light pass only through the crystals.

### Crystallization and x-ray crystallography

The crystals of MAR and MAR-Arf1_L8K,Q71L_ were grown using the in meso approach (*32*), similar to our previous works (*3*, *7*, *11*, *48*,*68*, *78*, *92*, *97*–*99*). The solubilized protein was mixed with the monooleoyl-formed lipidic phase (Nu-Chek Prep, US) and the crystallization buffer was added. Crystallization trials were set up using the NT8 robotic system (Formulatrix, US). The crystals were grown at 22°C and reached the final size of 100 to 300 μm within 1 to 3 months. Overall, three crystal forms appeared in our crystallization trials. Orange-form crystals of MAR (fig. S7A; corresponding to the ground and M-like state structures) belong to the *P*1 space group and contain two MAR molecules in the asymmetric unit (ASU). Crystals of MAR-Arf1_L8K,Q71L_ (fig. S7C; this crystal form was used to accumulate the N state intermediate of MAR) belong to the *C*2 space group and contain one chimera molecule in the ASU. Rose-form crystals of MAR (fig. S7B; corresponding to the O* and O state structures) belong to the *P*2 space group and contain one MAR molecule in the ASU. The best orange-form MAR crystals were obtained in 3.0 M ammonium phosphate buffer (AmPi pH 8.8; ground state), 1 M ammonium sulfate, and 0.1 M sodium acetate (pH 5.2; M-like state). The best MAR-Arf1_L8K,Q71L_ crystals were obtained in 2.0 M AmPi (pH 7.0). The best rose-form MAR crystals were obtained in 2.6 M AmPi (pH 8.4; O* state), 140 mM NaCl, 10% polyethylene glycol, molecular weight 600, and 0.1 M sodium acetate (pH 4.6; O state). All crystals were harvested using micromounts (MiTeGen), flash cooled, and stored in liquid nitrogen.

Diffraction data were collected at 100 K at the European Synchrotron Radiation Facility (ESRF), Grenoble, France, beamlines ID23-1 (MAR in the ground, M-like, O*, and O states), ID30A-3 (MAR-Arf1_L8K,Q71L_ in the N state), and ID30B (MAR-Arf1_L8K,Q71L_ in the ground state); and at the PETRAIII, DESY, Hamburg, Germany, EMBL beamline P14 (MAR in the P593 state). Diffraction images were processed using XDS (*100*). Because of the data’s notable anisotropy, excluding the M-like state, the STARANISO web server was used to estimate the anisotropic resolution limits (table S2) and apply an anisotropic correction. Despite the lower completeness of the resultant dataset at high-resolution shells, this service provides better quality of the final models than those built using all the reflexes. It is regularly used in the studies of challenging proteins [see refs. (*101*–*104*)]. For the M-like state of MAR, POINTLESS and AIMLESS (*105*) were used instead. When allowed by the space group (PDB IDs: 8RSO/7AVN/8RSP, 8RSQ/8RSR, and 8RSS/7AVP), the test set was chosen the same between different structures. The structures of MAR were solved using molecular replacement with MOLREP (*106*) and the poly-ala MAR [PDB ID: 5JSI (*107*)] as a search model. The structure of MAR-Arf1_L8K,Q71L_ was solved with Phaser, where poly-ala structures of MAR [PDB ID: 5JSI (*107*)] and human Arf1 [PDB ID: 1HUR (*108*)] were used as search models. The resultant structures were then rebuilt in Phenix.Autobuild (*109*). Interactive refinement was performed in Coot (*110*). REFMAC5 (*111*) and Phenix.Refine (*112*) were used for the automatic refinement. The final quality was assessed with Phenix.MolProbity (*113*). The final resolution of the models was confirmed by the paired refinement approach (*114*). In the case of the structure of MAR-Arf1 double mutant in the N state, the resolution dropped from 2.1 to 2.3 Å. The cavities were calculated using HOLLOW (*115*). Hydrophobic-hydrophilic boundaries of the membrane were calculated using the PPM server (*116*). Because of the low occupancy of the cryotrapped O state, obtained from the O* state crystals, we used extrapolated maps built in Xtrapol8 (*117*) to build the corresponding model.

### Identification of SHBs

Our data on MAR do not allow direct visualization of H-atoms, which is important for distinguishing between regular H-bonds and SHBs. Therefore, we analyzed the lengths of H-bonds to trace SHBs. For accurate analysis, the coordinate errors of the relative positions of the atoms were calculated as proposed in (*118*). Briefly, the position (*r*) error of an atom was estimated as σ*_r_* = 3^1/2^[*N*_atom_/(*N*_refl_ − *N*_param_)]^1/2^*C*^−1/3^*R*_factor_*D*_max_ = 3^1/2^ DPI, where *N*_atom_ is a total number of atoms in the structure, *N*_refl_ is a total number of reflections included in refinement, and *N*_param_ is the number of parameters of the model with the *R* factor of the model *R*_factor_, resolution limit *D*_max_, completeness *C*, and diffraction-component precision index DPI. The Cruickshank DPI in the formula is calculated with REFMAC5 (*111*) and included in table S3 for each structure. The H-bond length error was estimated as σ_d_ = 2^1/2^σ*_r_* = 6^1/2^ DPI. In the case of lower-resolution MAR-Arf1_L8K,Q71L_ structures, model restraints start to play a substantial role, and a free *R* factor *R*_free_ better estimates the H-bond length error with another formula σ_d_ = 6^1/2^(*N*_atom_/*N*_refl_)^1/2^*C*^−1/3^*R*_free_*D*_max_. For the ground and O states, the σ_d_ value was at the level of 0.1 Å, and for the M-like and O* states of MAR, it was 0.2 Å, while for the N state of MAR-Arf1_L8K,Q71L_, it was 0.3 Å. Given that H-bonds with the O–O or N–O distances of 2.3 to 2.6 or 2.3 to 2.7 Å, respectively, are considered SHBs (*119*, *120*), we assigned H-bonds as SHBs only when the distance was smaller than 2.5 or 2.6 Å for the ground and O state structures and 2.4 or 2.5 Å for the M-like and O* state structures. No SHB analysis was performed for the other structures because of the high H-bond length error. Information on SHBs in the structures of *Hs*BR and *Bc*XeR was taken from refs. (*3*, *20*), respectively.

### In crystallo UV-Vis absorption spectroscopy and cryotrapping of intermediate states

Absorption spectra of MAR crystals were collected at the in crystallo optical spectroscopy (*ic*OS) laboratory at the ESRF (*121*). Ultraviolet-visible (UV-Vis) absorption spectra were measured using a DH-200-BAL deuterium-halogen lamp (Ocean Optics, Dunedin, FL) as a reference light and a QE65 Pro spectrophotometer (Ocean Optics, Dunedin, FL). The crystal, mounted in a loop on the *ic*OS diffractometer, was placed in the flow of a cryostream (Oxford Instruments, UK) maintained at 100 K in the coinciding focal volume of three reflective objectives. The white lamp was connected to one of the objectives through a 200-μm-diameter fiber, resulting in a 50-μm focal spot on the crystal. The spectrometer was connected to the opposite objective with a 400-μm-diameter fiber. Spectra were recorded with a 100-ms acquisition time and averaged 20 times.

To populate the N and P593 states and show the ability of the O* state crystals to convert to the O state, we used the cryotrapping approach at the *ic*OS laboratory. Initially maintained at 100 K, a crystal was illuminated with a 532-nm laser (CNI Laser, Changchun, P.R. China) via the third objective for 2 s at room temperature by blocking the cryostream. The laser was connected through a 600-μm-diameter fiber, resulting in a 150-μm-diameter focal spot and a power density of 2 mW/cm^2^ at the crystal position. The mean size of the crystals was 100 by 50 by 20 μm. During the cryotrapping, the plate-like crystals were oriented so that the largest plane (100 by 50 μm) was perpendicular to the laser beam. The spectral data were analyzed using the in-house Python script (https://github.com/ncara/icOS). A similar cryotrapping setup was recreated for the x-ray data collection at the beamlines ID30A-3 (ESRF) and P14 (EMBL Hamburg).

## Supplementary Materials

### The PDF file includes:

Supplementary Text Figs. S1 to S19 Tables S1 to S5 Legend for movie S1 Legend for data S1 References
