# Supplementary material for: Proteorhodopsin insights into the molecular mechanism of vectorial proton transport
Source: Sci Adv. 2025 Apr 16;11(16):eadu5303. doi: 10.1126/sciadv.adu5303 (PMC12002130; doi:10.1126/sciadv.adu5303)
Supplement: Supplementary file 1 — Supplementary Text Figs. S1 to S19 Tables S1 to S5 Legend for movie S1 Legend for data S1 References [file sciadv.adu5303_sm.pdf]

Science Advances
AAAS

Supplementary Materials for

Proteorhodopsin insights into the molecular mechanism of vectorial proton transport

Sergey Bukhdruker *et al.*

Corresponding author: Valentin Gordeliy, valentin.gordeliy@ibs.fr

Sci. Adv. **11**, eadu5303 (2025)
DOI: 10.1126/sciadv.adu5303

The PDF file includes:

Supplementary Text
Figs. S1 to S19
Tables S1 to S5
Legend for movie S1
Legend for data S1
References

Other Supplementary Material for this manuscript includes the following:

Movie S1
Data S1

Supplementary Text

Supplementary Text 1**.** Retinal isomerization in the O state of different light-driven outward proton pumps.

The topic of retinal isomerization in different states of rhodopsins photocycle is complex, and many of the data interpretation problems come from the absence of high-resolution data of the late intermediates. The most cited Raman spectroscopy paper shows that in the O state of *Hs*BR (absorption maximum at 640 nm), the retinal, like in PRs, is in the all-*trans* configuration (*121*). However, recent spectroscopical studies (*123*) argue that the O state of *Hs*BR, in fact, is comprised of two states: O_1 (600 nm) and O_2 (635 nm). In the O_1 state, the retinal is likely still in the distorted 13-*cis* configuration, while in the O_2 state, it is in the all-*trans* state. What is important for us is that in both *Hs*BR and PRs, proton transfer to the extracellular space proceeds on the last stage of the photocycle after the retinal is relaxed back to the all-*trans* state. The concrete name of the last intermediate (O state in PRs or O_2 state in *Hs*BR) here is not critical. Thus, the structure of the O state of MAR (as a good model for the O_2 state in *Hs*BR) allows us to study the last stage of the photocycle of outward proton pumps, when the proton pumping to the extracellular space occurs.

Supplementary Text 2. Crystal contacts limit the functionality of the MAR molecules in the orange form crystals.

Initially, we worked with the orange form MAR crystals at high pH, containing the most native form of the protein in the ground state that we had, indicated by the major spectral maximum (λ = 515 nm; fig. S15A) and structural organization (RSB region organization, accessibility switch opened to the extracellular space). However, we quickly realized that the crystal contacts restrict the functionality of the orange form MAR crystals. This can be seen, e.g., by the photocycle of this crystal form, having a P593 late intermediate state (λ = 593 nm) not present for MAR in nanodiscs at any studied pHs (fig. S16). Moreover, the crystal spectrum indicates that a portion of MAR was already in the P593 state before the laser activation (fig. S15A). To understand what this state corresponds to, we need to discuss these crystals' structure and spectrum in more detail than was done in the main text.

ASU of the orange form MAR crystals at high pH comprise two MAR molecules that are not identical (fig. S15B). Molecule A was described in the main text. Briefly, the structural organization of the RSB region in the molecule was found to be identical to that in *Hs*BR, having three water molecules and the deprotonated His51-Asp72 pair (fig. S15C). Such a molecule gives rise to the spectral peak at λ = 518 nm, corresponding to the ground state. However, molecule B is different. Its RSB region comprises two conformations. Conformation A (occupancy equal to 50%) is identical to the one observed for molecule A and corresponds to the ground state. Conformation B (occupancy equals to 50%; we will further refer to it as P593) is somewhat similar to the conformation A of the M-like state, having only one water molecule in the RSB region, bonded His51-Asp72 pair, and all-*trans* retinal. However, there are remarkable differences in the proton-release region. In conformation A of the M-like state, Arg69 shifts towards the RSB region, losing its H-bonds with Glu189 and replacing the gone water molecule w3. In the P593 state, both Arg69 and Glu189 move simultaneously, preserving their H-bonds. As a result of such motion, Arg69 loses its H-bond to Thr193, while Glu189 loses the bond to Thr66. Thus, P593 is a unique state that exists in the orange form MAR at high pH and is different from both the ground and the M-like states discussed in the main text.

We then tried to use cryotrapping at RT with these crystals to solve the structure of an intermediate state. We observed the redshift of the spectrum (fig. S15A). When solving the structure, we saw no changes in molecule B; however, molecule A partially transited to the P593 state (occupancy being around 50%). This indicates that illumination with the laser-induced transition of the ground state to the P593 state in this molecule. Given that we know the occupancies of the ground and the P593 states for the unilluminated crystals (Occupancies of the ground:P593 states being 75%:25%), we can estimate occupancies after cryotrapping using spectral data. To achieve this, we simultaneously fitted two spectra with three Gaussians: λ_{max} = 367 nm (LCP scattering and/or free retinal), λ_{max} = 518 nm (ground state), and λ_{max} = 593 nm (P593 state). According to our calculations, the ground:P593 states

occupancies are 56%:44% (±6%), which correlates with the occupancies of the corresponding states in the refined structure. Thus, illumination of the orange form MAR crystals at high pH induces the transition of the protein from the ground state to the P593 state, indicated by both the structural and spectral data.

Finally, it was important to understand why MAR in the orange form crystals transits to the artificial P593 state. We suggest that it could be due to the crystal contacts that do not allow the accessibility swich to occur. As a result, after isomerization and proton loss to the His51-Asp72 pair, the RSB gets reprotonated not from the cytoplasmic bulk but likely from a residue from the extracellular side. While we do not exclude that this could happen in solution, the reverse reaction is inhibited thanks to the accessibility switch.

To summarize, while molecule A of the crystals of MAR in the orange form at high pH likely resembles the ground state of the protein, this crystal form should not be used to study intermediate states. This is because the protein, after activation, transits to the P593 state, which is not present in the solution. While this state resembles the structure of the M-like state, it is an artifact of the dense crystal packing, not allowing the accessibility switch to happen. The situation is completely different for MAR-Arf1 mutant crystals. After laser activation at RT, they transit to the singular state with the peak at λ_{max} = 550 nm. At the same time, the structure of MAR in this crystal form shows the signs of the accessibility switch to the cytoplasm, allowing us to assign the state as the N state.

Supplementary Text 3. Details of proton translocation in different proton transporters

Cytoplasmic part of the pumps. Proton transport in both MAR and *Hs*BR depends on the ability of the transporters to reorient from the extracellular side to the cytoplasm and back. This is achieved by cytoplasmic bulge, which is implemented differently in these transporters. Similarly to the case of other PRs (*7*, *34*), the structure of the middle part of the MAR helix F deviates from α-helical geometry in the vicinity of Pro173 (fig. S17). In both forms (orange and rose), the deviations are stabilized by direct H-bonds between the Asn199 side chain and Gly168 carbonyl oxygen and water molecule-mediated hydrogen bonds between the Tyr136 side chain and Leu166 and Ile170 backbone atoms. In the orange form, there are also two 3_{10}-like bonds between Val167 and Trp169 carbonyl oxygens and Ile170 and Tyr172 amide groups. Gly168 carbonyl oxygen is bound to the Ile171 amide group via a structural water molecule w4 (fig. S17A). In the rose form, there are three 3_{10}-like bonds between Val167, Gly168, and Trp169 carbonyl oxygens and Ile170, Ile171, and Tyr172 amide groups (fig. S17B).

The cytoplasmic bulge (π-bulge in *Hs*BR and a conserved proline residue for PRs, Pro173 in MAR) is connected to a chain of water molecules (HBC), which is stabilized by the carbonyl group of the RSB lysine. A modified HBC may connect the RSB vicinity with the proton donor residue. As an example of such communication, we should note that a chain of SHBs appears in the M state of *Hs*BR (*4*) (Fig. 5B). It is evident by the short length of the H-bonds (2.5-2.6 Å) in the HBC connecting Asp96 and the π-bulge. This means the proton is not completely localized at the proton donor Asp96 in the M state. The protonation state of Asp96 influences the deprotonation of the RSB. It is expected since the proton donor reprotonates the RSB upon transition from the M to N state.

A direct transient HBC between the RSB and donor/acceptor in at least one of the states related to the proton transfer event is observed (Fig. 5B). Indeed, in the N state of *Hs*BR, HBC directly connects the RSB and the proton donor Asp96, allowing the proton to be transferred to the RSB upon transition from the M to the N state. In the inward pump, the *Bc*XeR proton transfer from the RSB to the cytoplasmic proton acceptor proceeds upon transition from the L to M state. An HBC directly connects the RSB and the proton acceptor in the L state. In MAR, proton transfer from the proton donor Glu83 to the RSB proceeds upon transition from the M to N state. Currently, the structure of the M state of MAR is unavailable. Nevertheless, considering the data obtained with *Hs*BR and *Bc*XeR we suggest the existence of a transient HBC directly connecting the RSB and the proton donor in the M state of MAR. Thus, the ’hydrophobic’ water HBCs can conduct protons through hydrophobic media.

It has been recognized for a long time that the cytoplasmic part of proton pumping rhodopsins between the RSB and the proton donor is quite hydrophobic. There is a wealth of work on transferring the proton through such a ‘hydrophobic’ gap (*124*, *125*). Several water molecules observed in the gap might seem unexpected (Fig. 5B). We suggest it might be possible due to the specific properties of

one-dimensional HBCs. At already short distances, comparable to the size of a water molecule (about 3 Å), the electric field of HBC will decay fast, i.e., the chain of water molecules may become less hydrophilic. Our hypothesis on the 'hydrophobicity' of HBC explains several water molecules' appearance in the proteins' hydrophobic gaps. It is also supported by the existence of highly proton-conductive hydrophobic channels in nanomaterials such as hydrophobic carbon nanotubes (*126*). Anomalous proton permeability through lipid membranes is additional evidence in favor of the hypothesis. The proton permeability coefficients at neutral pH are enormously high. It is six to seven orders of magnitude greater than expected from the data on other monovalent cations (*127*).

Extracellular part of proton pumps. In outward proton pumps, long one-dimensional HBCs connect proton acceptors (Asp72 of MAR and Asp85 of *Hs*BR) and proton release group (Glu189 in MAR and Glu194-Glu204 pair in *Hs*BR) through Arg residues (Arg69 in MAR and Arg82 in *Hs*BR) (Fig. 9). In the inward proton pump, *Bc*XeR, a similar HBC, connects proton acceptor Asp73 and proton uptake residue Glu195 through Arg70 (Fig. 9). It is noteworthy that in all these cases, arginine is H-bonded to one half of the HBC via its NE atom and to the other half via its NH1 or NH2 atoms. The Arg side chain may flip during the photocycle, switching its NH1 and NH2 atoms from one half of the HBC to the other, leading to the proton transport. First, the Arg NH1 or NH2 atom donates one of its protons to one half of the HBC, and after the Arg flips and forms an H-bond with NH1/NH2 to the other half, the deprotonated atom is reprotonated. We also do not exclude a quantum mechanical nature of the proton transfer from the NE atom to the NH1 and NH2 atoms without the need for a flip of the Arg side group, and we describe this possibility in the text below.

Despite quite remarkable structural and functional differences of the proteins, the HBCs are amazingly similar. As we have seen above, at the ends of all HBCs, there is Asp and, in the proton release region, then Arg, followed by a Glu residue. Moreover, two more residues of the outward pumps, Asp196 and Tyr52 in MAR and Asp212 and Tyr57 in *Hs*BR, comprising the HBCs, are also the same. The residue similar to Asp196 and Asp212 in the inward proton pump is absent. Nevertheless, residue Tyr49 is present. All the mentioned residues (His, Asp, Tyr, Arg, and Glu) occupy identical positions in the structures of the proteins (Fig. 9), and all of them are conserved. This is not surprising; all the components of HBCs must match each other to create a continuous one-dimensional wire. The first requirement for such a wire is that the size of each chain must correspond to the length of H-bonds. The second requirement is that the HBCs must be able to conduct the signals from one end of the HBCs to the other. Third, the HBC must be able to transfer the protons between the ends of the wires. All these conditions impose restrictions on the content of the HBCs, explaining the conservation of functional amino acid residues.

Notably, conserved residues in such HBC and RSB itself possess π-electrons. π-electron clouds are critical to provide a continuous electron density connection between the ends of the HBC to transfer

the signals and protons. As an example, we will discuss arginine residue and its role in proton transport in *Hs*BR. Arg82 in *Hs*BR plays a key role in signaling and a subsequent proton transfer step. The functional group of arginine is the guanidinium cation, pointing towards the possible quantum mechanical nature of the proton transfer by the residue. Two pairs of π-electrons are distributed evenly between three nitrogen atoms in non-bonded Arg residues. A positive charge is also evenly distributed among nitrogens. In *Hs*BR, the extracellular HBC is connected to the NE atom of Arg82 in the L, M, and N states. This reduces the symmetrical distribution of the positive charge. Moreover, one can expect that the protonation state of Asp85, located at the opposite end of the HBC, will favor a particular distribution of the electrons, resulting in the shift of the positive charge more towards the NH1 or NH2 ends of Arg82. This charge redistribution breaks H-bonds between NH2 of Arg82 and Glu194 and establishes two H-bonds between NH1, NH2, and Glu204 during the L to M state transition. Consequently, the proton stored in the proton-release group Glu194-Glu204 is released (*3*). This quantum mechanical property of the guanidinium group explains the surprising conservation of arginines in MRhs.

Finally, considering the unique properties of the conserved residues comprising proton transfer chains, we can now discuss the proton transfer mechanism. In both *Hs*BR and MAR, proton transfer from the proton acceptor to the proton-release group occurs upon the O to the ground state transition after retinal relaxed back to all-*trans* conformation [see refs. (*50*, *121*, *122*) and Fig. 8]. The structure of the O state of *Hs*BR is still unknown. It is important that the data on MAR fill the gap in research on *Hs*BR, which is the absence of high-resolution structures of the O state intermediate. The data on MAR are complementary to those obtained with *Hs*BR. Since the structure of the O state is solved at 1.09 Å-resolution, we have a unique opportunity to show that the proton propagates along the HBC. So, how does proton transport occur in MAR? The HBC formed in the N state and preserved in the O state is very sensitive to changes in pKa at its ends due to the presence of π-electrons. When a positive proton charge appears on one side of the chain (e.g., reprotonated RSB), it can release a proton at the other end. This can occur either as a result of small flips of amino acid residues directly transferring protons or due to other mechanisms that do not require the movement of chain elements, such as those described in the works of Nagle and colleagues (*56*, *57*). To demonstrate this possibility, we present in fig. S18 a hypothetical model of proton transfer in the cytoplasmic part of *Hs*BR during the M-to-N transition based on Nagle's hop/turn mechanism. We also do not exclude that both mechanisms are involved simultaneously, as it is unclear how the proton from the His51-Asp72 pair can be passed to the HBC without a flip of one of the residues to the water molecule w1* upon the relaxation to the ground state. Neutron diffraction data and sub-angstrom-resolution X-ray crystallography will allow a more detailed understanding of the mechanism in the future.

What about proton transfer through the extracellular part of the inward proton pump *Bc*XeR? A similar HBC is present in the protein. As discussed in the main text, having a strong storage proton group in this protein part might be unnecessary. The proton transfer through the HBC proceeds upon the transition of the M to the ground state. In the M state, the RSB is deprotonated. However, unlike the outward proton pumps (*20*), the RSB is connected to the extracellular HBC (Fig. 9). The absence of the proton at the RSB creates a driving force for the inward transfer of the proton through the HBC and the following reprotonation of the RSB upon transition to the ground state.

Summary. To summarize, the concept of the proton transfer mechanism is as follows. The outward proton transport proceeds through HBCs connected to the RSB of the active sides of the transporters (extracellular for the outward pumps and intracellular for the inward pumps). The HBC in a quite hydrophobic cytoplasmic part solely consists of water molecules and provides the proton uptake. This fact points to a ‘hydrophobic’ nature of the HBC, which may also explain the existence of HBCs and anomalous proton conductivity in hydrophobic pores of nanomaterials. In contrast, the HBCs in the polar extracellular part of the proteins have a mixed composition; amino acid residues and water molecules form them. SHB at one of the ends of extracellular HBC serves for proton storage and prevention of the proton backflow.

Proton injection to the HBC and transfer is triggered through the change of boundary conditions (pK_as of the amino acids and the RSB at the ends of the HBCs) by reorientations and deprotonation/reprotonation of the RSB. It takes place upon isomerization/reisomerization of the retinal. The switch of the RSB towards the cytoplasmic side of the protein and its back switch to the extracellular side execute proton transfer in two time-separated events since stepwise proton transfer is energetically favorable. Among amino acid residues of the HBCs are only those with π-electron clouds. They allow continuous electron density along the HBCs. A specific and principal example is Arg in the extracellular HBCs. A schematic representation of the proton pumping on the example of *Hs*BR is shown in fig. S19.

Similar mechanisms are also characteristic of the inward proton pump. The only principal difference is the absence of strong proton storage pairs. Principal features of the proton transfer mechanisms in all these proteins are common.

fig. S1.

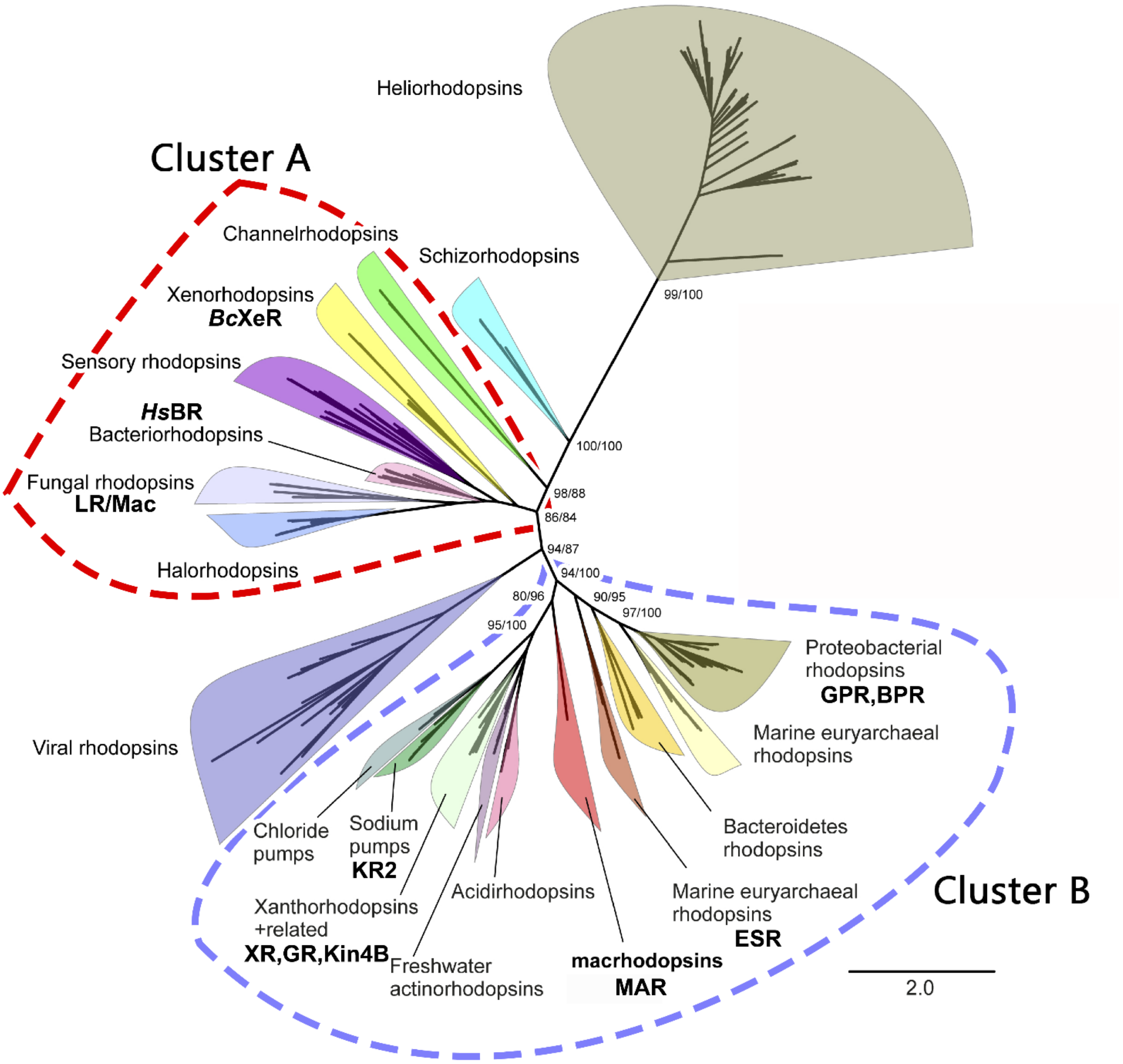

Phylogeny of microbial rhodopsins (MRhs, Type I rhodopsins). An unrooted maximum likelihood tree of all MRhs is shown. Cluster A and B rhodopsins (*4*, *23*) are highlighted with dashed lines. The clades are labeled according to phylogenetic affiliations or function (if known). The scale bar indicates the number of substitutions per site. Bootstrap values are shown at the branching sites. MRhs representatives mentioned in the text are shown in bold under their corresponding clades.

fig. S2.

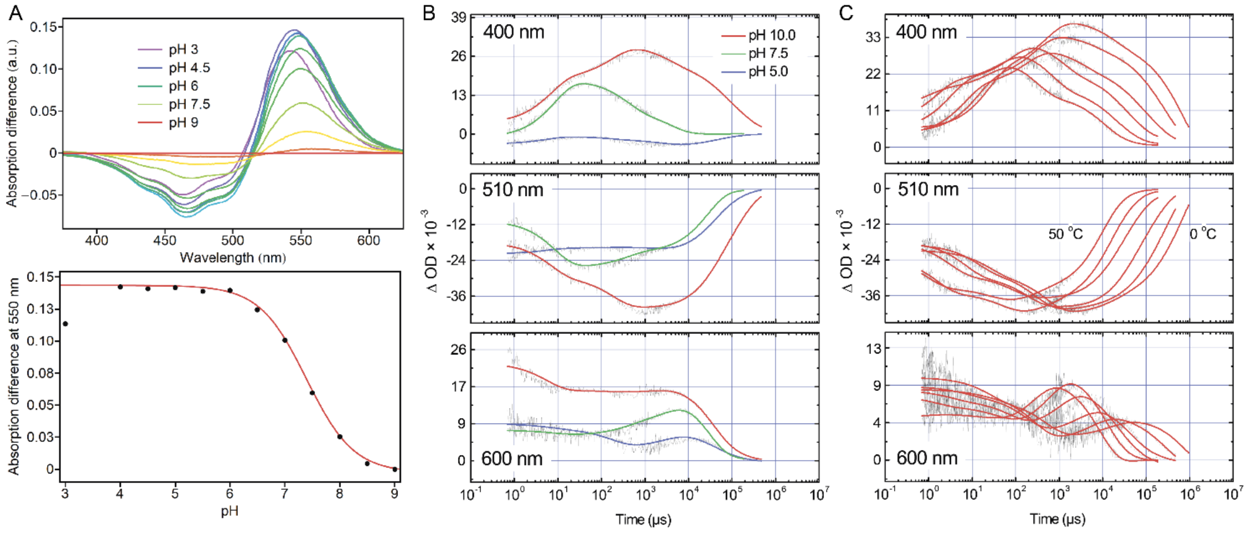

Dependence of the spectral properties of MAR on pH and temperature. (A), pH dependence of the absorption of solubilized MAR. The absorption difference spectra are shown for the pH values, starting from 9 down to 5 with a step of 1.5. The bottom panel shows the fitting of the absorption changes with the Henderson-Hasselbalch equation. Proton acceptor pK_a is determined to be 7.4. **(B)**, pH dependence of the transient absorption changes of MAR in nanodiscs (20 $^{\circ}$C) at three characteristic wavelengths. The raw data are shown in black, while red, blue, and green lines represent the result of the five-exponential fit for pH 10, 7.5, and 5.0, respectively. **(C)**, Temperature dependencies of the transient absorption changes of MAR in nanodiscs (pH 10.0) at three characteristic wavelengths 400, 510, and 600 nm, with temperature ranging from 0 to 50 $^{\circ}$C with a step of 10 $^{\circ}$C. Black lines represent the experimental data, while red lines result from global fit using five exponents.

fig. S3.

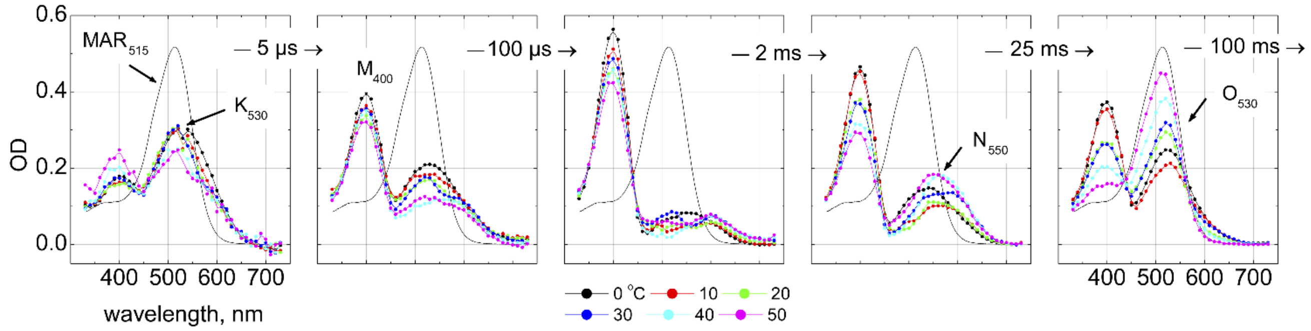

MAR photocycle spectra at different temperatures at pH 10. Each graph contains six spectra obtained at temperatures from 0 to 50 °C (in steps of 10 °C, data points, and B-spline connecting lines) and the ground MAR spectrum (thin black lines; measured at a standard spectrophotometer before experiments). The spectra were calculated by fitting the data based on a suggested sequential, irreversible reaction model. Depicted half-times correspond to 20 °C.

fig. S4.

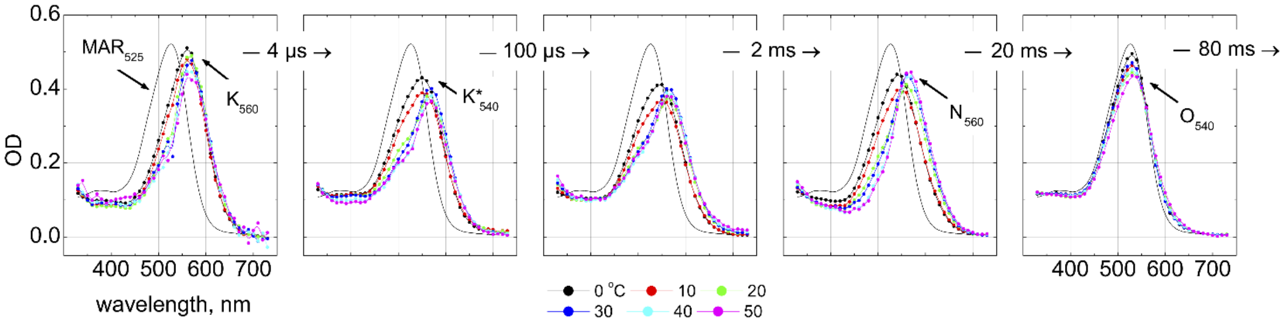

MAR photocycle spectra at different temperatures at pH 5. Each graph contains six spectra obtained at temperatures from 0 to 50 °C (in steps of 10 °C, data points, and B-spline connecting lines) and the ground MAR spectrum (thin black lines; measured at a standard spectrophotometer before experiments). The spectra were calculated by fitting the data based on a suggested sequential, irreversible reaction model. Depicted half-times correspond to 20 °C.

fig. S5.

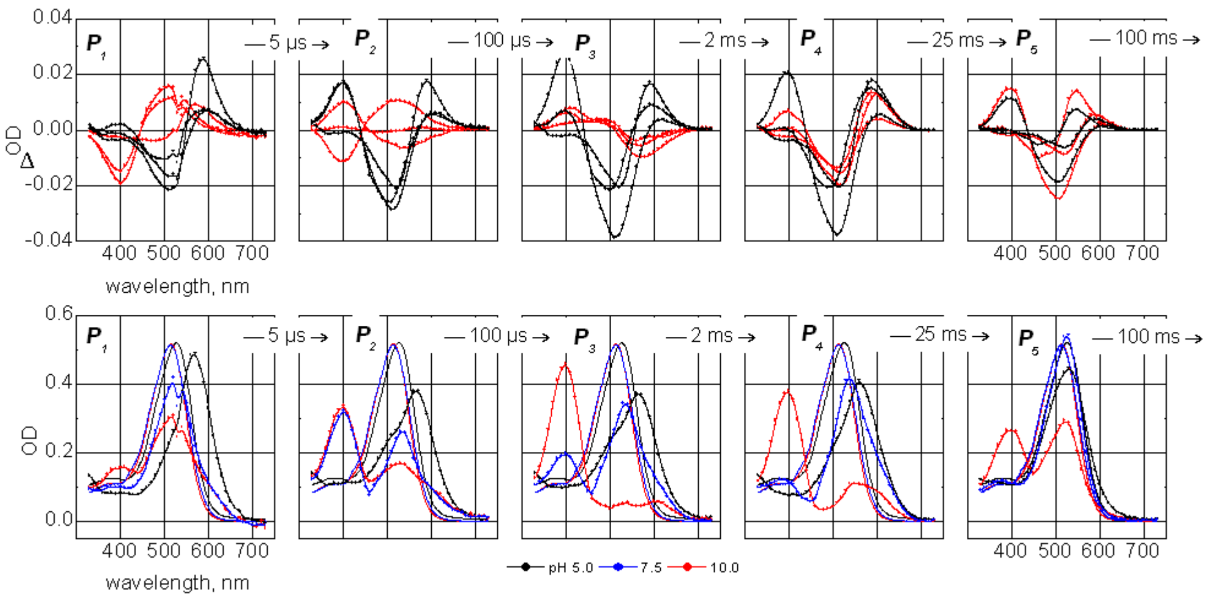

The MAR photocycle spectra at different pH values (5.0, 7.5, 10.0 at 20 °C). Upper row: amplitude spectra of five exponential components (red points and B-spline connecting lines) and corresponding difference spectra of the P_1-P_5 intermediates (with respect to the ground spectrum MAR, black points, and B-spline connecting lines). Depicted half-times correspond to 20 °C. Lower row: derived absorption spectra of intermediates P_1-P_5 of MAR.

fig. S6.

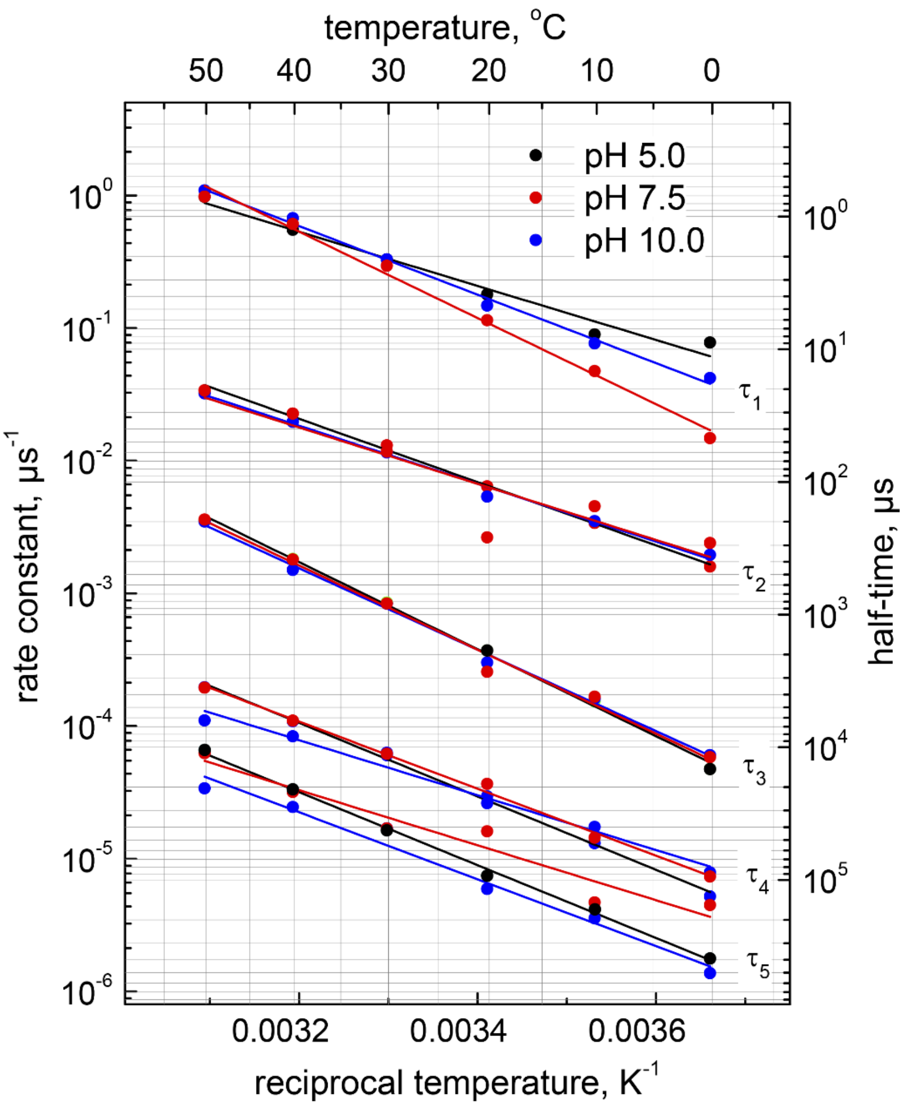

Temperature dependence of the rate constants derived from a five-exponential fit. Rate constants of the reaction in Arrhenius coordinates (left axis; right axis: corresponding half-times) at three pH (5.0, 7.5, and 10.0) (circles) and their fit (lines).

fig. S7.

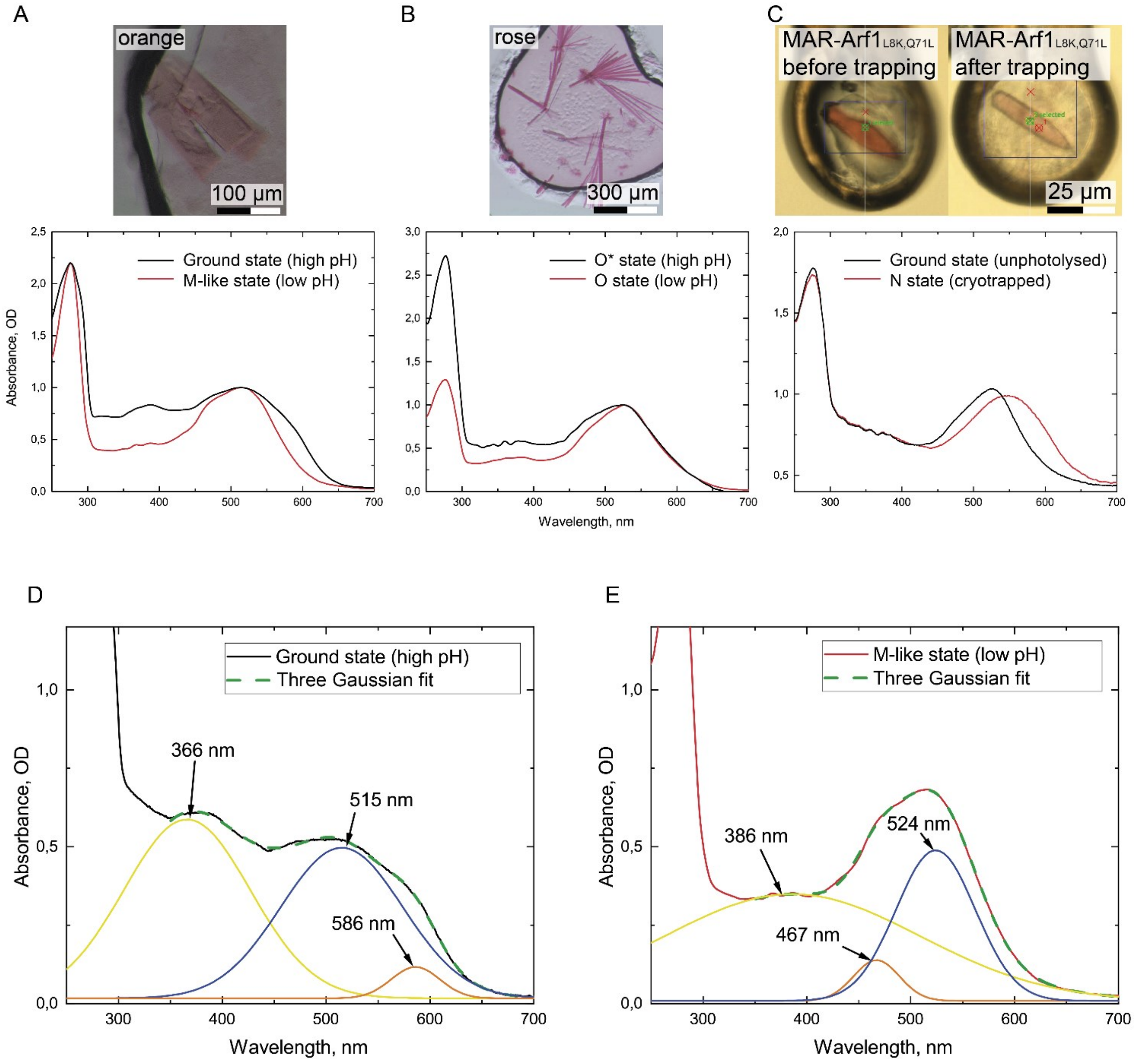

Examples of obtained MAR crystals and their UV-Vis spectra. (A), MAR orange-form crystals at high and low pH. **(B)**, MAR rose-form crystals at high and low pH. **(C)**, MAR-Arf1$_{L8K,Q71L}$ crystals before and after cryotrapping with a 532 nm-wavelength laser. **(D)**, a three Gaussian fit of the ground state crystals of MAR. **(E)**, a three Gaussian fit of the M-like state crystals of MAR. The three Gaussian fit is necessary to distinguish the MAR peak from the LCP background scattering and possible free retinal. The good agreement with the experimental curve confirms the reasonableness of this fit.

fig. S8.

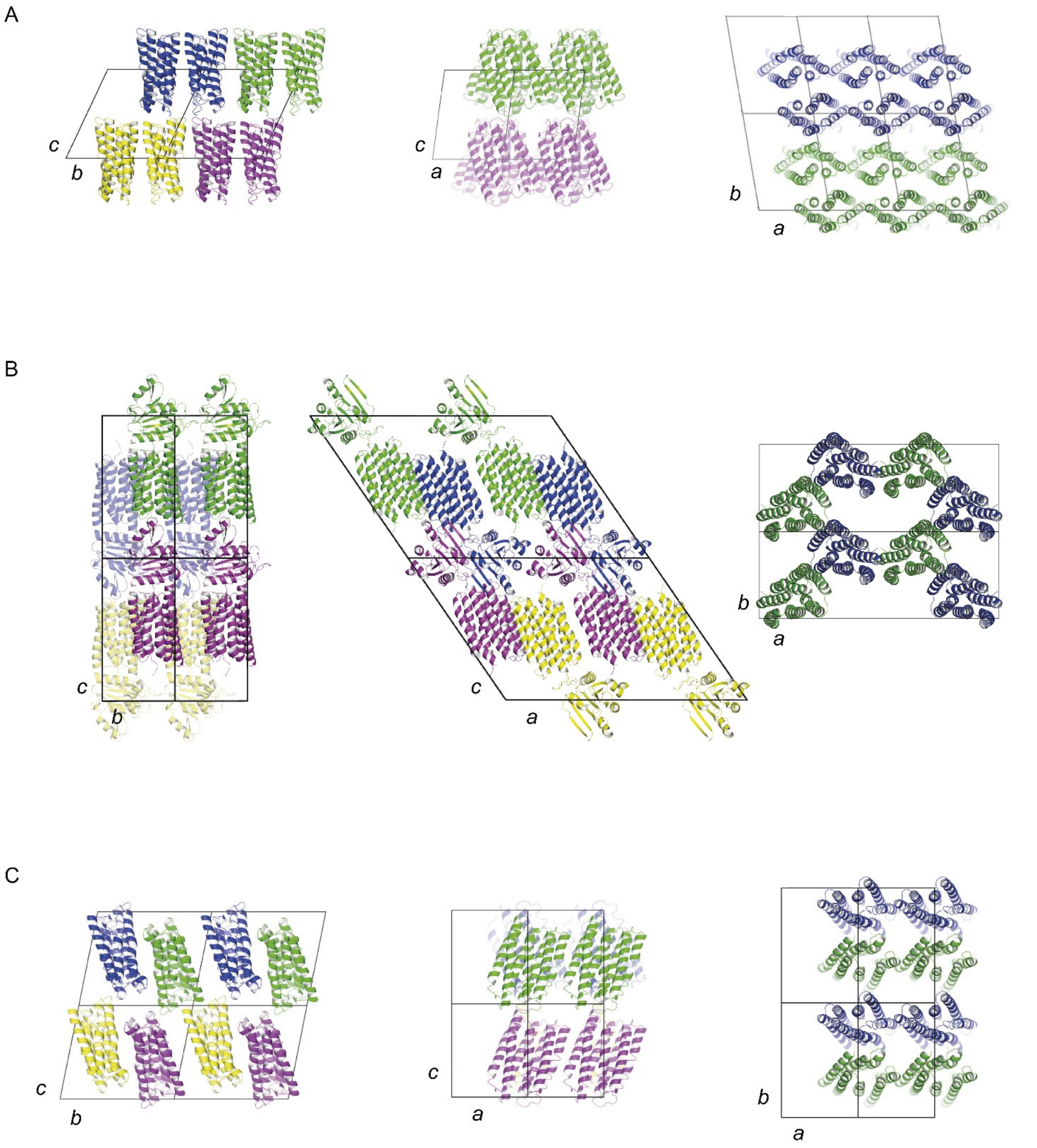

MAR packing in the crystals. (A), Orange-form crystals of MAR. **(B)**, MAR-Arf1$_{L8K,Q71L}$. **(C)**, Rose-form crystals of MAR.

fig. S9.

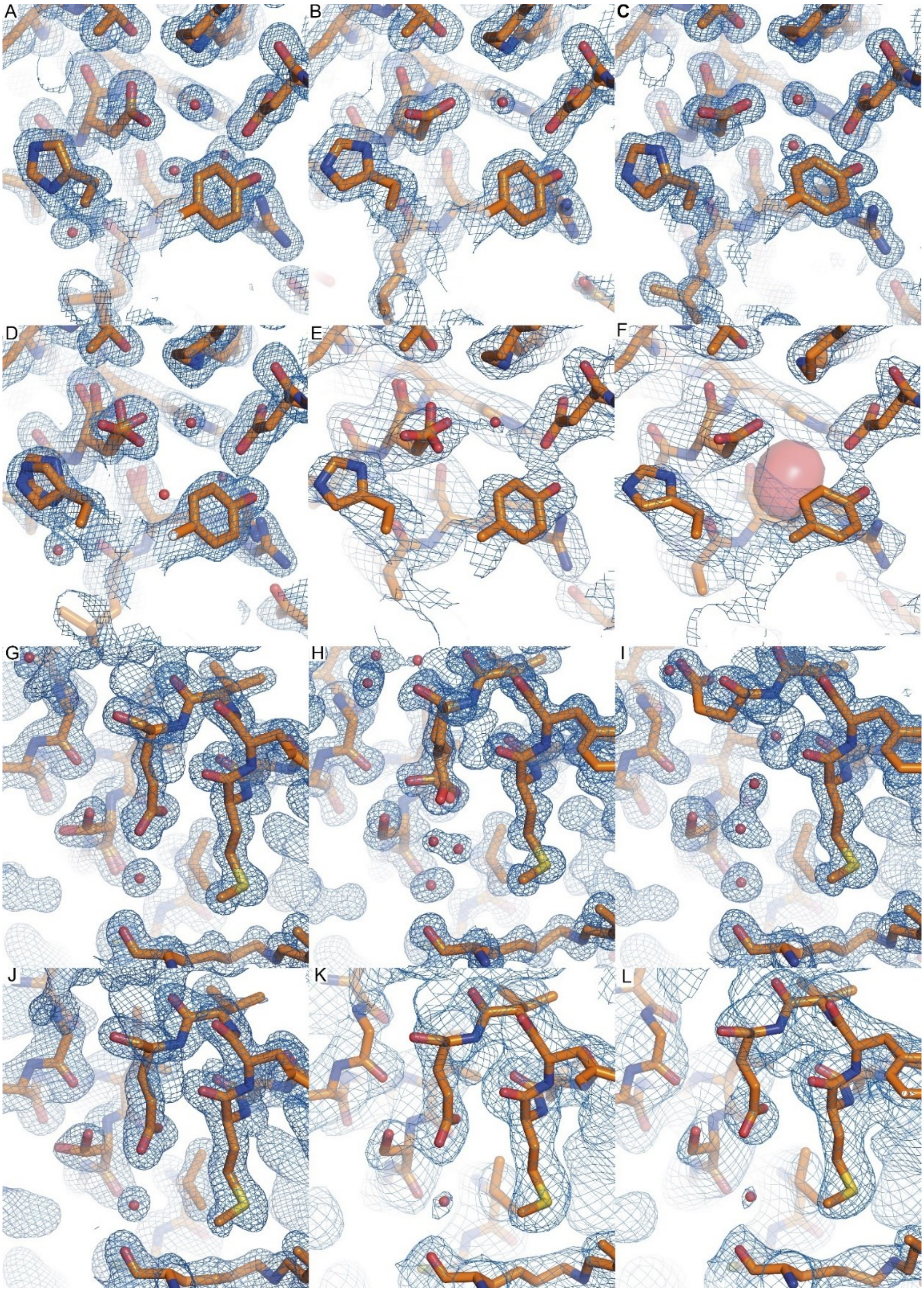

Examples of electron densities for different intermediate states of the MAR photocycle. (A) to **(F)**, RSB region in the structures of the ground state, M-like state, O state, P593 state, MAR-Arf1$_{L8K,Q71L}$ ground state, and MAR-Arf1$_{L8K,Q71L}$ N state, respectively. **(G)** to **(L)**, The proton uptake regions in the structures of the ground state, O state, O* state, P593 state, MAR-Arf1$_{L8K,Q71L}$ ground state, and MAR-Arf1$_{L8K,Q71L}$ N state, respectively. All electron density (2*m*Fo-*D*Fc; φcalc) maps are contoured at 1.2σ-level, except for the MAR-Arf1$_{L8K,Q71L}$ structures, for which 1.0σ-level was used. The cavity shown as a red surface in the panel (**F**) results from a calculation using HOLLOW (*114*).

fig. S10.

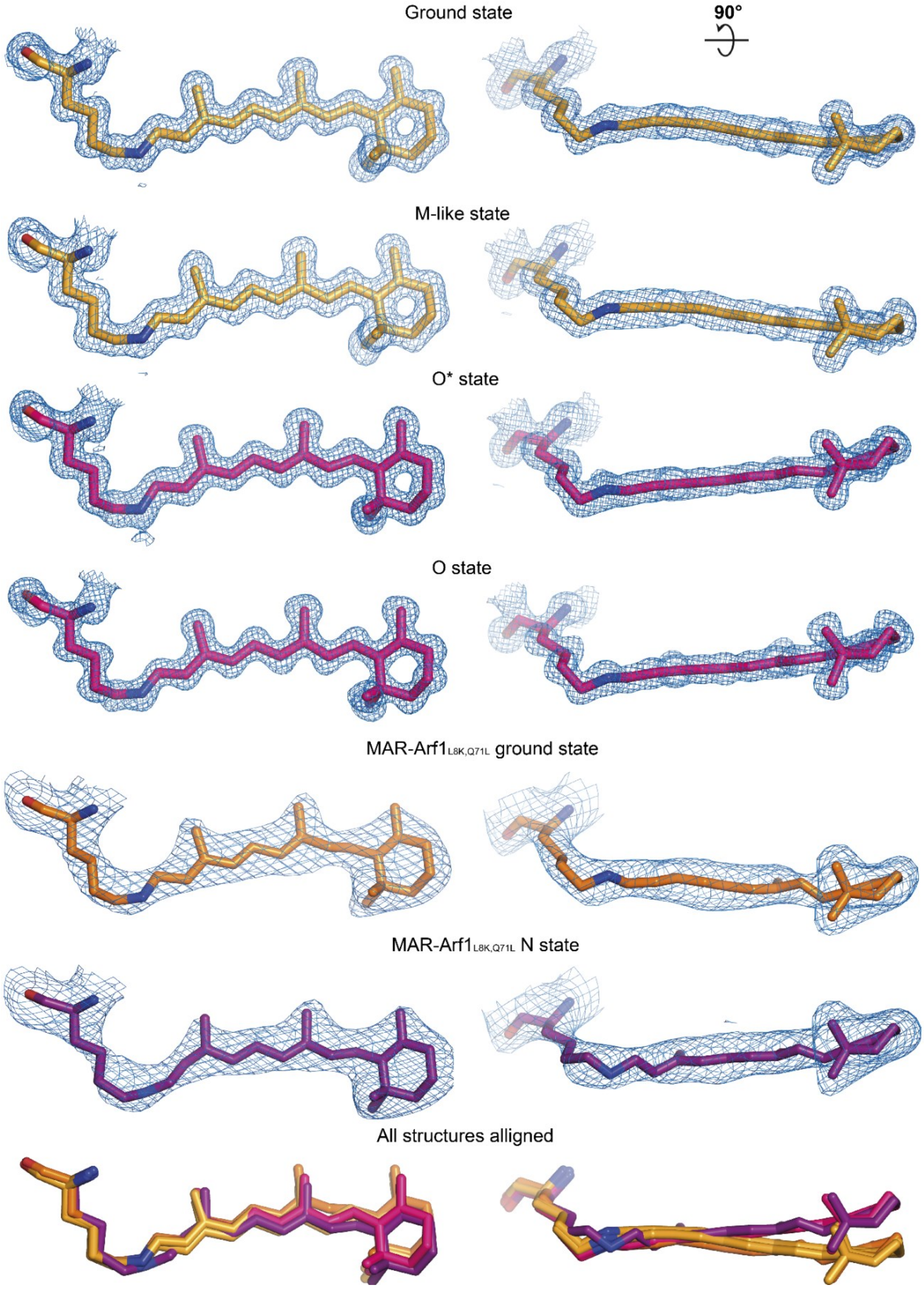

Orthogonal views of the electron density for Lys200 and the covalently bound retinal in the different intermediate states of MAR photocycle. All electron density (2*m*Fo-*D*Fc; φcalc) maps are contoured at 1.2σ-level, except for MAR-Arf1$_{L8K,Q71L}$ ground, and N states, for which 1.0σ-level was used. As can be seen, the RSB clearly adopts two different all-trans conformations in the ground state and the O* and O states. The details are discussed in ref. (*51*).

fig. S11.

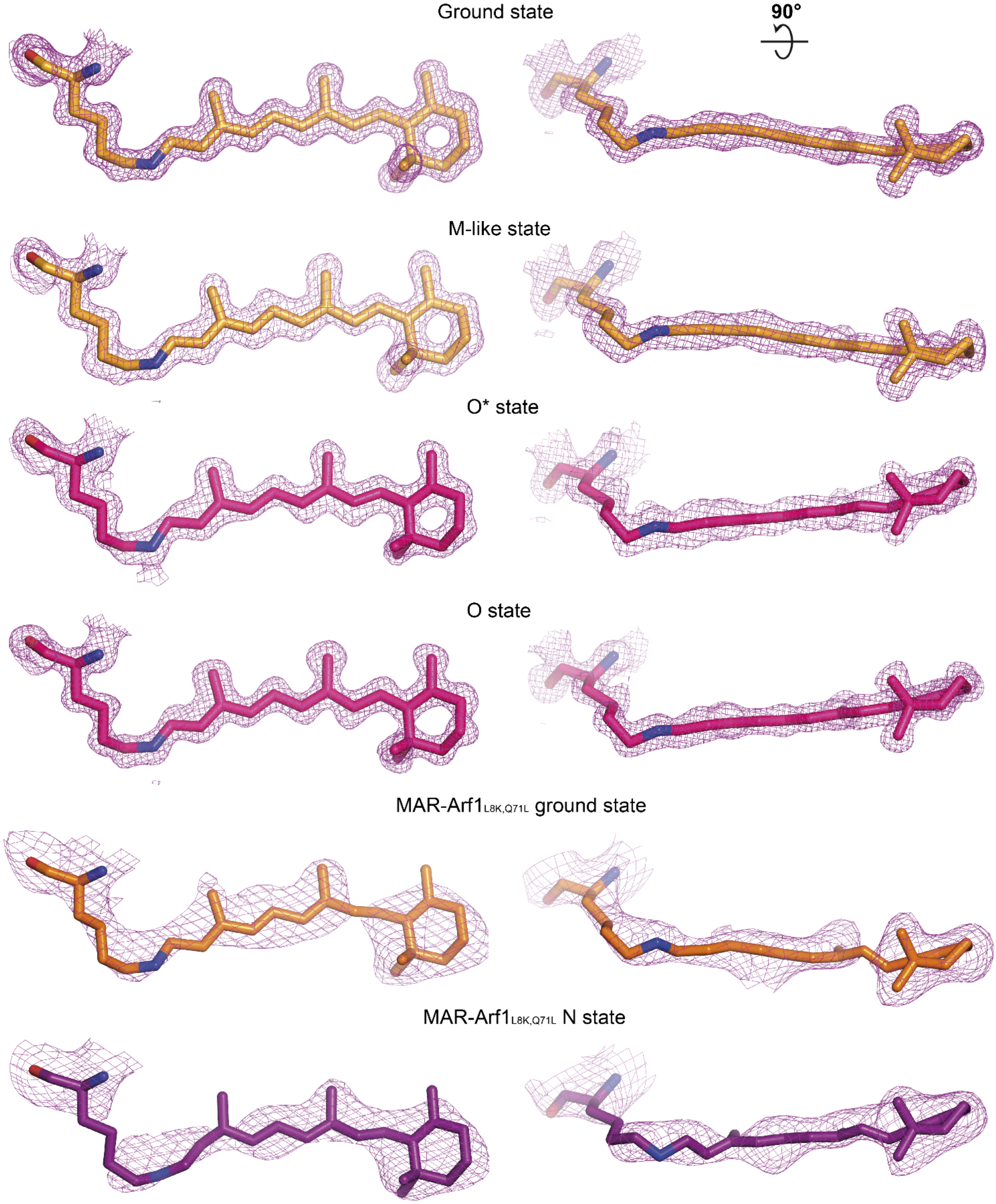

Orthogonal views of the 2*m*Fo-*D*Fc omit electron density map (anneal protocol) for Lys200 and the covalently bound retinal in the different intermediate states of the MAR photocycle. All electron density maps are contoured at 1.1σ-level, except for MAR-Arf1$_{L8K,Q71L}$ ground, and N states, for which 1.0σ-level was used.

fig. S12.

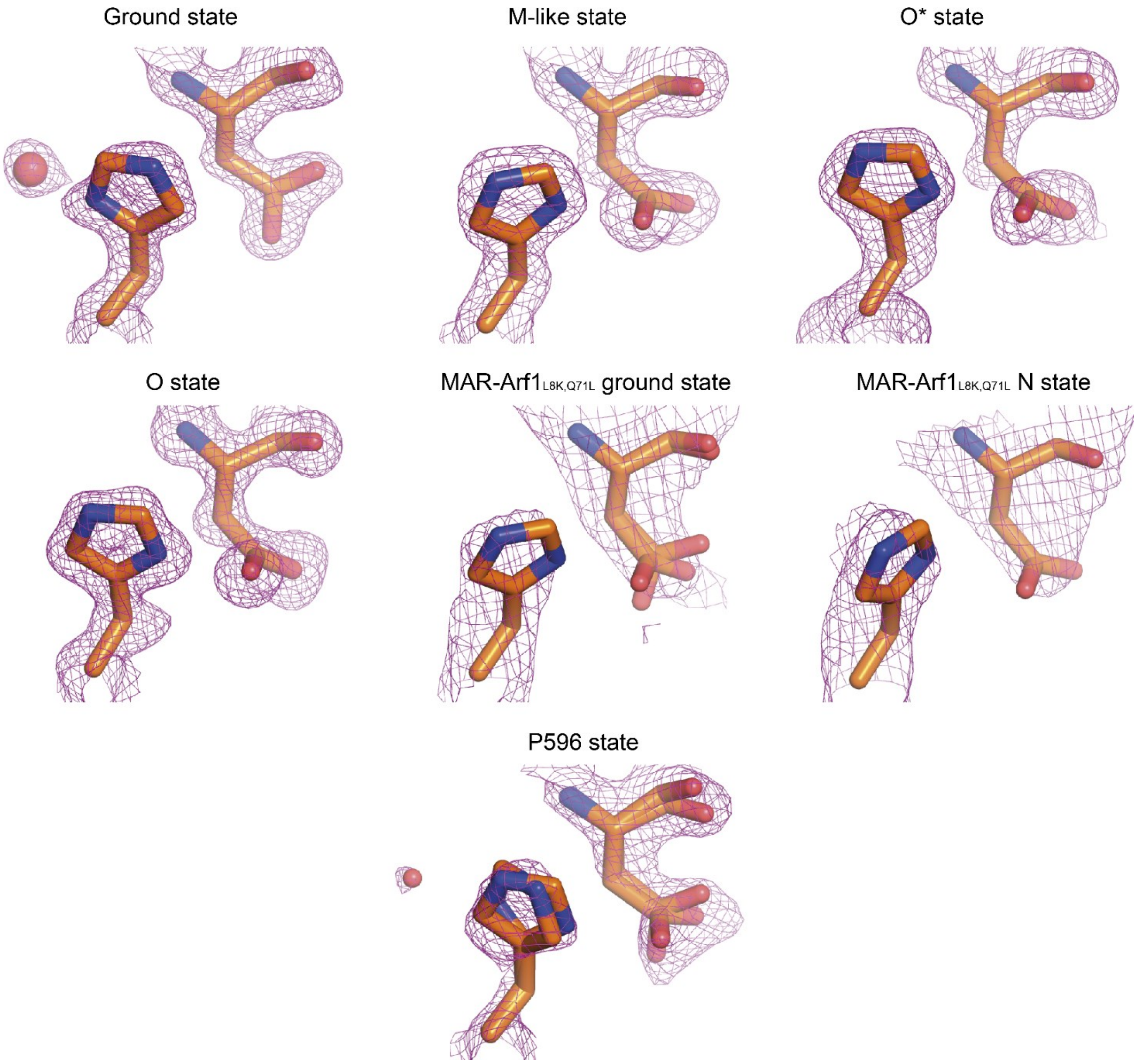

Views of the 2*m*Fo-*D*Fc omit electron density map (anneal protocol) for the His51-Asp72 pair in the different intermediate states of MAR photocycle. All electron density maps are contoured at 1.1σ-level, except for MAR-Arf1$_{L8K,Q71L}$ ground and N states, for which 1.0σ-level was used.

fig. S13.

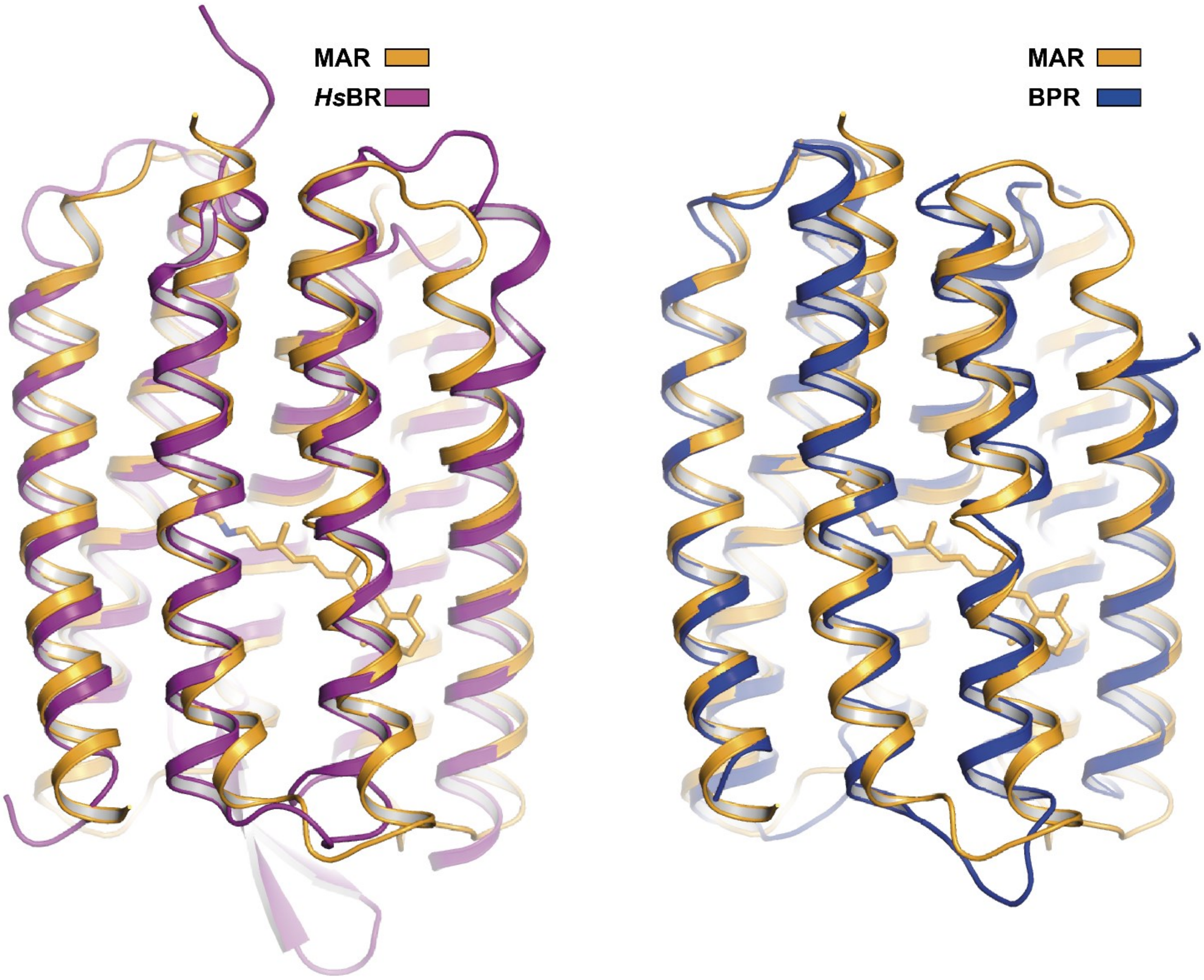

The overall fold of MAR. Comparison of the ground state structure of MAR with *Hs*BR [PDB ID: 7Z0A (*4*)] and BPR [PDB ID: 4JQ6 (*34*), chain B].

fig. S14.

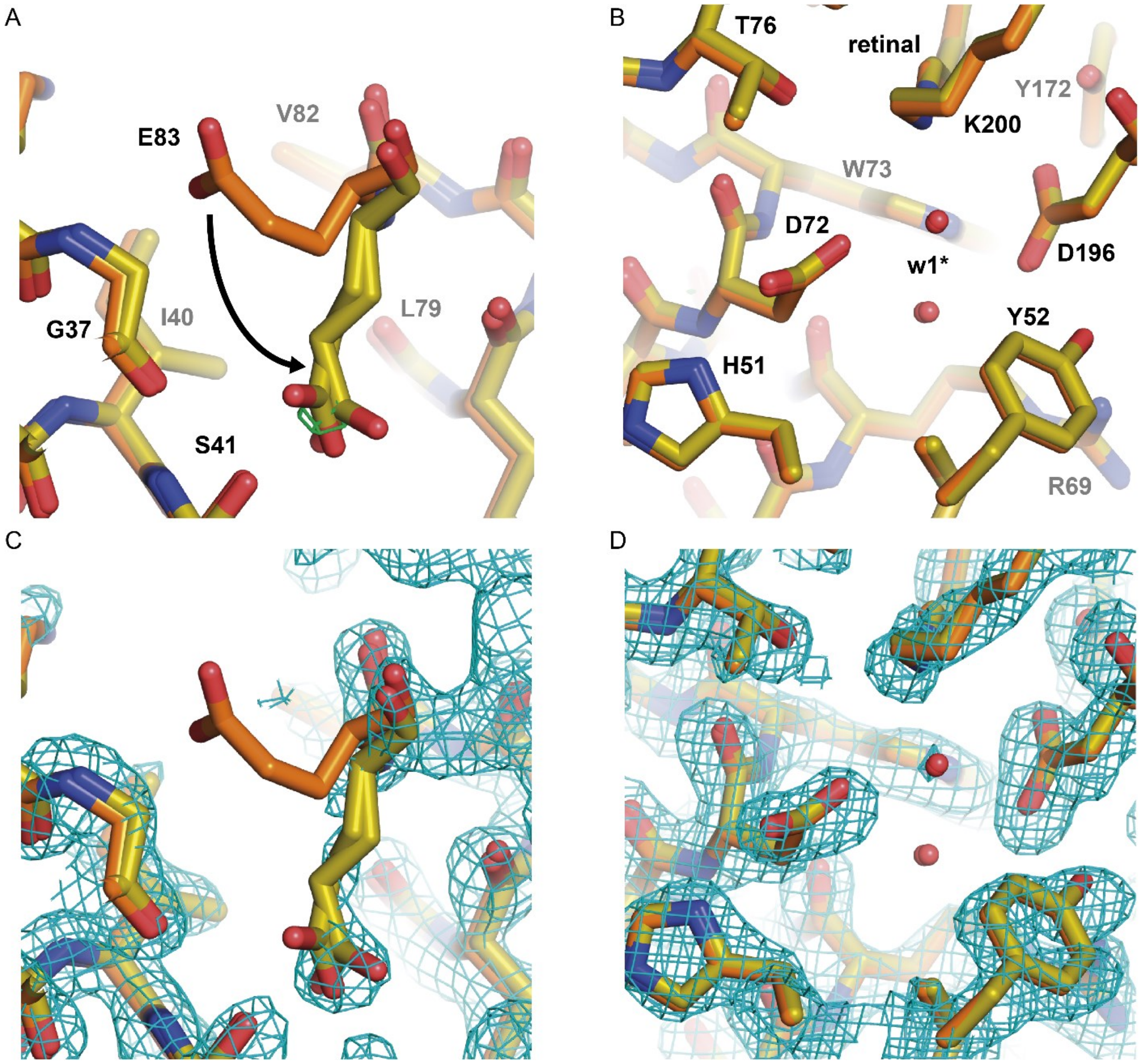

Cryotrapping of the O* state crystals of MAR. **(A)** and **(B)**, proton uptake region and RSB region, respectively. Structures of the O* and O states are aligned and colored orange and olive, respectively. F_O-F_O difference map is contoured at 3.5σ-level. The arrow shows the direction of the Glu83 flip. Two alternative Glu83 conformations are observed in the O state. No substantial densities are observed in the RSB region. The occupancy of changes is low [estimated as 20% by Xtrapol8 (*116*)]. This is because two states, O* and O, have similar absorption spectra, which does not allow the efficient accumulation of the O state upon illuminating the O* state. To prove the O state's accumulation, we also show extrapolated maps for the two regions **(C)** and **(D)** built with Xtrapol8 (*116*), colored cyan, and contoured at 1.5σ-level.

fig. S15.

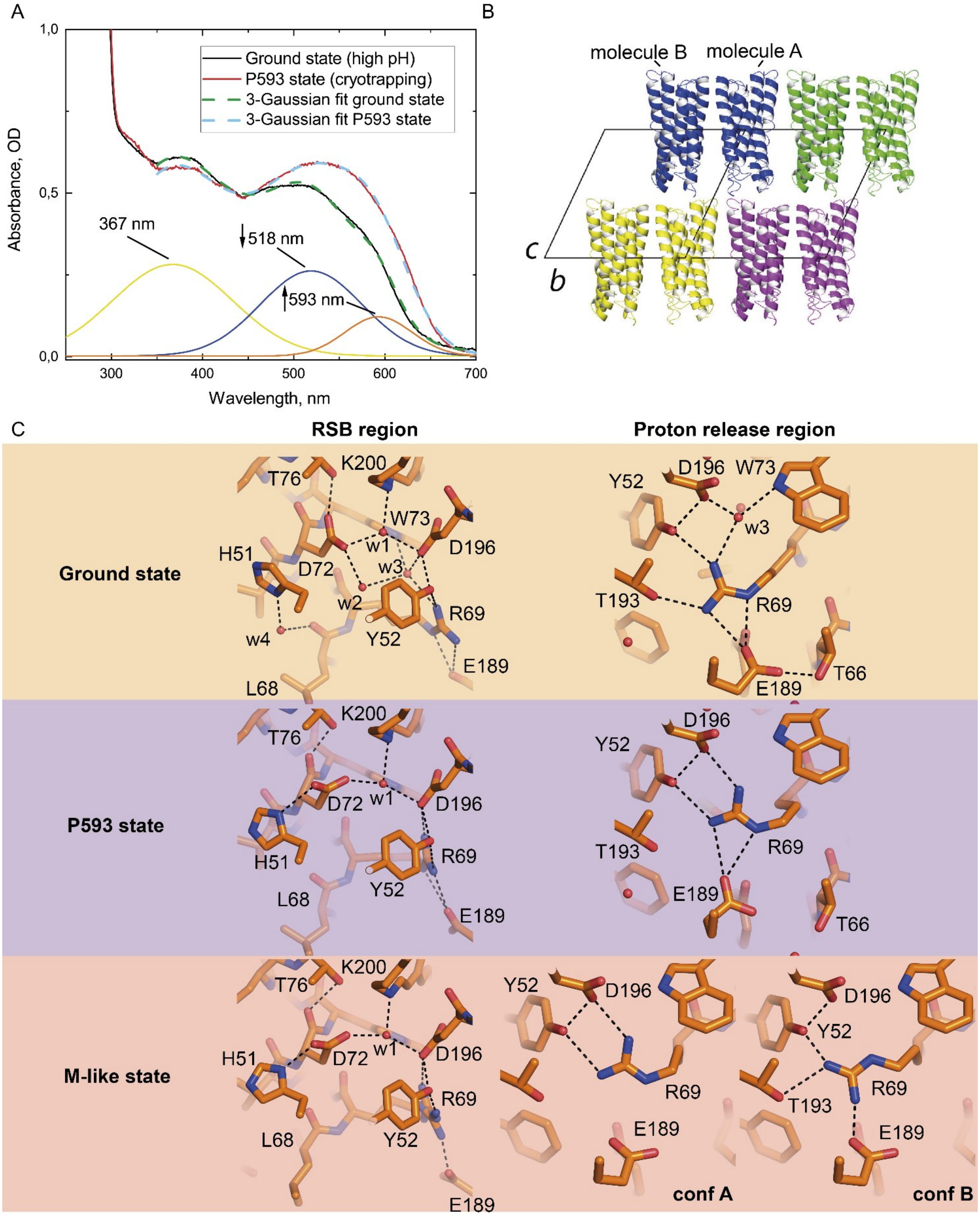

Crystal packing limits the functionality of MAR in the orange-form crystals. (A), Cryotrapping of MAR orange-form crystals. Spectrum is shown before (colored black) and after (colored red) laser activation. The three Gaussian global fit for the two spectra is shown with the dashed line. **(B)**, Packing of MAR orange form crystals. ASU comprises two non-equal protomers. **(C)**, Comparison of the RSB and proton release regions between 3 states, observed in the orange form MAR crystals. Polar contacts are shown with the dashed lines.

fig. S16.

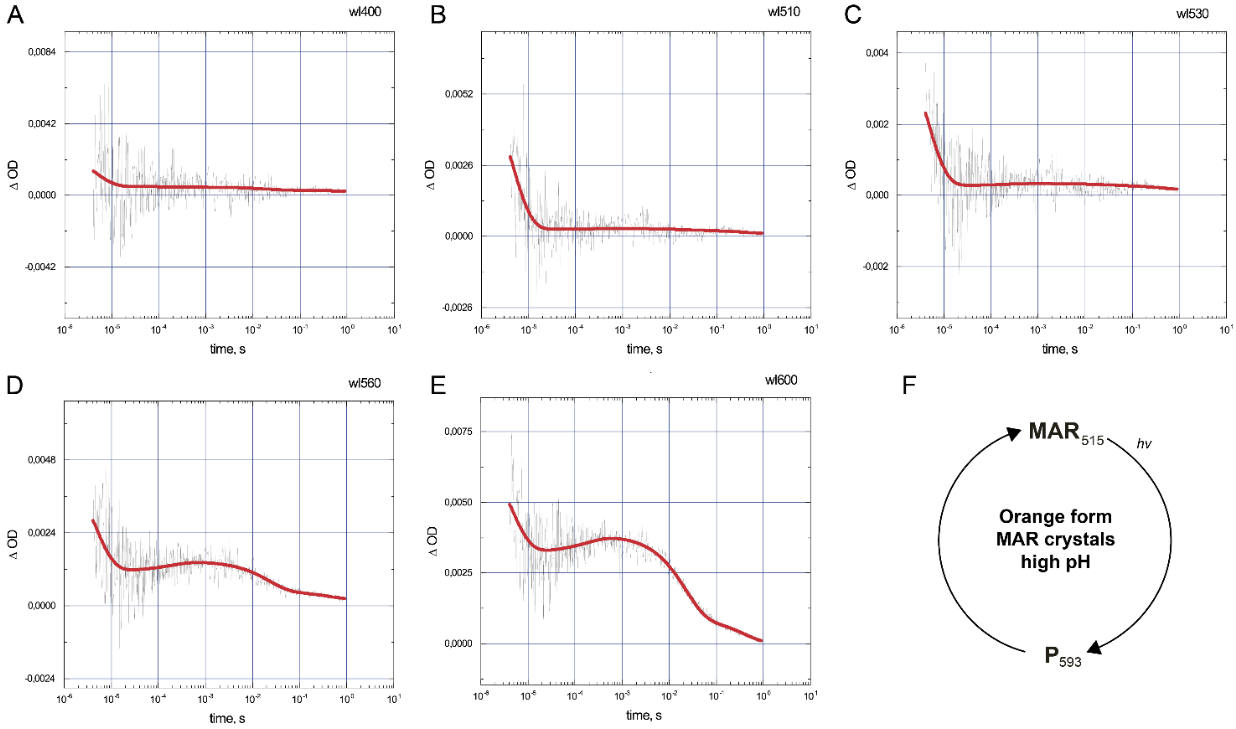

Photocycle of the MAR crystals in the orange form at high pH. (A) to **(E)**, The transient absorption changes of MAR crystals in the orange form at high pH at five characteristic wavelengths: 400 nm, 510 nm, 530 nm, 560 nm, and 600 nm. Black lines are the experimental data, while red lines represent the result of global fit using four exponents. **(F)**, Given the fitting results, a simplified version of the MAR photocycle in the orange form crystals at high pH.

fig. S17.

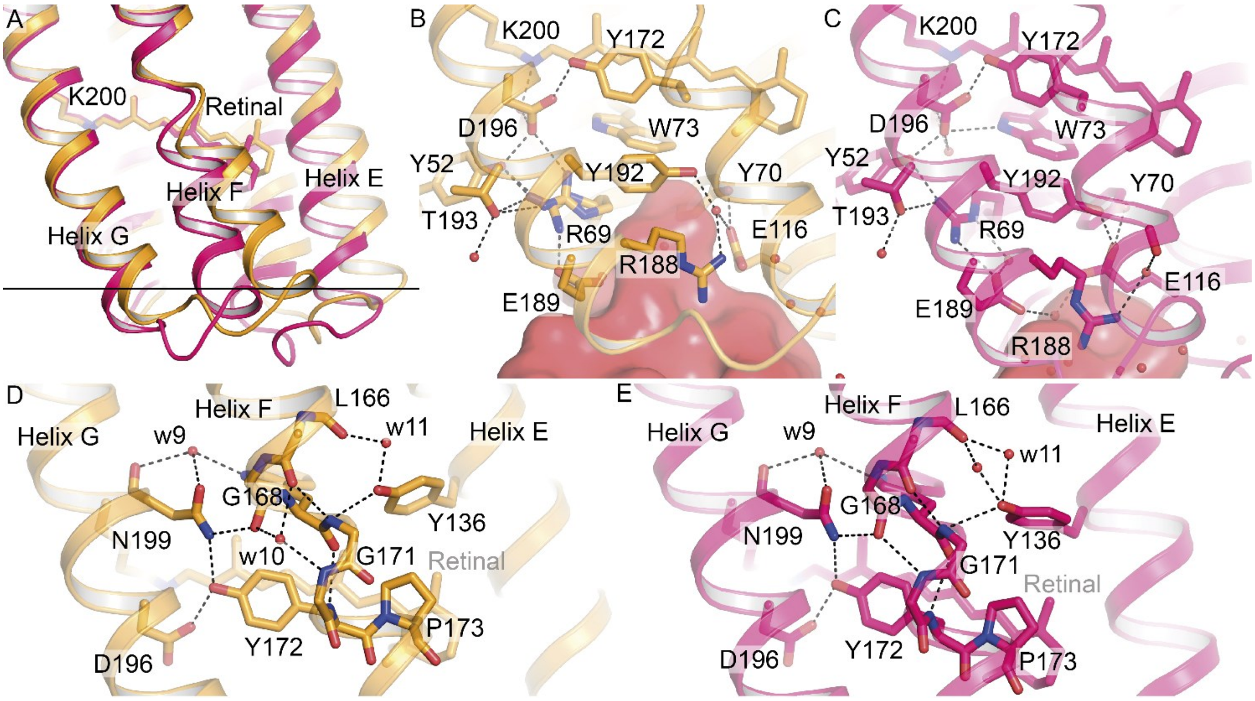

The role of helix F for controlling the solvent accessibility to the extracellular compartment of MAR. (A), Alignment of the proton release regions of MAR in the orange [colored orange, **(B)**] and the rose [colored rose, **(C)**] crystal forms. Helix F hinges in the area of Pro173. Cavities were calculated with HOLLOW (*114*). Structure of the helix F break in the orange (**D**) and rose (**E**) crystal forms of MAR. Putative H-bonds are shown with dashed lines. In the rose form structure, water molecule w10 is absent, and water molecule w11 occupies two alternative positions.

fig. S18.

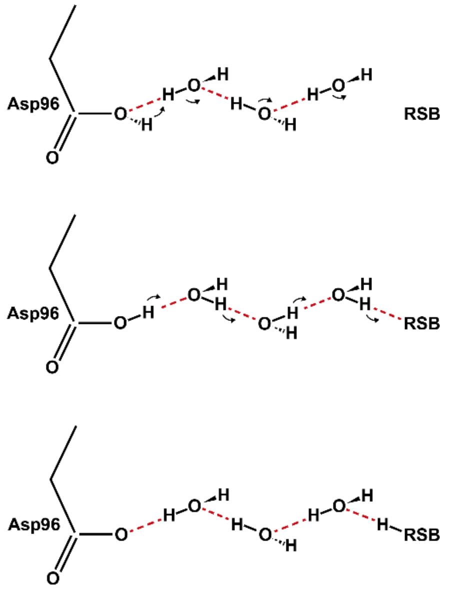

Hypothetical hop/turn mechanism of the RSB reprotonation during the M to N state transition in *Hs*BR. Red dashed lines depict HBCs. Black solid arrows indicate the hydrogen transfer steps.

fig. S19.

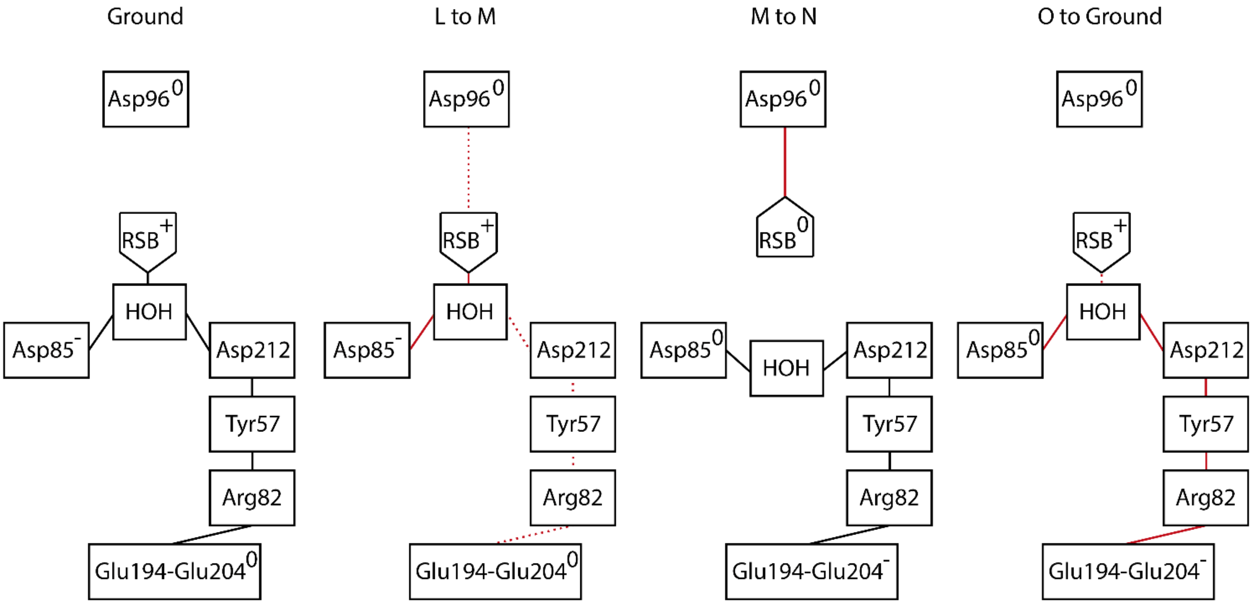

Schematic representation of the proton pumping in *Hs*BR. HBCs are shown with lines. HBCs that are involved in signal (dotted line) or proton (solid line) transfer on the particular step of proton pumping are colored red. RSB accessibility switch is depicted during the M to N state transition step.

table S1.

pH	$\Delta H_1^‡$	$\Delta S_1^‡$	$\Delta H_2^‡$	$\Delta S_2^‡$	$\Delta H_3^‡$	$\Delta S_3^‡$	$\Delta H_4^‡$	$\Delta S_4^‡$	$\Delta H_5^‡$	$\Delta S_5^‡$
5.0	37 ± 3	-18 ± 10	43 ± 1	-24 ± 4	60 ± 2	10 ± 5	51 ± 1	-45 ± 4	50 ± 1	-56 ± 4
7.5	60 ± 3	56 ± 9	38 ± 9	-42 ± 29	58± 3	3 ± 10	46 ± 2	-59 ± 5	37 ± 5	-97 ± 17
10.0	47 ± 2	16 ± 6	40 ± 2	-38 ± 6	56 ± 2	-4 ± 6	37 ± 3	-90 ± 10	46 ± 4	-73 ± 12

Activation barriers of five apparent rate constants for the MAR photocycle at different pH. Enthalpies ($\Delta H^‡$, *kJ/mol*) and entropies ($\Delta S^‡$, *J/mol K*) of activation were derived according to the Transition State Theory from non-linear fitting of data using equation $Ln(k_i) = Ln\left(\frac{k_B T}{h}\right) + \frac{\Delta S_i^‡}{R} - \frac{\Delta H_i^‡}{RT}$, where $k_{i=1..5}$ are experimental rate constants, and k_B, h, R, T are Boltzmann, Planck, gas constants and Kelvin temperature, respectively.

table S2.

Name	MAR ground state	MAR M-like state	MAR P593 state	MAR-Arf1 mutant ground state	MAR-Arf1 mutant N state	MAR O* state	MAR O state
pH	8.8	5.2	8.8	7.0	7.0	8.4	4.6
UV-Vis absorption peak, nm	515	524	593	525	550	530	530
Beamline	ESRF ID23–1	ESRF ID23–1	EMBL Hamburg P14	ESRF ID30B	ESRF ID30A–3	ESRF ID23–1	ESRF ID23–1
Wavelength, Å	0.9537	0.9724	0.9755	0.9763	0.9677	0.8856	0.9724
Space group	P1	P1	P1	C2	C2	P2	P2
Unit cell							
a, Å	40.71	40.69	40.68	132.87	133.19	50.61	50.63
b, Å	55.88	56.63	55.98	39.72	39.79	40.17	40.42
c, Å	56.61	57.39	56.73	94.98	95.76	60.31	60.43
α, °	64.19	63.66	64.41	90.00	90.00	90.00	90.00
β, °	81.81	78.98	81.31	124.63	124.54	101.22	101.37
γ, °	85.47	80.33	84.43	90.00	90.00	90.00	90.00
Resolution, Å	47.23 – 1.24 (1.36 – 1.24)	50.98 – 1.60 (1.63 – 1.60)	40.19 – 1.41 (1.54 – 1.41)	37.33 – 2.30 (2.41 – 2.30)	39.44 – 2.10 (2.19 – 2.10)	40.17 – 1.41 (1.55 – 1.41)	59.24 – 1.08 (1.18 – 1.08)
Anisotropic diffraction limits, Å	1.28, 1.42, 1.24	-	1.38, 1.82, 1.48	2.40, 2.10, 2.50	2.06, 1.96, 2.53	1.44, 1.45, 2.15	1.06, 1.17, 1.32
Total number of observations	337772 (11683)	641326 (31260)	200825 (10435)	34487 (1446)	58647 (2829)	68063 (2358)	941677 (44506)
Total number unique	92902 (4644)	59048 (2944)	52344 (2662)	15145 (759)	19820 (992)	27492 (1375)	71975 (3599)
<(I)/σ(I)>	9.6 (1.3)	23.2 (2.8)	11.5 (0.7)	8.0 (1.6)	6.4 (0.6)	7.0 (1.3)	13.7 (1.9)
CC(1/2)	0.996 (0.352)	1.000 (0.772)	0.999 (0.285)	0.998 (0.702)	0.997 (0.172)	0.998 (0.709)	0.999 (0.747)
Rpim	0.037 (0.670)	0.017 (0.284)	0.029 (1.069)	0.052 (0.409)	0.068 (1.279)	0.047 (0.344)	0.024 (0.364)
Completeness (spherical), %	73.8 (15.3)	99.7 (99.7)	60.5 (13.3)	81.8 (33.2)	80.9 (34.5)	60.2 (12.5)	69.1 (14.2)
Completeness (ellipsoidal), %	86.6 (35.5)	N/A	79.5 (45.0)	88.9 (69.7)	93.0 (71.5)	88.8 (46.5)	89.9 (56.8)
Multiplicity	3.6 (2.5)	10.9 (10.6)	3.8 (3.9)	2.3 (1.9)	3.0 (2.9)	2.5 (1.7)	13.1 (12.4)

Data collection statistics for the crystal structures of MAR intermediate states. Numbers in parentheses are for the high-resolution shells.

table S3.

Name	MAR ground state	MAR M-like state	MAR P593 state	MAR-Arf1 mutant ground state	MAR-Arf1 mutant N state	MAR O* state	MAR O state
PDB ID	8RSO	7AVN	8RSP	8RSQ	8RSR	8RSS	7AVP
Resolution range, Å	33.60 – 1.25	50.57 – 1.60	40.19 – 1.41	36.56 – 2.30	39.44 – 2.30	29.58 – 1.41	42.38 – 1.09
Reflections used in refinement/for calculation of R-free	92890/4304	56027/3019	51309/2406	15122/1534	17205/1730	27485/1347	69035/3381
R-work/R-free	0.1517/0.1768	0.1754/0.2044	0.2323/0.2618	0.2108/0.2636	0.2525/0.2822	0.1595/0.1978	0.1338/0.1527
Number of non-hydrogen atoms	3969	4023	3857	3281	3143	2224	2161
macromolecules	3506	3490	3503	3082	2963	1910	1753
retinal	40	40	40	20	20	20	20
lipids	280	335	254	41	25	137	179
solvent	143	158	60	110	107	157	209
Protein residues	433	434	433	389	388	218	217
RMS(bonds), Å	0.010	0.014	0.006	0.001	0.002	0.003	0.008
RMS(angles), °	1.12	1.61	0.78	0.46	0.47	0.59	1.00
Ramachandran favored, %	99.76	99.29	99.53	98.95	98.69	99.53	99.53
Ramachandran allowed, %	0.24	0.71	0.47	1.05	1.31	0.47	0.47
Ramachandran outliers, %	0.00	0.00	0.00	0.00	0.00	0.00	0.00
Rotamer outliers, %	1.47	3.16	1.76	1.00	0.35	1.04	0.56
Clashscore	5.50	12.85	6.08	3.98	3.36	4.00	2.20
Average B-factor, Å^2	25.57	30.94	33.32	49.06	61.45	20.08	20.14
macromolecules	23.65	28.87	32.63	49.46	61.88	17.94	14.30
retinal	16.46	17.08	23.39	35.08	50.36	10.22	8.65
lipids	43.97	48.58	43.36	52.81	63.35	36.00	59.13
solvent	39.24	42.78	38.10	42.03	55.27	33.41	36.77
Cruickshank DPI, Å	0.0568	0.0888	0.1385	1.3916	0.5644	0.0974	0.0391
H-bond length error estimation, Å	0.1	0.2	0.3	0.3	0.3	0.2	0.1

Refinement statistics for the crystal structures of MAR intermediate states.

table S4.

Name	O* state reference	O state cryotrapped
Crystallization conditions	2.1 M AmPi, pH 8.8	2.1 M AmPi, pH 8.8
Beamline	ESRF ID30A–3	ESRF ID30A–3
Wavelength, Å	0.9677	0.9677
Space group	P2	P2
Unit cell		
a, Å	50.69	50.54
b, Å	40.27	40.31
c, Å	60.25	60.30
α, °	90	90
β, °	100.57	101.25
γ, °	90	90
Resolution, Å	59.23 – 2.05<br>(2.22 – 2.05)	49.57 – 1.52<br>(1.63 – 1.52)
Total number of observations	33039 (1689)	114066 (4644)
Total number unique	10521 (527)	29957 (1498)
<(I)/σ(I)>	4.5 (1.5)	10.0 (1.4)
CC(1/2)	0.987 (0.644)	0.998 (0.533)
Rpim	0.095 (0.377)	0.046 (0.501)
Completeness (spherical), %	69.3 (17.1)	80.2 (20.2)
Completeness (ellipsoidal), %	85.2 (45.0)	91.0 (44.3)
Multiplicity	3.1 (3.2)	3.8 (3.1)

Data collection statistics for the cryotrapping experiment with the MAR O* state crystals. Numbers in parentheses are for the high-resolution shells.

table S5.

Name	O* state reference	O state cryotrapped
PDB ID	9G15	9G16
Resolution range, Å	34.93 – 2.05	49.57 – 1.52
Reflections used in refinement/for calculation of R-free	10279/1020	29951/1487
R-work/R-free	0.2195/0.2655	0.1760/0.2089
Number of non-hydrogen atoms	2178	2235
macromolecules	1901	1923
retinal	20	20
lipids	137	137
solvent	120	155
Protein residues	218	218
RMS(bonds), Å	0.002	0.006
RMS(angles), °	0.51	0.78
Ramachandran favored, %	99.53	100.00
Ramachandran allowed, %	0.47	0.00
Ramachandran outliers, %	0.00	0.00
Rotamer outliers, %	1.05	1.55
Clashscore	4.25	2.34
Average B-factor, $Å^2$	22.77	20.75
macromolecules	21.89	18.35
retinal	16.02	12.00
lipids	30.31	41.42
solvent	29.31	33.24

Refinement statistics for the cryotrapping experiment with the MAR O* state crystals.

Caption for movie S1.

Proton accessibility of MAR switches between extracellular (ground state) and cytoplasmic (O state) sides.

Caption for data S1.

Plasmids that were used for the expression of MAR and MAR-Arf1$_{L8K,Q71L}$.

REFERENCES AND NOTES

1. O. Béja, E. N. Spudich, J. L. Spudich, M. Leclerc, E. F. DeLong, Proteorhodopsin phototrophy in the ocean. *Nature* **411**, 786–789 (2001).

2. L. Gómez-Consarnau, J. A. Raven, N. M. Levine, L. S. Cutter, D. Wang, B. Seegers, J. Arístegui, J. A. Fuhrman, J. M. Gasol, S. A. Sañudo-Wilhelmy, Microbial rhodopsins are major contributors to the solar energy captured in the sea. *Sci. Adv.* **5**, eaaw8855 (2019).

3. V. Borshchevskiy, K. Kovalev, E. Round, R. Efremov, R. Astashkin, G. Bourenkov, D. Bratanov, T. Balandin, I. Chizhov, C. Baeken, I. Gushchin, A. Kuzmin, A. Alekseev, A. Rogachev, D. Willbold, M. Engelhard, E. Bamberg, G. Büldt, V. Gordeliy, True-atomic-resolution insights into the structure and functional role of linear chains and low-barrier hydrogen bonds in proteins. *Nat. Struct. Mol. Biol.* **29**, 440–450 (2022).

4. D. Zabelskii, N. Dmitrieva, O. Volkov, V. Shevchenko, K. Kovalev, T. Balandin, D. Soloviov, R. Astashkin, E. Zinovev, A. Alekseev, E. Round, V. Polovinkin, I. Chizhov, A. Rogachev, I. Okhrimenko, V. Borshchevskiy, V. Chupin, G. Büldt, N. Yutin, E. Bamberg, E. Koonin, V. Gordeliy, Structure-based insights into evolution of rhodopsins. *Commun. Biol.* **4**, 821 (2021).

5. T. Friedrich, S. Geibel, R. Kalmbach, I. Chizhov, K. Ataka, J. Heberle, M. Engelhard, E. Bamberg, Proteorhodopsin is a light-driven proton pump with variable vectoriality. *J. Mol. Biol.* **321**, 821–838 (2002).

6. L. Zimányi, G. Váró, M. Chang, B. Ni, R. Needleman, J. K. Lanyi, Pathways of proton release in the bacteriorhodopsin photocycle. *Biochemistry* **31**, 8535–8543 (1992).

7. I. Gushchin, P. Chervakov, P. Kuzmichev, A. N. Popov, E. Round, V. Borshchevskiy, A. Ishchenko, L. Petrovskaya, V. Chupin, D. A. Dolgikh, A. A. Arseniev, M. Kirpichnikov, V. Gordeliy, Structural insights into the proton pumping by unusual proteorhodopsin from nonmarine bacteria. *Proc. Natl. Acad. Sci. U.S.A.* **110**, 12631–12636 (2013).

8. V. B. Bergo, O. A. Sineshchekov, J. M. Kralj, R. Partha, E. N. Spudich, K. J. Rothschild, J. L. Spudich, His-75 in proteorhodopsin, a novel component in light-driven proton translocation by

primary pumps. *J. Biol. Chem.* **284**, 2836–2843 (2009).

9. F. Hempelmann, S. Hölper, M.-K. Verhoefen, A. C. Woerner, T. Köhler, S.-A. Fiedler, N. Pfleger, J. Wachtveitl, C. Glaubitz, His75-Asp97 cluster in green proteorhodopsin. *J. Am. Chem. Soc.* **133**, 4645–4654 (2011).

10. R. Astashkin, K. Kovalev, S. Bukhdruker, S. Vaganova, A. Kuzmin, A. Alekseev, T. Balandin, D. Zabelskii, I. Gushchin, A. Royant, D. Volkov, G. Bourenkov, E. Koonin, M. Engelhard, E. Bamberg, V. Gordeliy, Structural insights into light-driven anion pumping in cyanobacteria. *Nat. Commun.* **13**, 6460 (2022).

11. K. Kovalev, R. Astashkin, I. Gushchin, P. Orekhov, D. Volkov, E. Zinovev, E. Marin, M. Rulev, A. Alekseev, A. Royant, P. Carpentier, S. Vaganova, D. Zabelskii, C. Baeken, I. Sergeev, T. Balandin, G. Bourenkov, X. Carpena, R. Boer, N. Maliar, V. Borshchevskiy, G. Büldt, E. Bamberg, V. Gordeliy, Molecular mechanism of light-driven sodium pumping. *Nat. Commun.* **11**, 2137 (2020).

12. L. S. Brown, Light-driven proton transfers and proton transport by microbial rhodopsins – A biophysical perspective. *Biochim. Biophys. Acta Biomembr.* **1864**, 183867 (2022).

13. R. Efremov, V. I. Gordeliy, J. Heberle, G. Büldt, Time-resolved microspectroscopy on a single crystal of bacteriorhodopsin reveals lattice-induced differences in the photocycle kinetics. *Biophys. J.* **91**, 1441–1451 (2006).

14. S. Engilberge, N. Caramello, S. Bukhdruker, M. Byrdin, T. Giraud, P. Jacquet, D. Scortani, R. Biv, H. Gonzalez, A. Broquet, P. van der Linden, S. L. Rose, D. Flot, T. Balandin, V. Gordeliy, J. M. Lahey-Rudolph, M. Roessle, D. de Sanctis, G. A. Leonard, C. Mueller-Dieckmann, A. Royant, The TR-icOS setup at the ESRF: Time-resolved microsecond UV-Vis absorption spectroscopy on protein crystals. *Acta Crystallogr. D Struct. Biol.* **80**, 16–25 (2024).

15. T. Weinert, P. Skopintsev, D. James, F. Dworkowski, E. Panepucci, D. Kekilli, A. Furrer, S. Brünle, S. Mous, D. Ozerov, P. Nogly, M. Wang, J. Standfuss, Proton uptake mechanism in bacteriorhodopsin captured by serial synchrotron crystallography. *Science* **365**, 61–65 (2019).

16. R. Ghai, C. M. Mizuno, A. Picazo, A. Camacho, F. Rodriguez-Valera, Metagenomics uncovers a new group of low GC and ultra-small marine Actinobacteria. *Sci. Rep.* **3**, 2471 (2013).

17. E. M. Landau, J. P. Rosenbusch, Lipidic cubic phases: A novel concept for the crystallization of membrane proteins. *Proc. Natl. Acad. Sci. U.S.A.* **93**, 14532–14535 (1996).

18. H. Luecke, B. Schobert, H. T. Richter, J. P. Cartailler, J. K. Lanyi, Structural changes in bacteriorhodopsin during ion transport at 2 angstrom resolution. *Science* **286**, 255–260 (1999).

19. H. Luecke, H. T. Richter, J. K. Lanyi, Proton transfer pathways in bacteriorhodopsin at 2.3 angstrom resolution. *Science* **280**, 1934–1937 (1998).

20. K. Kovalev, F. Tsybrov, A. Alekseev, V. Shevchenko, D. Soloviov, S. Siletsky, G. Bourenkov, M. Agthe, M. Nikolova, D. von Stetten, R. Astashkin, S. Bukhdruker, I. Chizhov, A. Royant, A. Kuzmin, I. Gushchin, R. Rosselli, F. Rodriguez-Valera, N. Ilyinskiy, A. Rogachev, V. Borshchevskiy, T. R. Schneider, E. Bamberg, V. Gordeliy, Mechanisms of inward transmembrane proton translocation. *Nat. Struct. Mol. Biol.* **30**, 970–979 (2023).

21. S. Bukhdruker, I. Melnikov, C. Baeken, T. Balandin, V. Gordeliy, Crystallographic insights into lipid-membrane protein interactions in microbial rhodopsins. *Front. Mol. Biosci.* **11**, 1503709 (2024).

22. M. López-Pérez, J. M. Haro-Moreno, J. Iranzo, F. Rodriguez-Valera, Genomes of the " *Candidatus* Actinomarinales" order: Highly streamlined marine epipelagic actinobacteria. *mSystems* **5**, e01041-20 (2020).

23. J. Pinhassi, E. F. DeLong, O. Béjà, J. M. González, C. Pedrós-Alió, Marine bacterial and archaeal ion-pumping rhodopsins: Genetic diversity, physiology, and ecology. *Microbiol. Mol. Biol. Rev.* **80**, 929–954 (2016).

24. A. Higuchi, W. Shihoya, M. Konno, T. Ikuta, H. Kandori, K. Inoue, O. Nureki, Crystal structure of schizorhodopsin reveals mechanism of inward proton pumping. *Proc. Natl. Acad. Sci. U.S.A.* **118**, e2016328118 (2021).

25. T. Köhler, I. Weber, C. Glaubitz, J. Wachtveitl, Proteorhodopsin Photocycle Kinetics Between pH 5 and pH 9. *Photochem. Photobiol.* **93**, 762–771 (2017).

26. K. M. Stone, J. Voska, M. Kinnebrew, A. Pavlova, M. J. N. Junk, S. Han, Structural insight into proteorhodopsin oligomers. *Biophys. J.* **104**, 472–481 (2013).

27. S. Hirschi, D. Kalbermatter, Z. Ucurum, T. Lemmin, D. Fotiadis, Cryo-EM structure and dynamics of the green-light absorbing proteorhodopsin. *Nat. Commun.* **12**, 4107 (2021).

28. S. Hirschi, T. Lemmin, N. Ayoub, D. Kalbermatter, D. Pellegata, Z. Ucurum, J. Gertsch, D. Fotiadis, Structural insights into the mechanism and dynamics of proteorhodopsin biogenesis and retinal scavenging. *Nat. Commun.* **15**, 6950 (2024).

29. T. Morizumi, W. L. Ou, N. Van Eps, K. Inoue, H. Kandori, L. S. Brown, O. P. Ernst, X-ray crystallographic structure and oligomerization of Gloeobacter rhodopsin. *Sci. Rep.* **9**, 11283 (2019).

30. A. Iizuka, K. Kajimoto, T. Fujisawa, T. Tsukamoto, T. Aizawa, N. Kamo, K.-H. Jung, M. Unno, M. Demura, T. Kikukawa, Functional importance of the oligomer formation of the cyanobacterial H^+ pump Gloeobacter rhodopsin. *Sci. Rep.* **9**, 10711 (2019).

31. V. I. Gordeliy, J. Labahn, R. Moukhametzianov, R. Efremov, J. Granzin, R. Schlesinger, G. Büldt, T. Savopol, A. J. Scheidig, J. P. Klare, M. Engelhard, Molecular basis of transmembrane signalling by sensory rhodopsin II-transducer complex. *Nature* **419**, 484–487 (2002).

32. V. I. Gordeliy, R. Schlesinger, R. Efremov, G. Büldt, J. Heberle, Crystallization in lipidic cubic phases: A case study with bacteriorhodopsin. *Methods Mol. Biol.* **228**, 305–316 (2003).

33. H. Luecke, B. Schobert, J. Stagno, E. S. Imasheva, J. M. Wang, S. P. Balashov, J. K. Lanyi, Crystallographic structure of xanthorhodopsin, the light-driven proton pump with a dual chromophore. *Proc. Natl. Acad. Sci. U.S.A.* **105**, 16561–16565 (2008).

34. T. Ran, G. Ozorowski, Y. Gao, O. A. Sineshchekov, W. Wang, J. L. Spudich, H. Luecke, Cross-protomer interaction with the photoactive site in oligomeric proteorhodopsin complexes. *Acta*

Crystallogr. D Biol. Crystallogr. **69**, 1965–1980 (2013).

35. A. Chazan, I. Das, T. Fujiwara, S. Murakoshi, A. Rozenberg, A. Molina-Márquez, F. K. Sano, T. Tanaka, P. Gómez-Villegas, S. Larom, A. Pushkarev, P. Malakar, M. Hasegawa, Y. Tsukamoto, T. Ishizuka, M. Konno, T. Nagata, Y. Mizuno, K. Katayama, R. Abe-Yoshizumi, S. Ruhman, K. Inoue, H. Kandori, R. León, W. Shihoya, S. Yoshizawa, M. Sheves, O. Nureki, O. Béjà, Phototrophy by antenna-containing rhodopsin pumps in aquatic environments. *Nature* **615**, 535–540 (2023).

36. S. Reckel, D. Gottstein, J. Stehle, F. Löhr, M.-K. Verhoefen, M. Takeda, R. Silvers, M. Kainosho, C. Glaubitz, J. Wachtveitl, F. Bernhard, H. Schwalbe, P. Güntert, V. Dötsch, Solution NMR Structure of Proteorhodopsin. *Angew. Chem. Int. Ed. Engl.* **50**, 11942–11946 (2011).

37. K. Gao, J. Beardall, D.-P. Häder, J. M. Hall-Spencer, G. Gao, D. A. Hutchins, Effects of ocean acidification on marine photosynthetic organisms under the concurrent influences of warming, UV radiation, and deoxygenation. *Front. Mar. Sci.* **6**, 322 (2019).

38. E. S. Imasheva, S. P. Balashov, J. M. Wang, J. K. Lanyi, pH-dependent transitions in xanthorhodopsin. *Photochem. Photobiol.* **82**, 1406–1413 (2006).

39. S. P. Balashov, L. E. Petrovskaya, E. P. Lukashev, E. S. Imasheva, A. K. Dioumaev, J. M. Wang, S. V. Sychev, D. A. Dolgikh, A. B. Rubin, M. P. Kirpichnikov, J. K. Lanyi, Aspartate-histidine interaction in the retinal schiff base counterion of the light-driven proton pump of Exiguobacterium sibiricum. *Biochemistry* **51**, 5748–5762 (2012).

40. H. Okumura, M. Murakami, T. Kouyama, Crystal structures of acid blue and alkaline purple forms of bacteriorhodopsin. *J. Mol. Biol.* **351**, 481–495 (2005).

41. A. K. Dioumaev, L. S. Brown, J. Shih, E. N. Spudich, J. L. Spudich, J. K. Lanyi, Proton transfers in the photochemical reaction cycle of proteorhodopsin. *Biochemistry* **41**, 5348–5358 (2002).

42. S. Subramanlam, R. Henderson, Molecular mechanism of vectorial proton translocation by bacteriorhodopsin. *Nature* **406**, 653–657 (2000).

43. R. Huber, T. Köhler, M. O. Lenz, E. Bamberg, R. Kalmbach, M. Engelhard, J. Wachtveitl, pH-dependent photoisomerization of retinal in proteorhodopsin. *Biochemistry* **44**, 1800–1806 (2005).

44. M. O. Lenz, R. Huber, B. Schmidt, P. Gilch, R. Kalmbach, M. Engelhard, J. Wachtveitl, First steps of retinal photoisomerization in proteorhodopsin. *Biophys. J.* **91**, 255–262 (2006).

45. D. Ikeda, Y. Furutani, H. Kandori, FTIR study of the retinal Schiff base and internal water molecules of proteorhodopsin. *Biochemistry* **46**, 5365–5373 (2007).

46. M. Mehler, C. E. Eckert, A. J. Leeder, J. Kaur, T. Fischer, N. Kubatova, L. J. Brown, R. C. D. Brown, J. Becker-Baldus, J. Wachtveitl, C. Glaubitz, Chromophore distortions in photointermediates of proteorhodopsin visualized by dynamic nuclear polarization-enhanced solid-state NMR. *J. Am. Chem. Soc.* **139**, 16143–16153 (2017).

47. J. F. Bada Juarez, P. J. Judge, S. Adam, D. Axford, J. Vinals, J. Birch, T. O. C. Kwan, K. K. Hoi, H.-Y. Yen, A. Vial, P.-E. Milhiet, C. V. Robinson, I. Schapiro, I. Moraes, A. Watts, Structures of the archaerhodopsin-3 transporter reveal that disordering of internal water networks underpins receptor sensitization. *Nat. Commun.* **12**, 629 (2021).

48. D. Zabelskii, A. Alekseev, K. Kovalev, V. Rankovic, T. Balandin, D. Soloviov, D. Bratanov, E. Savelyeva, E. Podolyak, D. Volkov, S. Vaganova, R. Astashkin, I. Chizhov, N. Yutin, M. Rulev, A. Popov, A.-S. Eria-Oliveira, T. Rokitskaya, T. Mager, Y. Antonenko, R. Rosselli, G. Armeev, K. Shaitan, M. Vivaudou, G. Büldt, A. Rogachev, F. Rodriguez-Valera, M. Kirpichnikov, T. Moser, A. Offenhäusser, D. Willbold, E. Koonin, E. Bamberg, V. Gordeliy, Viral rhodopsins 1 are an unique family of light-gated cation channels. *Nat. Commun.* **11**, 5707 (2020).

49. J. H. Park, P. Scheerer, K. P. Hofmann, H.-W. Choe, O. P. Ernst, Crystal structure of the ligand-free G-protein-coupled receptor opsin. *Nature* **454**, 183–187 (2008).

50. R. J. C. Hilf, R. Dutzler, Structure of a potentially open state of a proton-activated pentameric ligand-gated ion channel. *Nature* **457**, 115–118 (2009).

51. T. Fujisawa, K. Nishikawa, J. Tamogami, M. Unno, Conformational analysis of a retinal schiff base chromophore in proteorhodopsin by Raman optical activity. *J. Phys. Chem. Lett.* **12**, 9564–

9568 (2021).

52. C. Bamann, E. Bamberg, J. Wachtveitl, C. Glaubitz, Proteorhodopsin. *Biochim. Biophys. Acta Bioenerg.* **1837**, 614–625 (2014).

53. B. Schätzler, N. A. Dencher, J. Tittor, D. Oesterhelt, S. Yaniv-Checover, E. Nachliel, M. Gutman, Subsecond proton-hole propagation in bacteriorhodopsin. *Biophys. J.* **84**, 671–686 (2003).

54. S. Faramarzi, J. Feng, B. Mertz, Allosteric effects of the proton donor on the microbial proton pump Proteorhodopsin. *Biophys. J.* **115**, 1240–1250 (2018).

55. S. Sasaki, J. Tamogami, K. Nishiya, M. Demura, T. Kikukawa, Replaceability of Schiff base proton donors in light-driven proton pump rhodopsins. *J. Biol. Chem.* **297**, 101013 (2021).

56. J. F. Nagle, H. J. Morowitz, Molecular mechanisms for proton transport in membranes. *Proc. Natl. Acad. Sci. U.S.A.* **75**, 298–302 (1978).

57. J. F. Nagle, S. Tristram-Nagle, Hydrogen bonded chain mechanisms for proton conduction and proton pumping. *J. Membr. Biol.* **74**, 1–14 (1983).

58. T. Noji, Y. Chiba, K. Saito, H. Ishikita, Energetics of the H-bond network in *Exiguobacterium sibiricum* rhodopsin. *Biochemistry* **63**, 1505–1512 (2024).

59. E. Freier, S. Wolf, K. Gerwert, Proton transfer via a transient linear water-molecule chain in a membrane protein. *Proc. Natl. Acad. Sci. U.S.A.* **108**, 11435–11439 (2011).

60. M. R. M. Miranda, A. R. Choi, L. Shi, A. G. Bezerra Jr, K.-H. Jung, L. S. Brown, The photocycle and proton translocation pathway in a cyanobacterial ion-pumping rhodopsin. *Biophys. J.* **96**, 1471–1481 (2009).

61. F. Garczarek, K. Gerwert, Functional waters in intraprotein proton transfer monitored by FTIR difference spectroscopy. *Nature* **439**, 109–112 (2006).

62. T. Sugimoto, K. Katayama, H. Kandori, FTIR study of light-induced proton transfer and Ca^{2+}

binding in T82D mutant of TAT rhodopsin. *Biophys. J.* **123**, 4245–4255 (2024).

63. P. Goyal, N. Ghosh, P. Phatak, M. Clemens, M. Gaus, M. Elstner, Q. Cui, Proton storage site in bacteriorhodopsin: New insights from quantum mechanics/molecular mechanics simulations of microscopic pK_a and infrared spectra. *J. Am. Chem. Soc.* **133**, 14981–14997 (2011).

64. D. Maag, T. Mast, M. Elstner, Q. Cui, T. Kubař, O to bR transition in bacteriorhodopsin occurs through a proton hole mechanism. *Proc. Natl. Acad. Sci. U.S.A.* **118**, e2024803118 (2021).

65. A. Shigeta, S. Ito, K. Inoue, T. Okitsu, A. Wada, H. Kandori, I. Kawamura, Solid-state nuclear magnetic resonance structural study of the retinal-binding pocket in sodium ion pump rhodopsin. *Biochemistry* **56**, 543–550 (2017).

66. N. Caramello, A. Royant, From femtoseconds to minutes: Time-resolved macromolecular crystallography at XFELs and synchrotrons. *Acta Crystallogr. D Struct. Biol.* **80**, 60–79 (2024).

67. G. Khusainov, J. Standfuss, T. Weinert, The time revolution in macromolecular crystallography. *Struct. Dyn.* **11**, 020901 (2024).

68. I. Gushchin, A. Reshetnyak, V. Borshchevskiy, A. Ishchenko, E. Round, S. Grudinin, M. Engelhard, G. Büldt, V. Gordeliy, Active state of sensory rhodopsin II: Structural determinants for signal transfer and proton pumping. *J. Mol. Biol.* **412**, 591–600 (2011).

69. S. Liu, W. Li, Protein fusion strategies for membrane protein stabilization and crystal structure determination. *Crystals* **12**, 1041 (2022).

70. S. Dai, L.-M. Funk, F. R. von Pappenheim, V. Sautner, M. Paulikat, B. Schröder, J. Uranga, R. A. Mata, K. Tittmann, Low-barrier hydrogen bonds in enzyme cooperativity. *Nature* **573**, 609–613 (2019).

71. H. Ogata, K. Nishikawa, W. Lubitz, Hydrogens detected by subatomic resolution protein crystallography in a [NiFe] hydrogenase. *Nature* **520**, 571–574 (2015).

72. M. Woińska, S. Grabowsky, P. M. Dominiak, K. Woźniak, D. Jayatilaka, Hydrogen atoms can

be located accurately and precisely by X-ray crystallography. *Sci. Adv.* **2**, e1600192 (2016).

73. U. K. Eriksson, G. Fischer, R. Friemann, G. Enkavi, E. Tajkhorshid, R. Neutze, Subangstrom resolution X-ray structure details aquaporin-water interactions. *Science* **340**, 1346–1349 (2013).

74. M. P. Blakeley, S. S. Hasnain, S. V. Antonyuk, Sub-atomic resolution X-ray crystallography and neutron crystallography: Promise, challenges and potential. *IUCrJ* **2**, 464–474 (2015).

75. J. A. Fuhrman, M. S. Schwalbach, U. Stingl, Proteorhodopsins: An array of physiological roles? *Nat. Rev. Microbiol.* **6**, 488–494 (2008).

76. R. Neutze, E. Pebay-Peyroula, K. Edman, A. Royant, J. Navarro, E. M. Landau, Bacteriorhodopsin: A high-resolution structural view of vectorial proton transport. *Biochim. Biophys. Acta* **1565**, 144–167 (2002).

77. K. Inoue, S. Ito, Y. Kato, Y. Nomura, M. Shibata, T. Uchihashi, S. P. Tsunoda, H. Kandori, A natural light-driven inward proton pump. *Nat. Commun.* **7**, 13415 (2016).

78. V. Shevchenko, T. Mager, K. Kovalev, V. Polovinkin, A. Alekseev, J. Juettner, I. Chizhov, C. Bamann, C. Vavourakis, R. Ghai, I. Gushchin, V. Borshchevskiy, A. Rogachev, I. Melnikov, A. Popov, T. Balandin, F. Rodriguez-Valera, D. J. Manstein, G. Bueldt, E. Bamberg, V. Gordeliy, Inward H^+ pump xenorhodopsin: Mechanism and alternative optogenetic approach. *Sci. Adv.* **3**, e1603187 (2017).

79. A. Okuyama, S. Hososhima, H. Kandori, S. P. Tsunoda, Driving forces of proton-pumping rhodopsins. *Biophys. J.* **123**, 4274–4284 (2024).

80. A. Warshel, A. Papazyan, P. A. Kollman, On low-barrier hydrogen bonds and enzyme catalysis. *Science* **269**, 102–106 (1995).

81. S. Yamaguchi, H. Kamikubo, K. Kurihara, R. Kuroki, N. Niimura, N. Shimizu, Y. Yamazaki, M. Kataoka, Low-barrier hydrogen bond in photoactive yellow protein. *Proc. Natl. Acad. Sci. U.S.A.* **106**, 440–444 (2009).

82. J. Wang, Visualization of H atoms in the X-ray crystal structure of photoactive yellow protein: Does it contain low-barrier hydrogen bonds? *Protein Sci.* **28**, 1966–1972 (2019).

83. K. Katoh, D. M. Standley, MAFFT multiple sequence alignment software version 7: Improvements in performance and usability. *Mol. Biol. Evol.* **30**, 772–780 (2013).

84. B. Q. Minh, H. A. Schmidt, O. Chernomor, D. Schrempf, M. D. Woodhams, A. Von Haeseler, R. Lanfear, E. Teeling, IQ-TREE 2: New models and efficient methods for phylogenetic inference in the Genomic Era. *Mol. Biol. Evol.* **37**, 1530–1534 (2020).

85. S. Kalyaanamoorthy, B. Q. Minh, T. K. F. Wong, A. Von Haeseler, L. S. Jermiin, ModelFinder: Fast model selection for accurate phylogenetic estimates. *Nat. Methods* **14**, 587–589 (2017).

86. D. T. Hoang, O. Chernomor, A. Von Haeseler, B. Q. Minh, L. S. Vinh, UFBoot2: Improving the ultrafast bootstrap approximation. *Mol. Biol. Evol.* **35**, 518–522 (2018).

87. R. C. Edgar, MUSCLE: Multiple sequence alignment with high accuracy and high throughput. *Nucleic Acids Res.* **32**, 1792–1797 (2004).

88. K. Okonechnikov, O. Golosova, M. Fursov, UGENE team, Unipro UGENE: A unified bioinformatics toolkit. *Bioinformatics* **28**, 1166–1167 (2012).

89. I. Letunic, P. Bork, Interactive Tree Of Life (iTOL) v5: An online tool for phylogenetic tree display and annotation. *Nucleic Acids Res.* **49**, W293–W296 (2021).

90. F. W. Studier, Protein production by auto-induction in high density shaking cultures. *Protein Expr. Purif.* **41**, 207–234 (2005).

91. L. Renault, B. Guibert, J. Cherfils, Structural snapshots of the mechanism and inhibition of a guanine nucleotide exchange factor. *Nature* **426**, 525–530 (2003).

92. I. Gushchin, V. Shevchenko, V. Polovinkin, K. Kovalev, A. Alekseev, E. Round, V. Borshchevskiy, T. Balandin, A. Popov, T. Gensch, C. Fahlke, C. Bamann, D. Willbold, G. Büldt, E. Bamberg, V. Gordeliy, Crystal structure of a light-driven sodium pump. *Nat. Struct. Mol.*

Biol. **22**, 390–395 (2015).

93. E. Bamberg, H.-J. Apell, N. A. Dencher, W. Sperling, H. Stieve, P. Läuger, Photocurrents generated by bacteriorhodopsin on planar bilayer membranes. *Biophys. Struct. Mech.* **5**, 277–292 (1979).

94. I. Chizhov, D. S. Chernavskii, M. Engelhard, K. H. Mueller, B. V. Zubov, B. Hess, Spectrally silent transitions in the bacteriorhodopsin photocycle. *Biophys. J.* **71**, 2329–2345 (1996).

95. I. Chizhov, G. Schmies, R. Seidel, J. R. Sydor, B. Lüttenberg, M. Engelhard, The photophobic receptor from natronobacterium pharaonis: Temperature and pH dependencies of the photocycle of sensory Rhodopsin II. *Biophys. J.* **75**, 999–1009 (1998).

96. I. Chizhov, M. Engelhard, Temperature and halide dependence of the photocycle of halorhodopsin from *Natronobacterium pharaonis*. *Biophys. J.* **81**, 1600–1612 (2001).

97. D. Bratanov, K. Kovalev, J.-P. Machtens, R. Astashkin, I. Chizhov, D. Soloviov, D. Volkov, V. Polovinkin, D. Zabelskii, T. Mager, I. Gushchin, T. Rokitskaya, Y. Antonenko, A. Alekseev, V. Shevchenko, N. Yutin, R. Rosselli, C. Baeken, V. Borshchevskiy, G. Bourenkov, A. Popov, T. Balandin, G. Büldt, D. J. Manstein, F. Rodriguez-Valera, C. Fahlke, E. Bamberg, E. Koonin, V. Gordeliy, Unique structure and function of viral rhodopsins. *Nat. Commun.* **10**, 4939 (2019).

98. O. Volkov, K. Kovalev, V. Polovinkin, V. Borshchevskiy, C. Bamann, R. Astashkin, E. Marin, A. Popov, T. Balandin, D. Willbold, G. Büldt, E. Bamberg, V. Gordeliy, Structural insights into ion conduction by channelrhodopsin 2. *Science* **358**, eaan8862 (2017).

99. K. Kovalev, D. Volkov, R. Astashkin, A. Alekseev, I. Gushchin, J. M. Haro-Moreno, I. Chizhov, S. Siletsky, M. Mamedov, A. Rogachev, T. Balandin, V. Borshchevskiy, A. Popov, G. Bourenkov, E. Bamberg, F. Rodriguez-Valera, G. Büldt, V. Gordeliy, High-resolution structural insights into the heliorhodopsin family. *Proc. Natl. Acad. Sci. U.S.A.* **117**, 4131–4141 (2020).

100. W. Kabsch, XDS. *Acta Crystallogr. D Biol. Crystallogr.* **66**, 125–132 (2010).

101. S. Günther, P. Y. A. Reinke, Y. Fernández-García, J. Lieske, T. J. Lane, H. M. Ginn, F. H. M.

Koua, C. Ehrt, W. Ewert, D. Oberthuer, O. Yefanov, S. Meier, K. Lorenzen, B. Krichel, J.-D. Kopicki, L. Gelisio, W. Brehm, I. Dunkel, B. Seychell, H. Gieseler, B. Norton-Baker, B. Escudero-Pérez, M. Domaracky, S. Saouane, A. Tolstikova, T. A. White, A. Hänle, M. Groessler, H. Fleckenstein, F. Trost, M. Galchenkova, Y. Gevorkov, C. Li, S. Awel, A. Peck, M. Barthelmess, F. Schlünzen, P. L. Xavier, N. Werner, H. Andaleeb, N. Ullah, S. Falke, V. Srinivasan, B. A. França, M. Schwinzer, H. Brognaro, C. Rogers, D. Melo, J. I. Zaitseva-Kinneberg, J. Knoska, G. E. Peña-Murillo, A. R. Mashhour, V. Hennicke, P. Fischer, J. Hakanpää, J. Meyer, P. Gribbon, B. Ellinger, M. Kuzikov, M. Wolf, A. R. Beccari, G. Bourenkov, D. von Stetten, G. Pompidor, I. Bento, S. Panneerselvam, I. Karpics, T. R. Schneider, M. M. Garcia-Alai, S. Niebling, C. Günther, C. Schmidt, R. Schubert, H. Han, J. Boger, D. C. F. Monteiro, L. Zhang, X. Sun, J. Pletzer-Zelgert, J. Wollenhaupt, C. G. Feiler, M. S. Weiss, E.-C. Schulz, P. Mehrabi, K. Karničar, A. Usenik, J. Loboda, H. Tidow, A. Chari, R. Hilgenfeld, C. Uetrecht, R. Cox, A. Zaliani, T. Beck, M. Rarey, S. Günther, D. Turk, W. Hinrichs, H. N. Chapman, A. R. Pearson, C. Betzel, A. Meents, X-ray screening identifies active site and allosteric inhibitors of SARS-CoV-2 main protease. *Science* **372**, 642–646 (2021).

102. M. Słabicki, Z. Kozicka, G. Petzold, Y.-D. Li, M. Manojkumar, R. D. Bunker, K. A. Donovan, Q. L. Sievers, J. Koeppel, D. Suchyta, A. S. Sperling, E. C. Fink, J. A. Gasser, L. R. Wang, S. M. Corsello, R. S. Sellar, M. Jan, D. Gillingham, C. Scholl, S. Fröhling, T. R. Golub, E. S. Fischer, N. H. Thomä, B. L. Ebert, The CDK inhibitor CR8 acts as a molecular glue degrader that depletes cyclin K. *Nature* **585**, 293–297 (2020).

103. L. Andreeva, B. Hiller, D. Kostrewa, C. Lässig, C. C. De Oliveira Mann, D. Jan Drexler, A. Maiser, M. Gaidt, H. Leonhardt, V. Hornung, K.-P. Hopfner, cGAS senses long and HMGB/TFAM-bound U-turn DNA by forming protein–DNA ladders. *Nature* **549**, 394–398 (2017).

104. Q. L. Sievers, G. Petzold, R. D. Bunker, A. Renneville, M. Słabicki, B. J. Liddicoat, W. Abdulrahman, T. Mikkelsen, B. L. Ebert, N. H. Thomä, Defining the human C2H2 zinc finger degrome targeted by thalidomide analogs through CRBN. *Science* **362**, eaat0572 (2018).

105. P. Evans, Scaling and assessment of data quality. *Acta Crystallogr. D Biol. Crystallogr.* **62**,

72–82 (2006).

106. A. Vagin, A. Teplyakov, Molecular replacement with MOLREP. *Acta Crystallogr. D Biol. Crystallogr.* **66**, 22–25 (2010).

107. I. Melnikov, V. Polovinkin, K. Kovalev, I. Gushchin, M. Shevtsov, V. Shevchenko, A. Mishin, A. Alekseev, F. Rodriguez-Valera, V. Borshchevskiy, V. Cherezov, G. A. Leonard, V. Gordeliy, A. Popov, Fast iodide-SAD phasing for high-throughput membrane protein structure determination. *Sci. Adv.* **3**, e1602952 (2017).

108. J. C. Amor, D. H. Harrison, R. A. Kahn, D. Ringe, Structure of the human ADP-ribosylation factor 1 complexed with GDP. *Nature* **372**, 704–708 (1994).

109. T. C. Terwilliger, R. W. Grosse-Kunstleve, P. V. Afonine, N. W. Moriarty, P. H. Zwart, L. W. Hung, R. J. Read, P. D. Adams, Iterative model building, structure refinement and density modification with the PHENIX AutoBuild wizard. *Acta Crystallogr. D Biol. Crystallogr.* **64**, 61–69 (2007).

110. P. Emsley, B. Lohkamp, W. G. Scott, K. Cowtan, Features and development of Coot. *Acta Crystallogr. D Biol. Crystallogr.* **66**, 486–501 (2010).

111. G. N. Murshudov, P. Skubák, A. A. Lebedev, N. S. Pannu, R. A. Steiner, R. A. Nicholls, M. D. Winn, F. Long, A. A. Vagin, REFMAC5 for the refinement of macromolecular crystal structures. *Acta Crystallogr. D Biol. Crystallogr.* **67**, 355–367 (2011).

112. P. V. Afonine, R. W. Grosse-Kunstleve, N. Echols, J. J. Headd, N. W. Moriarty, M. Mustyakimov, T. C. Terwilliger, A. Urzhumtsev, P. H. Zwart, P. D. Adams, Towards automated crystallographic structure refinement with phenix.refine. *Acta Crystallogr. D Biol. Crystallogr.* **68**, 352–367 (2012).

113. C. J. Williams, J. J. Headd, N. W. Moriarty, M. G. Prisant, L. L. Videau, L. N. Deis, V. Verma, D. A. Keedy, B. J. Hintze, V. B. Chen, S. Jain, S. M. Lewis, W. B. Arendall III, J. Snoeyink, P. D. Adams, S. C. Lovell, J. S. Richardson, D. C. Richardson, MolProbity: More and better reference data for improved all-atom structure validation. *Protein Sci.* **27**, 293–315 (2018).

114. K. Diederichs, P. A. Karplus, Better models by discarding data? *Acta Crystallogr. D Biol. Crystallogr.* **69**, 1215–1222 (2013).

115. B. K. Ho, F. Gruswitz, HOLLOW: Generating accurate representations of channel and interior surfaces in molecular structures. *BMC Struct. Biol.* **8**, 49 (2008).

116. M. A. Lomize, I. D. Pogozheva, H. Joo, H. I. Mosberg, A. L. Lomize, OPM database and PPM web server: Resources for positioning of proteins in membranes. *Nucleic Acids Res.* **40**, D370–D376 (2012).

117. E. De Zitter, N. Coquelle, P. Oeser, T. R. M. Barends, J.-P. Colletier, Xtrapol8 enables automatic elucidation of low-occupancy intermediate-states in crystallographic studies. *Commun. Biol.* **5**, 640 (2022).

118. D. W. Cruickshank, Remarks about protein structure precision. *Acta Crystallogr. D Biol. Crystallogr.* **55**, 583–601 (1999).

119. G. A. J. Jeffrey, G. A. Jeffrey, *An Introduction to Hydrogen Bonding* (Oxford Univ. Press, 1997).

120. P. A. Frey, *Encyclopedia of Biological Chemistry* (Elsevier, 2004).

121. D. von Stetten, T. Giraud, P. Carpentier, F. Sever, M. Terrien, F. Dobias, D. H. Juers, D. Flot, C. Mueller-Dieckmann, G. A. Leonard, D. de Sanctis, A. Royant, In crystallo optical spectroscopy (icOS) as a complementary tool on the macromolecular crystallography beamlines of the ESRF. *Acta Crystallogr. D Biol. Crystallogr.* **71**, 15–26 (2015).

122. S. O. Smith, J. Lugtenburg, R. A. Mathies, Determination of retinal chromophore structure in bacteriorhodopsin with resonance Raman spectroscopy. *J. Membr. Biol.* **85**, 95–109 (1985).

123. T. Kouyama, K. Ihara, Existence of two substates in the O intermediate of the bacteriorhodopsin photocycle. *Biochim. Biophys. Acta Biomembr.* **1864**, 183998 (2022).

124. J. Heberle, Proton transfer reactions across bacteriorhodopsin and along the membrane.

Biochim. Biophys. Acta **1458**, 135–147 (2000).

125. H. Kandori, Hydration switch model for the proton transfer in the Schiff base region of bacteriorhodopsin. *Biochim. Biophys. Acta* **1658**, 72–79 (2004).

126. Z. Cao, Y. Peng, T. Yan, S. Li, A. Li, G. A. Voth, Mechanism of fast proton transport along one-dimensional water chains confined in carbon nanotubes. *J. Am. Chem. Soc.* **132**, 11395–11397 (2010).

127. D. W. Deamer, Proton permeation of lipid bilayers. *J. Bioenerg. Biomembr.* **19**, 457–479 (1987).
